# β-Arrestin2 Is Critically Involved in the Differential Regulation of Phosphosignaling Pathways by Thyrotropin-Releasing Hormone and Taltirelin

**DOI:** 10.3390/cells11091473

**Published:** 2022-04-27

**Authors:** Zdenka Drastichova, Radka Trubacova, Jiri Novotny

**Affiliations:** Department of Physiology, Faculty of Science, Charles University, 128 00 Prague, Czech Republic; zdenka.drastichova@natur.cuni.cz (Z.D.); radka.moravcova@natur.cuni.cz (R.T.)

**Keywords:** β-arrestin2, GH1 cells, small GTPase-mediated signaling, taltirelin, thyrotropin-releasing hormone, TRH receptor

## Abstract

In recent years, thyrotropin-releasing hormone (TRH) and its analogs, including taltirelin (TAL), have demonstrated a range of effects on the central nervous system that represent potential therapeutic agents for the treatment of various neurological disorders, including neurodegenerative diseases. However, the molecular mechanisms of their actions remain poorly understood. In this study, we investigated phosphosignaling dynamics in pituitary GH1 cells affected by TRH and TAL and the putative role of β-arrestin2 in mediating these effects. Our results revealed widespread alterations in many phosphosignaling pathways involving signal transduction via small GTPases, MAP kinases, Ser/Thr- and Tyr-protein kinases, Wnt/β-catenin, and members of the Hippo pathway. The differential TRH- or TAL-induced phosphorylation of numerous proteins suggests that these ligands exhibit some degree of biased agonism at the TRH receptor. The different phosphorylation patterns induced by TRH or TAL in β-arrestin2-deficient cells suggest that the β-arrestin2 scaffold is a key factor determining phosphorylation events after TRH receptor activation. Our results suggest that compounds that modulate kinase and phosphatase activity can be considered as additional adjuvants to enhance the potential therapeutic value of TRH or TAL.

## 1. Introduction

Thyrotropin-releasing hormone (TRH) is a tripeptide consisting of pyroglutamyl-histidyl-proline amide. It is produced by neurons in the hypothalamus and circulates through the hypophyseal portal system to the anterior pituitary, where it binds to its receptors and stimulates the release of other hormones [1,2]. TRH is thus a fundamental element involved in the regulation of many hormonal and metabolic functions. TRH has been reported to exert numerous beneficial effects due to its antiapoptotic [3,4,5] or neuroprotective [6,7] properties. This peptide is also considered a potential therapeutic agent for the treatment of neurodegenerative diseases, especially Parkinson’s and Alzheimer´s disease [8]. Because of TRH’s short half-life in the bloodstream, low lipophilicity, long time to cross the blood brain barrier, and potent HPT axis-stimulating effects [9,10], a number of TRH analogs have been synthesized [11]. Among them, the superagonist taltirelin (TAL) seems to be a promising drug due to its much longer half-life and duration of action [12,13]. TAL has a 10–100 times stronger stimulatory effect on the central nervous system and an eight times longer time of action than TRH and was approved for the treatment of spinocerebellar degeneration [10].

The effects of TRH and TAL are mediated by the thyrotropin-releasing hormone receptor (TRH-R), which belongs to family A of G protein-coupled receptors (GPCRs). Two subtypes of TRH-R (TRH-R1 and TRH-R2) have been detected in rodents. TRH-R1 is highly expressed in the pituitary gland, whereas TRH-R2 is not detected or is detected at low levels in the anterior pituitary and was absent in the neurointermediate lobe of the pituitary [14,15]. TRH-R2 exhibits higher basal signaling activity and is internalized more rapidly than TRH-R1. Its long-term activation with TRH results in a lower induction of transcription of the reporter gene [15]. Agonist-bound TRH-R activates phospholipase Cβ via Gq/11 proteins, which subsequently triggers the hydrolysis of phosphatidylinositol 4,5-bisphosphate into two second messengers: inositol 1,4,5-trisphosphate (IP3) and diacylglycerol (DAG) [16]. IP3 then moves away from the membrane, binds to IP3 receptors on the endoplasmic reticulum, and allows the release of Ca^2+^ into the cytosol, where it modulates various signaling pathways. DAG remains near the cell membrane and activates protein kinase C [17]. Other signaling pathways triggered by TRH include MAPK signaling [18], phosphoinositide 3-kinase (PI3K)/Akt signaling [8] and Rho and Ras signaling [19,20].

It has been well-established that TRH-R is phosphorylated and binds to β-arrestin after agonist exposure [21]. β-Arrestins are cytosolic proteins that are among the most important regulatory molecules. Upon binding to a phosphorylated receptor, β-arrestins serve as adapters that link receptor molecules to components of the endocytic apparatus formed by clathrin and adapter protein-2, thereby arresting the signaling and acting as a negative regulator. This is consistent with the original idea describing the function of β-arrestins as key molecules for receptor internalization. There are two β-arrestin isoforms, β-arrestin1 and β-arrestin2, which are ubiquitously expressed [22] and able to interact with TRH-R [21]. β-Arrestins are also associated with a very hot topic in current molecular pharmacology called biased agonism. This term describes the process by which different agonists can activate different signaling pathways through one type of receptor by stabilizing different receptor conformations. This basically means functional selectivity of a particular ligand for one of two signaling pathways that lead either through G proteins or β-arrestin. This mechanism has been demonstrated for several GPCRs, including those coupled to Gq/11 [23,24,25]. However, to date, there is no direct evidence for biased agonism at TRH-R.

Currently, it is clear that the role of β-arrestins is much more diverse. These molecules can have a number of signaling functions, for example, they link Src kinases to receptors [26] or serve as scaffolds for binding individual components of mitogen-activated protein kinase (MAPK) cascades, such as Raf-1, MEK1, and ERK1/2 [27,28,29]. Interestingly, it appears that TRH-R does not require the recruitment of β-arrestin to activate MAPK signaling pathways [18]. The list of other proteins that interact with β-arrestin includes regulators of small GTPase activity, components of the JNK and p38 pathways, casein kinase 2, PI3K and components of the Wnt/β-catenin pathway [30]. It is worth noting that these interactions can be strongly influenced by phosphorylation. The phosphorylation state of Src and the dishevelled proteins DVL1 and DVL2 determines their interactions with β-arrestin [30]. Protein phosphorylation is among the regulatory mechanisms that determine not only protein interactions but also their activation/deactivation, stability and subcellular localization. Phosphorylation and dephosphorylation events are mediated by kinases and phosphatases, respectively, and occur at serine (Ser), threonine (Thr), or tyrosine (Tyr) residues. Most kinases act on both serine and threonine residues (Ser/Thr kinases (STKs)), others act on tyrosine (Tyr kinases (TKs)), and some act on all three residues (dual-specificity kinases (DSKs)) [31].

It is known that β-arrestin2 regulates many signaling pathways in a positive or negative manner [30]. GPCR-dependent activation of Rho kinase signaling increased the association of arrestin with PTEN phosphatase, resulting in the recruitment of PTEN to the plasma membrane, its activation, and negative regulation of Akt signaling [30]. β-Arrestin negatively regulates PAR2 receptor-mediated Cdc42 activation [30]. It positively regulates assembly of the PP2A-Akt-GSK3β complex and subsequent PI3K/Akt-dependent survival signaling or positively mediates Rac1 activation through Wnt5A [30].

There is some evidence that certain small GTPases may also be involved in TRH-R-mediated signaling [32]. Small GTPases are GTP-binding proteins that are divided into five subfamilies: Ras (Ras, Ral, Rap), Rho (Rho, Rac, Cdc42), Rab, Arf, and Ran [30]. The activity of small GTPases is regulated by guanine nucleotide exchange factors (GEFs) and GTPase activator proteins (GAPs). While GEFs stimulate the exchange of GDP for GTP to activate the small GTPases, GAPs promote GTP hydrolysis to inactivate them [33]. Most of the 150 identified GEFs and GAPs are expressed in the brain with specific spatial and temporal expression patterns, but their functions have not been fully elucidated [34]. Small GTPases and their regulators are modified by phosphorylation, which controls their stability and activity, subcellular localization, and interactions with binding partners [35,36,37].

In the present study, we investigated the phosphosignaling dynamics in rat pituitary GH1 cells treated with TRH or TAL. Moreover, our studies revealed a specific role of β-arrestin2 in determining phosphorylation events induced by TRH or TAL.

## 2. Materials and Methods

### 2.1. Materials

The GH1 cell line (CCL 82) was purchased from the American Tissue Type Collection (Rockville, MD, USA). Horse and fetal bovine sera were from Gibco (Carlsbad, CA, USA), LipofectamineTM RNAiMAX and β-arrestin2 antibody were from Invitrogen (Carlsbad, CA, USA). All others chemicals were purchased from Sigma-Aldrich (St. Louis, MO, USA) and were of the highest purity available.

### 2.2. Cell Culture, siRNA Inhibition and Drug Treatment

GH1 cells (ATCC CCL-82), the tumor cells from pituitary gland of Rattus norvegicus, were routinely grown in Ham’s F-12 Nutrient Mixture supplemented with 15% horse serum, 2.5% fetal bovine serum (FBS) and 1% antibiotic-antimycotic solution at 37 °C in 5% CO_2_ humidified atmosphere. Before starting the experiment, growth medium was replaced with medium supplemented with 1% FBS.

For transfection with siRNA, cells were grown in 75 cm, 2 tissue culture flasks, each sample in triplicate. At 50% confluence, cells were transfected with siRNA against β-arrestin2 or control siRNA using Lipofectamine^TM^ RNAiMAX reagent according to the manufacturer´s instructions. Briefly, control or β-arrestin2 siRNA were mixed with an appropriate amount of Lipofectamine RNAiMAX in serum-free Opti-MEM medium and incubated for 5 min at room temperature to obtain siRNA-lipid complexes which were then added to each flask.

Then, 2 days after the transfection, cells were treated with or without 1 μM TRH or 1 μM taltirelin (TAL) to ensure that a maximal response to stimulation was elicited. After 30 min of incubation in the absence (control) or presence of ligands, cells were washed with phosphate-buffered saline (PBS) and subsequently harvested by centrifugation using Hettich Universal 320 centrifuge (1800 rpm, 10 min, 4 °C). Cell pellets were snap-frozen in liquid nitrogen and stored at −80 °C until use.

### 2.3. Western Blot Analysis

Harvested cells were resuspended in TMES buffer (250 mM sucrose, 20 mM Tris-HCl, 1 mM EDTA and 3 mM MgCl_2_; pH 7.0) and briefly sonicated. Homogenates were solubilized in Laemmli buffer and separated in 10% polyacrylamide gels using the Mini-Protean II apparatus (Bio-Rad, Hercules, CA, USA). The resolved proteins were electrotransferred from the gel to a nitrocellulose membrane and stained with Ponceau S (0.1% (*w*/*v*) in 5% acetic acid (*v*/*v*)) to verify equivalent protein loading and normalize the data. After blocking with 5% reduced-fat milk in TBS-T buffer (10 mM Tris, 150 mM NaCl, 0.1% Tween 20 (*v*/*v*); pH 8.0), membranes were incubated with primary antibodies against β-arrestin1 or β-arrestin2 at 4 °C overnight. The next day, membranes were washed three times with TBS-T buffer, probed with secondary antibody conjugated to horseradish peroxidase for 1 h at room temperature and washed three times with TBS-T buffer. Blots were visualized with the enhanced chemiluminescence technique using Thermo Scientific SuperSignal West Dura Extended Duration Substrate according to the manufacturer´s instructions. The generated images were scanned, and the signal intensities of the bands corresponding to β-arrestin1 or β-arrestin2 were quantified using ImageJ software (https://imagej.nih.gov/ij/, assessed on 15 May 2021). Data were analyzed using GraphPad Prism 8.0 (GraphPad Sofware, La Jolla, CA, USA). Results are presented as the mean ± standard error of the mean (SEM) from three independent experiments. Significant differences between groups were determined using Student´s t-test. Statistical significance was set as *p* < 0.05.

### 2.4. Protein Digestion

Cells (~500 µg per sample) were homogenized by sonication using a micro probe sonicator (Bandelin Sonoplus) and lysed by boiling at 95 °C for 10 min in 100 mM Tris pH 8.5 containing 2% sodium deoxycholate. Chloroacetamide and Tris(2-carboxyethyl)phosphine to 40 mM and 10 mM final concentration, respectively, were added. The protein concentration was determined using the BCA protein assay kit (Thermo) and 250 µg of protein per sample was used for MS sample preparation. Proteins were digested by 5 µg of trypsin per sample at 37 °C overnight. Phosphopeptides were enriched using TiO_2_ according to Humphrey et al. [38].

### 2.5. Phosphoproteomic Analysis by nLC-MS2

Nano reversed-phase PepMap C18 chromatography columns (EASY-Spray column, 50 cm × 75 µm ID, 2 µm particles, 100 Å pore size) were used for LC/MS analysis. The buffer of mobile phase A consisted of 0.1% formic acid dissolved in water, and mobile phase B consisted of 0.1% formic acid dissolved in acetonitrile. The loading buffer consisting of 2% acetonitrile, 0.1% trifluoroacetic acid, and water was used to load the samples onto the C18 PepMap100 trap column (5 μm particle size, 300 μm × 5 mm, Thermo Scientific (Waltham, MA, USA)), and sample loading proceeded at 18 μL/min for 4 min. The gradient of mobile phase B from 2% to 35% B was used to elute the peptides from the column. The eluted peptide cations were converted to gas-phase ions by electrospray ionization and then analyzed using a Thermo Orbitrap Fusion (Q-OT-qIT, Thermo Scientific). The Orbitrap was set to a resolution of 120 K (at 200 *m*/*z*) with an ion count of 1 × 10^6^ to perform survey scans of peptide precursors from 350 to 1400 *m*/*z*. Tandem MS was performed by isolation at 1.5 Th with the quadrupole, higher energy collisional dissociation fragmentation with a normalized collision energy of 35, and rapid scan MS analysis in the ion trap. The maximum injection time was 150 ms, while the MS2 ion count target was set to 10^4^. For MS2, only the precursors with charge states 2–6 were scanned. The dynamic exclusion time was set to 30 s with a tolerance of 10 ppm for the selected precursor and its isotopes. The cycle time was set to 2 s and the selection of the monoisotopic precursor was activated during the measurement.

### 2.6. Data Analysis

MaxQuant software (version 1.6.3.4) was used to analyze and quantify the raw data [39]. The false discovery rate (FDR) was set to 1% for both peptides and proteins, while the minimum peptide length was set to seven amino acids. The Rattus norvegicus database was used to search the MS/MS spectra using the Andromeda search engine. Enzyme specificity was set for C-terminal Arg and Lys residues with cleavage at proline bonds. In parallel, a maximum of two failed cleavages were allowed. While carbamidomethylation of cysteine was selected as a fixed modification, N-terminal protein acetylation and methionine oxidation were considered as variable modifications. We used the “match between runs” feature of MaxQuant to transfer identifications based on their masses and retention times (deviation not exceeding 0.7 min) to other LC-MS/MS runs. This approach was also used for quantification experiments. Quantifications were performed using the label-free algorithm in MaxQuant and the Perseus 1.6.1.3 software was used for data analysis [40,41].

Only phosphosites of phosphoproteins whose fold change values were greater than or equal to ±2 and whose intensities were simultaneously detected in at least two of three independent samples were considered for analysis. The differences between the mean values of the intensities of the relevant groups were analyzed using GraphPad Prism 8. The significance level was set at *p* ≤ 0.05. Phosphoproteins that met our above requirements were further analyzed using the Database for Annotation, Visualization, and Integrated Discovery (DAVID). This bioinformatics database allowed the assignment of proteins to specific biological processes. From this database, the processes involved in signaling transduction associated with small GTPases and MAP kinases were selected and mapped using the String database.

## 3. Results

### 3.1. GO Enrichment Analysis of Differentially Phosphorylated Proteins

The aim of this study was to determine how TRH or its analog TAL might affect the phosphosignalosome in the context of biological processes and protein interactions in the pituitary gland, which TRH acts on directly to stimulate thyroid-stimulating hormone production. To this end, we used a rat pituitary tumor cell line (GH1) as a model. The first specific objective was to determine which phosphosignaling pathways are affected by TRH or TAL and what kind of differential changes in phosphosignaling dynamics underlying biased agonism for TRH-R are induced by the two ligands. The next objective was to determine the role of β-arrestin2 in regulating TRH- and TAL-induced phosphosignaling pathways. In the Results section, we focus on the detailed description of the changes induced by TRH or TAL in wild-type or β-arrestin2-deficient cells for each pathway found, including the analysis of the localization and function of some phosphosites. Some descriptive results seen in the figures are mentioned in the Supplementary Results. In the Discussion section, we attempted to relate the results of the different phosphosignaling pathways to more general conclusions to highlight phosphoproteins with significant variations.

To confirm the efficacy of siRNA targeting β-arrestin2, a Western blot analysis was performed with specific antibodies against β-arrestin2 (Figure S1). This analysis showed that transfection of GH1 cells with β-arrestin2 resulted in a reduction in β-arrestin2 expression by more than 60%, without significant parallel change in β-arrestin1 expression. Bioinformatic analysis of data obtained by label-free bottom-up mass spectrometry-based proteomics was performed using the MaxQuant and Perseus software platforms. Differences in phosphoproteomes of control or ligand-treated GH1 cells were sorted as qualitative and quantitative changes. A qualitative change was defined as evidence of presence or absence if certain proteins were detected in only one experimental group from the pairwise comparison and in at least two measured samples of the biological triplicates. A quantitative change was defined as at least twofold difference between two experimental groups and simultaneous detection of phosphopeptides in at least two measured samples of the biological triplicates. To specify the biological processes affected by knockdown of β-arrestin2 and/or treatment with TRH or TAL, the sets of altered phosphoproteins for each experimental group were examined in Gene Ontology (GO) enrichment analysis using the DAVID database v6.8 (DAVID Functional Annotation Bioinformatics Microarray Analysis, ncifcrf.gov, assessed on 5 November 2021). The GO terms enriched in the experimental groups are related to activation and regulation of GTPase activity (p-values ranging from 1.40 × 10^−17^ to 4.90 × 10^−2^), protein and peptidyl-serine phosphorylation, and the MAPK cascade (*p*-values ranging from 1.40 × 10^−6^ to 4.60 × 10^−2^).

### 3.2. Alterations in Phosphorylation of Phosphoproteins Involved in GTPase-Mediated Signal Transduction and Protein Phosphorylation

The data sets of differentially phosphorylated proteins associated with small GTPase activity and protein phosphorylation consist of 571 phosphosites from 237 phosphoproteins in GH1 cells after siRNA knockdown of β-arrestin2 compared with control GH1 cells (pairwise comparison marked Arr/C) (Tables S1 and S6), 216 phosphosites of 113 phosphoproteins in GH1 cells after stimulation with 1 μM TRH compared with control GH1 cells (TRH/C) (Tables S2 and S7), 231 phosphosites of 131 phosphoproteins in GH1 cells after siRNA knockdown of β-arrestin2 and stimulation with 1 μM TRH compared with GH1 cells after siRNA knockdown of β-arrestin2 (Arr-TRH/Arr) (Tables S4 and S9), 305 phosphosites of 152 phosphoproteins in GH1 cells after stimulation with 1 μM TAL compared with control GH1 cells (TAL/C) (Tables S3 and S8), and 290 phosphosites of 157 phosphoproteins in GH1 cells after siRNA knockdown of β-arrestin2 and stimulation with 1 μM TAL compared with GH1 cells after siRNA knockdown of β-arrestin2 (Arr-TAL/Arr) (Tables S5 and S10). According to the UniProt database, many phosphorylations were found in disordered regions of proteins. Only some of them were found in functional or binding domains and are directly mentioned below.

**Figure 1:**
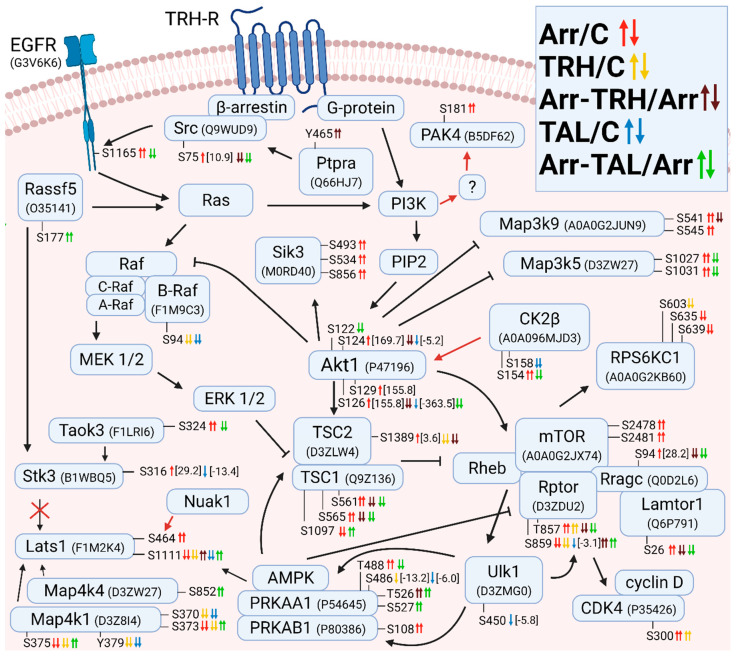
Alterations in phosphorylation of phosphoproteins involved in Ras GTPase-mediated signal transduction associated with the PI3K/Akt/mTOR pathway. Phosphoproteins with changes in phosphorylation after β-arrestin2 knockdown and TRH or TAL treatments in wild-type or β-arrestin2 knockdown cells are labeled with the gene name and protein ID in the UniProt Database (www.uniprot.org, accessed on 1 August 2021). Phosphosites are labeled with the abbreviations of the amino acid residues (S, serine; T, threonine; Y, tyrosine) and the number that determines the position in the amino acid sequence of the protein. Small up and down arrows represent an increase and a decrease in phosphorylation, respectively. Two or one arrows represent qualitative and quantitative changes, respectively. The five pairwise experimental groups are distinguished by color (β-arrestin2 knockdown to control, red; TRH to control, yellow; β-arrestin2 knockdown + TRH to control, brown; TAL to control, blue; β-arrestin2 knockdown + TAL to control, green). Associations between proteins are shown by arrows that determine stimulatory or inhibitory effects. Red arrows and crosses represent confirmed relationships between phosphorylation on the protein phosphosite and the phosphorylating kinase or downstream protein. Associations and interactions between proteins were ordered according to [44,45,46,47,48,50,52,54,55,56,57,58,59,60,61,62,63,64,65,66,67,68,69,70,71,72]. Abbreviations: AMPK: 5′-AMP-activated protein kinase; CDK4: cyclin-dependent kinase 4; CK2β: casein kinase 2β; EGFR: epidermal growth factor receptor; ERK1/2: extracellular signal-regulated kinase 1; GPCR: G-protein coupled receptor; Map3k: mitogen-activated protein kinase kinase kinase; Map4k: mitogen-activated protein kinase kinase kinase kinase; MEK1/2: dual specificity mitogen-activated protein kinase kinase 1; mTOR: mechanistic target of rapamycin; PAK4: serine/threonine p21-activated kinase 4; PIP2: phosphatidylinositol 4,5-bisphosphate; PI3K: phosphoinositide 3-kinase; RPS6KC1: ribosomal protein S6 kinase C1; Stk3: serine/threonine protein kinase 3; Rptor: regulatory-associated protein of mTOR; Rragc: Ras-related GTP-binding protein C; Src: proto-oncogene tyrosine-protein kinase Src; Taok3: serine/threonine protein kinase thousand and one amino acid protein 3; TSC: tuberous sclerosis protein; Ulk1: Unc-51-like kinase 1.

**Figure 2:**
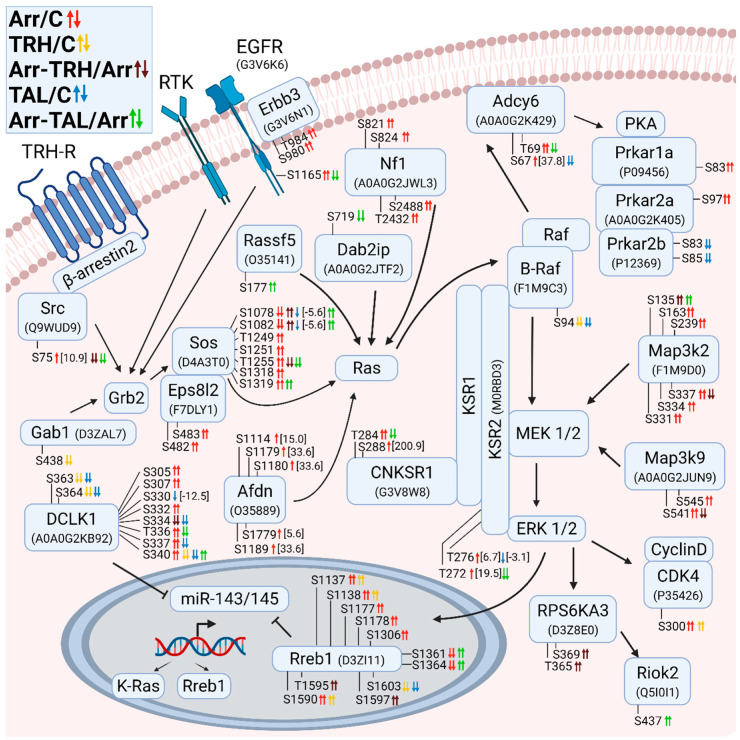
Alterations in phosphorylation of phosphoproteins involved in Ras GTPase-mediated signal transduction associated with Grb2/Sos/Ras/Raf/MEK/ERK. The description of the figure is the same as in Figure 1. Associations and interactions between proteins were ordered according to [60,64,65,76,79,80,84,85,86,87,88,89]. Abbreviations: Afdn: afadin; Adcy6: adenylyl cyclase 6; Cnksr1: connector enhancer of kinase suppressor 1; Dab2ip: disabled homolog 2-interacting protein; Dclk1: doublecortin-like kinase 1; Eps8l2: epidermal growth factor receptor kinase substrate 8-like protein 2; Erbb3: receptor tyrosine-protein kinase erbB-3; Gab1: Grb2-associated-binding protein 1; Grb2: growth factor receptor-bound protein2; KSR: kinase suppressor of Ras; PKA: cAMP-dependent protein kinase A; Prkar: cAMP-dependent protein kinase regulatory subunit; Rassf5: Ras association domain-containing protein 5; Nf1: neurofibromin 1; Riok2: serine/threonine protein kinase RIO2; RPS6KA3: ribosomal protein S6 kinase α3; Rreb1: Ras-responsive element-binding protein 1; Sos: son of sevenless homolog 1.

**Figure 3:**
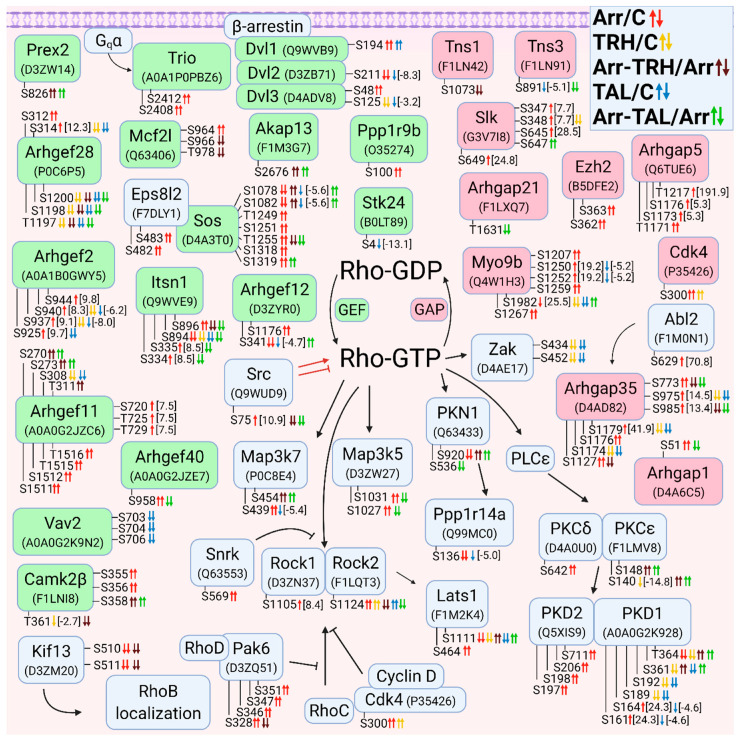
Alterations in phosphorylation of phosphoproteins involved in Rho GTPase-mediated signal transduction. The description of the figure is the same as in Figure 1. Associations and interactions between proteins were ordered according to [44,69,91,92,93,94,95,96,97,98,99,100,101,102,103,104,105,106,107,108,109,110,111,112,113,114,115,116,117]. Abbreviations: Abl2: Abelson tyrosine-protein kinase 2; Akap13: A-kinase anchor protein 13; Arhgef: Rho guanine nucleotide exchange factor; Arhgap: Rho GTPase-activating protein; Camk2β: calcium/calmodulin-dependent protein kinase type II subunit β; Dvl: dishevelled protein; Ezh2: histone-lysine N-methyltranferase; Kif13: kinesin family member 13A; Mcf2l: Mcf2-transforming sequence-like protein; Myo9b: unconventional myosin-IXb; Itsn: intersectin; Pak6, p21-activated kinase 6; PKN1: serine/threonine-protein kinase N1; PLCε: 1-phosphatidylinositol 4,5-bisphosphate phosphodiesterase ε; PKCδ: protein kinase Cδ; PKCε: protein kinase Cε; PKD: serine/threonine-protein kinase D; Ppp1r14a: protein phosphatase 1 regulatory subunit 14A; Ppp1r9b: neurabin-2; Prex2: phosphatidylinositol 3,4,5-trisphosphate-dependent Rac exchanger 2 protein; Rock: Rho-associated protein kinase; Slk: STE20-like serine/threonine-protein kinase; Snrk: SNF-related serine/threonine-protein kinase; Stk24: serin/threonine-protein kinase 24; Tns: tensin; Trio: Triple functional domain protein; Zak: leucine zipper- and sterile α motif-containing kinase.

**Figure 4:**
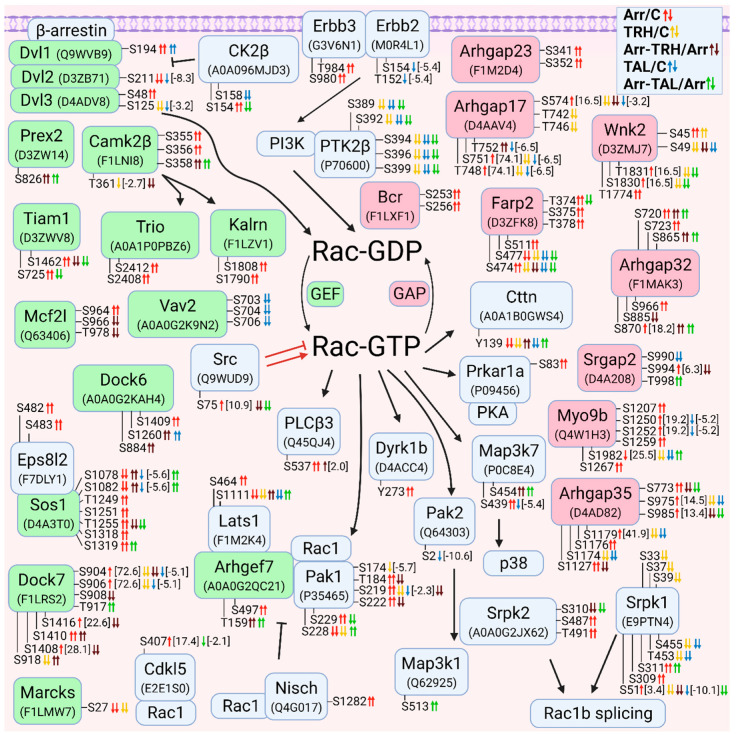
Alterations in phosphorylation of phosphoproteins involved in Rac GTPase-mediated signal transduction. The description of the figure is the same as in Figure 1. Associations and interactions between proteins were ordered according to [44,72,94,98,100,102,110,111,114,117,118,119,121,122,123,124,125,126,127,128,129,130,131,132,133,134,135,136,137,138,139,140,141,142,143,144,145]. Abbreviations: Cdkl5: cyclin-dependent kinase-like 5; Cttn: Src substrate cortactin; Dock: dedicator of cytokinesis protein; Dyrk1b: dual specificity tyrosine-phosphorylation-regulated kinase 1B; Farp2: FERM, ARH/RhoGEF and pleckstrin domain protein 2; Kalrn: kalirin; Marcks, myristoylated alanine-rich C-kinase substrate; Nisch: nischarin; Pak1: p21-activated kinase 1; Pak2: p21-activated kinase 2; PLCβ3: 1-phosphatidylinositol 4,5-bisphosphate phosphodiesterase β3; Ptk2β: protein-tyrosine kinase 2β; Srgap2: SLIT-ROBO Rho GTPase-activating protein 2; Srpk: SRSF protein kinase; Tiam1: TIAM Rac1-associated GEF 1; Wnk2: serine/threonine-protein kinase with no lysine 2.

**Figure 5:**
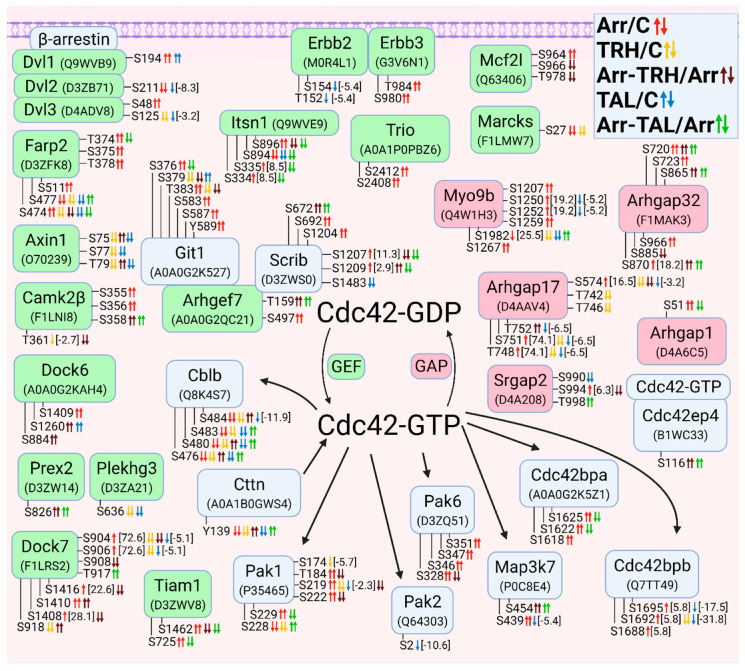
Alterations in phosphorylation of phosphoproteins involved in Cdc42 GTPase-mediated signal transduction. The description of the figure is the same as in Figure 1. Associations and interactions between proteins were arranged according to [94,101,107,111,114,117,121,124,128,129,133,137,140,144,145,150,151,152,153,154,155,156,157]. Abbreviations: Cblb: E3 ubiquitin-protein ligase CBL-B; Cdc42bpa: Cdc42-binding protein kinase α; Cdc42bpb: Cdc42-binding protein kinase β; Cdc42ep4: Cdc42 effector protein 4; Git1: ARF GTPase-activating protein GIT1; Plekhg3: pleckstrin homology domain-containing family G member 3; Scrib: protein scribble homolog.

**Figure 6:**
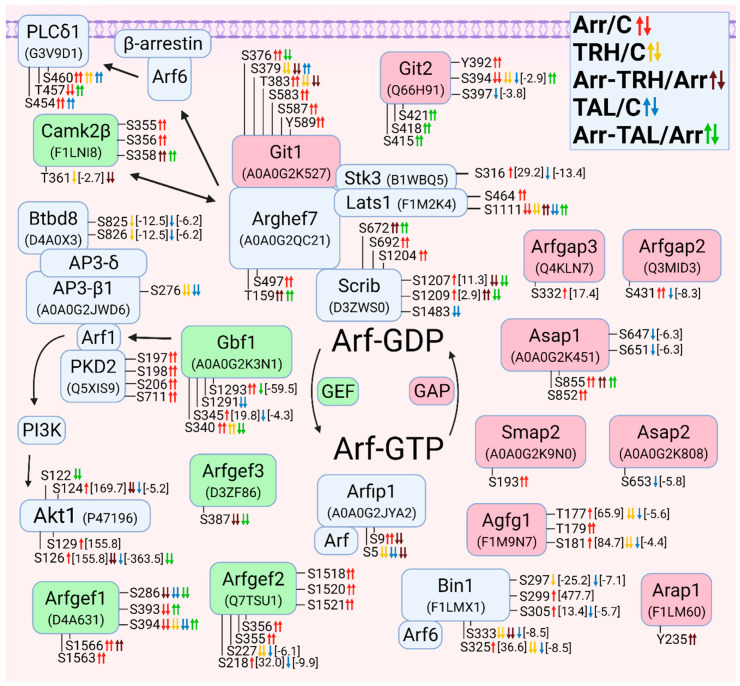
Alterations in phosphorylation of phosphoproteins involved in Arf GTPase-mediated signal transduction. The description of the figure is the same as in Figure 1. Associations and interactions between proteins were ordered according to [145,147,158,159,160,161,162,163,164]. Abbreviations: Agfg1: Arf-GAP domain and FG repeat-containing protein 1; AP3: adaptor complex protein 3; Arap1: Arfgap with RhoGAP domain, ANK repeat and PH domain-containing protein 1; Arfgap: ADP-ribosylation factor GTPase-activating protein; Arfgef: Brefeldin A-inhibited guanine nucleotide-exchange protein; Arfip1, arfaptin 1; Asap: ArfGAP with SH3 domain, ANK repeat and PH domain-containing protein; Bin1: Myc box-dependent-interacting protein 1; Btbd8: AP2-interacting clathrin-endocytosis protein; Gbf1: Golgi brefeldin A-resistant guanine nucleotide exchange factor 1; PLCδ1: 1-phosphatidylinositol 4,5-bisphosphate phosphodiesterase δ1; Smap2: Small Arfgap.

**Figure 7:**
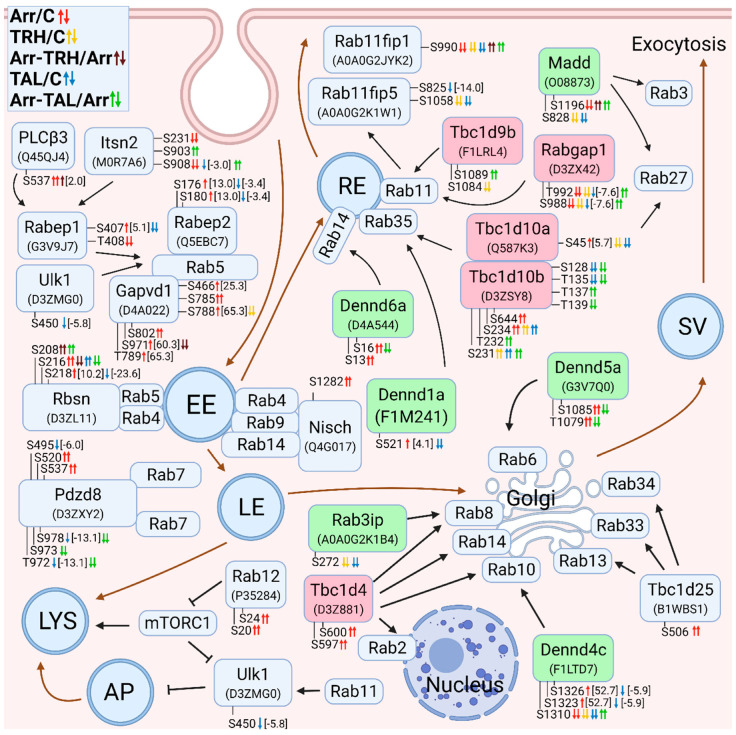
Alterations in phosphorylation of phosphoproteins involved in Rab GTPase-mediated signal transduction. The description of the figure is the same as in Figure 1. Associations and interactions between proteins were ordered according to [130,165,166,167,169,172,173,176,178,179,181,182,183,187,188,189,190,191,192,193]. Abbreviations I: AP: autophagosomes; EEs: early endosomes; LEs: late endosomes; LYS: lysosomes; REs: recycling endosomes; SVs: secretory vesicles. Abbreviations II: Dennd: DENN domain-containing proteins; Gapvd1: GTPase-activating protein and VPS9 domain-containing protein 1; Madd: MAP kinase-activating death domain protein; Pdzd8: PDZ domain-containing protein 8; Rabep: Rab GTPase-binding effector protein; Rabgap: Rab GTPase-activating protein 1; Rab3ip: Rab-3A-interacting protein; Rab11fip: Rab11 family-interacting proteins; Rbsn: rabenosyn-5; Tbc1d: TBC1 domain family members.

**Figure 8:**
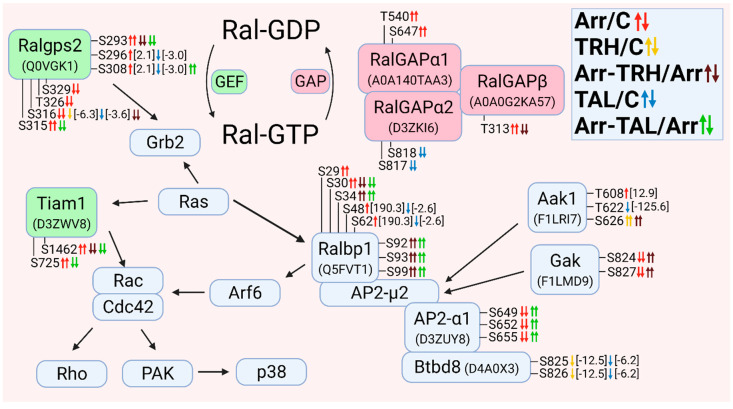
Alterations in phosphorylation of phosphoproteins involved in Ral GTPase-mediated signal transduction. The description of the figure is the same as in Figure 1. Associations and interactions between proteins were ordered according to [71,160,161,194,202,203,204,205]. Abbreviations: Aak1: AP2-associated protein kinase 1; AP2: adaptor protein complex 2; Gak: cyclin-G-associated kinase; Ralbp1: RalA-binding protein 1; RalGAP: Ral GTPase-activating protein; Ralgps2: Ras-specific guanine nucleotide-releasing factor RalGPS2.

**Figure 9:**
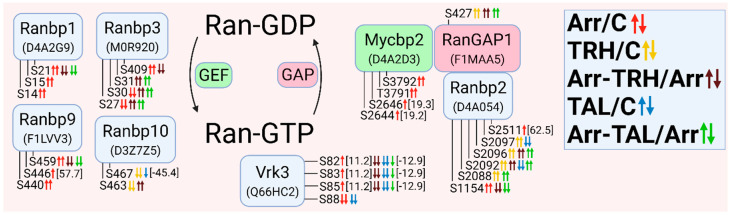
Alterations in phosphorylation of phosphoproteins involved in Ran GTPase-mediated signal transduction. The description of the figure is the same as in Figure 1. Associations and interactions between proteins were ordered according to [207,209,210,211]. Abbreviations: Mycbp2: RCR-type E3 ubiquitin transferase; Ranbp: Ran-binding protein; RanGAP: Ran GTPase-activating protein; Vrk3: serine/threonine-protein vaccinia-related kinase 3.

**Figure 10:**
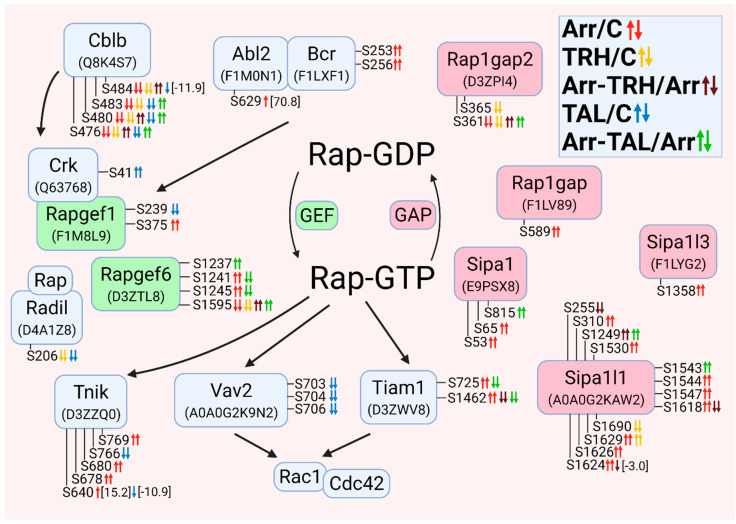
Alterations in phosphorylation of phosphoproteins involved in Rap GTPase-mediated signal transduction. The description of the figure is the same as in Figure 1. Associations and interactions between proteins were ordered according to [212,213,214,215,216,217,218]. Abbreviations: Bcr: breakpoint cluster region protein; Crk: adapter molecule crk; Radil: Ras-associating and dilute domain-containing protein; Rap1gap: Rap1 GTPase-activating protein 1; Rapgef: Rap guanine nucleotide exchange factor; Sipa: signal-induced proliferation-associated proteins; Tnik: TRAF2 and NCK-interacting protein kinase.

**Figure 11:**
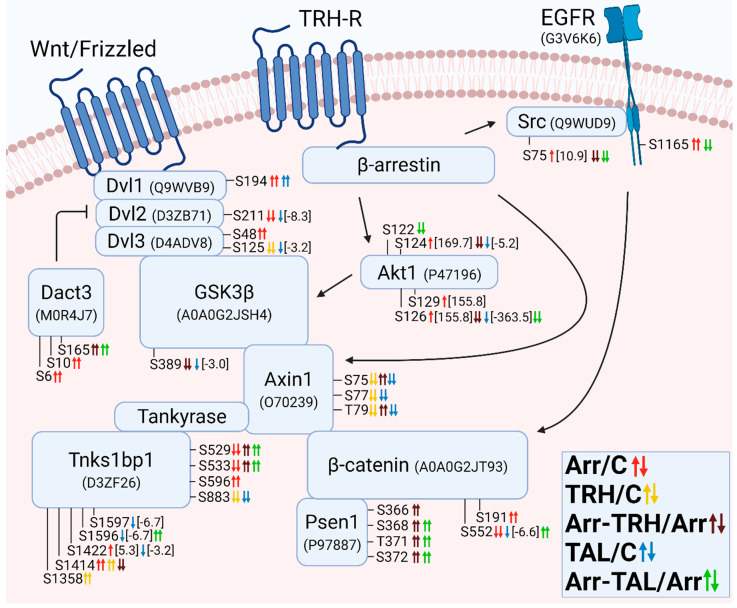
Alterations in phosphorylation of phosphoproteins involved in β-catenin-mediated signal transduction. The description of the figure is the same as in Figure 1. Associations and interactions between proteins were ordered according to [117,219,220,221,222,223,224,225,226]. Abbreviations: Dact3: dishevelled-binding antagonist of β-catenin 3; GSK3β: glycogen synthase kinase-3β; Psen1: presenilin 1; Tnks1bp1: 182 kDa tankyrase-1-binding protein 1.

The altered phosphoproteins were sorted according to their association with classes of small GTPases. The classes of small GTPases most affected by β-arrestin2 knockdown and/or TRH/TAL were Ras and Rho/Rac/Cdc42. For clarity, the changes in phosphorylation and phosphoprotein associations for each class of small GTPases are shown in Figure 1, Figure 2, Figure 3, Figure 4, Figure 5, Figure 6, Figure 7, Figure 8, Figure 9 and Figure 10. The altered phosphoproteins associated with catenin signaling are shown in Figure 11. All these figures were created with BioRender.com (assessed on 1 September 2021).

#### 3.2.1. Alterations in Phosphorylation of Phosphoproteins Involved in Ras GTPase-Mediated Signal Transduction Associated with the PI3K/Akt/mTOR Pathway

The changes in phosphorylation of phosphoproteins involved in Ras GTPase signal transduction are shown in Figure 1 and Figure 2 and listed in detail in Tables S1–S10. Figure 1 shows the changes in phosphorylation when the Ras/Raf and PI3K/Akt/mTOR signaling pathways overlap. It was found that activation of TRH-R by TRH and TAL decreased phosphorylation at Ser94 of B-Raf, and knockdown of β-arrestin2 had no effect on phosphorylation of this phosphosite (Figure 1, Tables S7 and S8). The marked differences between the TRH- and TAL-initiated pathways were evident in the phosphorylation patterns of the upstream and downstream effectors of the mTOR complex. TRH treatment decreased phosphorylation at Ser1389 in the upstream effector TSC2 (Figure 1, Table S2) and at Ser603 in the downstream effector Rps6kc1 (Figure 1, Table S7) and increased phosphorylation at Ser300 in the downstream effector Cdk4 (Figure 1, Table S7), suggesting that phosphorylation at Ser1389 in TSC2 hierarchically precedes phosphorylation at Ser603 in Rps6kc1 and Ser300 in Cdk4. Treatment with TAL only affected the mTOR downstream effector Ulk1 by decreasing phosphorylation at Ser450 (Figure 1, Table S3). Whereas TRH treatment affected the phosphorylation pattern of Rptor, a protein that interacts with mTOR, regulates mTOR kinase activity, and mediates signal transduction through the downstream effector Rps6kc1 [42] by hyperhosphorylating Thr857 and hypophosphorylating Ser859 (Figure 1, Table S9); TAL treatment resulted in only a slight decrease at Ser859 (Figure 1, Table S8). Phosphorylation at Ser859 is known to be mediated by Ulk1 [43]. Rptor is also phosphorylated by 5’-AMP-activated protein kinase (AMPK), resulting in inhibition of mTORC1 [43]. In our study, the catalytic subunit of AMPK, Prkaa1, was hypophosphorylated by TRH at Ser486 and weakly by TAL (Figure 1, Tables S2 and S3). It appears that TRH and TAL act on the activity of the mTORC1 complex in different ways. Whereas TAL appears to act simultaneously on the phosphorylation of Rptor via Ulk1 and AMPK, TRH affects the phosphorylation of Rptor only via AMPK. The phosphosites Ser450 and Ser486 in Ulk1 and AMPK, respectively, are the key sites for regulating the phosphorylation and activity of Rptor. The phosphorylation pattern of Rptor can regulate the activity of the mTORC1 complex, leading to subsequent phosphorylation of various downstream effectors.

Knockdown of β-arrestin2 markedly affected phosphorylation of proteins involved in the Src/EGFR/Ras/PI3K/Akt pathway and downstream effectors of Akt kinase (Figure 1). The Src kinase was hyperphosphorylated at Ser75 (Figure 1, Table S1), which corresponds to Ser75 in human Src with ID P12931. Phosphorylation at this phosphosite is associated only with active Src and is mediated by Cdk5. It promotes ubiquitin-dependent degradation of Src and thus restricts the availability of active Src [44]. This correlates with our finding that knockdown of β-arrestin2 led to dissociation of the β-arrestin2/Src complex and active Src became freely available for phosphorylation at Ser75 and subsequent degradation. Formation and activation of the β-arrestin/Src complex is essential for epidermal growth factor receptor (EGFR) transactivation and downstream activation of Akt [45]. After knockdown of β-arrestin2, EGFR was hyperphosphorylated at Ser1165 (Figure 1, Table S1), whose function is unknown. However, it seems that it may be related to the change in EGFR transactivation. In contrast to hyperphosphorylation at Ser75 in Src after β-arrestin2 knockdown, treatment with TRH or TAL of β-arrestin2-deficient cells induced hypophosphorylation at this phosphosite (Figure 1, Tables S4 and S5), suggesting that TRH and TAL can alter Src activity in β-arrestin2-deficient cells. However, only TAL induced hypophosphorylation at Ser1165 in EGFR in β-arrestin2-deficient cells (Figure 1, Table S5).

Phosphosite Ser181 in PAK4 is located in the region responsible for PAK4 binding to 14-3-3 proteins and its phosphorylation is promoted by PI3K signaling [46]. In the present study, PI3K was not differentially phosphorylated but PAK4 was hyperphosphorylated at Ser181 in β-arrestin2-deficient cells (Figure 1, Table S6). This phosphoresidue corresponds to Ser181 in human Pak4 with ID O96013. Akt1 kinase was hyperphosphorylated after knockdown of β-arrestin2 at Ser124, Ser126, and Ser129 (Figure 1, Table S1). These phosphosites are located in a linker region that is expected to play a role in regulating the conformational states of Akt1 [47]. The phosphosite Ser129 is an important target of casein kinase 2 and its phosphorylation can trigger subsequent phosphorylation at Ser126 of Akt [48]. In our study, both phosphosites in Akt1 were quantitatively hyperphosphorylated at the same level, with a fold change of 155.8 after knockdown of β-arrestin2 (Figure 1, Table S1), which correlates with subsequent hierarchical phosphorylation by casein kinase 2. Casein kinase 2β (CK2β) was hyperphosphorylated after knockdown of β-arrestin2 at Ser154 (Figure 1, Table S6). CK2β, identified via UniProt ID A0A096MJD3, is a 164 amino acid isoform. When comparing the sequence with the human isoform via UniProt ID P67870, the Ser154 phosphosite in CK2β corresponds to the Ser209 phosphosite in the human isoform, whose phosphorylation is associated with mitosis [49]. Phosphorylation at Ser129 in Akt1 (and possibly at Ser126) is responsible for Akt hyperactivation by CK2β [48]. Whereas TRH treatment did not affect the phosphorylation pattern of Akt1 and CK2β, TAL treatment induced hypophosphorylation of Akt1 on Ser124 and Ser126 with concomitant hypophosphorylation of CK2β on Ser158 (Figure 1, Table S3). All altered phosphoresidues in Akt1 correspond to the same phosporesidues at the same positions in human Akt with ID P31749.

The hyperphosphorylation pattern at phosphosites Ser124, Ser126, and Ser129 in Akt1 with its subsequent activation may correlate with changes in phosphorylation of downstream effectors such as MAP3K5, MAP3K9, Sik3, Tsc1/2, or proteins that interact with mTOR (Rptor, Rragc, and Lamtor1) and downstream effectors of the mTOR complex (Cdk4, Rps6kc1) (Figure 1, Tables S1 and S6). In β-arrestin2-deficient cells, both ligands induced hypophosphorylation at Ser26 in Lamtor1 (Figure 1, Tables S4 and S5), a component of the regulator complex that binds mTOR complex 1 to lysosomes [50], as well as at Ser94 in Rragc and Thr857 in Rptor (Figure 1, Tables S4 and S5), suggesting that the same putative kinase or phosphatase acts simultaneously on interacting proteins of the mTORC1 complex in β-arrestin2-deficient cells.

AMPK, which affects downstream and upstream effectors and associated proteins of mTOR, was differentially phosphorylated at its catalytic and regulatory subunits Prkaa1 and Prkab1, respectively. Knockdown of β-arrestin2 resulted in hyperphosphorylation at Thr488 in Prkaa1 and at Ser108 in Prkab1 (Figure 1, Table S6). The second phosphosite Ser108, corresponding to Ser108 in human Prkab1 with ID Q9Y478, is located in the carbohydrate-binding module (CBM) and is an autophosphorylation site or can be phosphorylated by Ulk1 [51,52]. Its phosphorylation stabilizes the interaction of the CBM with the kinase domain and increases AMPK activity and affinity for binding of allosteric drugs and metabolites [51,52,53], suggesting that knockdown of β-arrestin2 causes an increase in AMPK activity and phosphorylation of AMPK substrates, e.g., TSC1, TSC2, Thr857 in Rptor. In β-arrestin2-deficient cells, TRH treatment induced hyperphosphorylation at Thr526, whereas TAL treatment caused hyperphosphorylation at Thr526 and Ser527 and hypophosphorylation at Thr488 in the Prkaa1 catalytic subunit of AMPK (Figure 1, Tables S9 and S10). These phosphosites are located in the β-ID domain of the C-terminal ST loop, which has a regulatory function for AMPK kinase activity [53]. Phosphorylation at Thr488 is mediated by Ulk1 [43]. All three phosphosites correspond to the same phosphoresidues at same positions in human Prkaa1 with ID Q13131.

For the phosphoprotein Lats1, the change at Ser1111 occurred in all pairwise comparisons. This protein was hypophosphorylated in β-arrestin2-deficient cells, TRH-treated cells, or TAL-treated cells. Subsequent TRH or TAL treatments of β-arrestin2-deficient cells induced hyperphosphorylation at this phosphosite (Figure 1, Tables S6–S10). Although Stk3 (Mst2) kinase, whose phosphosite Ser316 was hyperphosphorylated in β-arrestin2-deficient cells and hypophosphorylated in TAL-treated cells (Figure 1, Tables S6 and S8), phosphorylates some phosphosites in Lats1; phosphosite Ser1111 was excluded as a site regulated by Stk3 [54]. Its phosphorylation can be mediated by AMPK or Map4k kinases, which are thought to regulate Lats1 activity [55,56]. Both AMPK and Map4k kinases were found to be differentially phosphorylated (Figure 1). These data suggest that both AMPK and Map4k kinases may mediate phosphorylation of Lats1. The second affected phosphosite in Lats1, Ser464, was hyperphosphorylated in β-arrestin2-deficient cells (Figure 1, Table S6). Lats1 is phosphorylated by the kinase Nuak1 but not by AMPK [57]. This phosphorylation reduces the stability of Lats1 [57], suggesting that knockdown of β-arrestin2 can lead to a reduction in Lats1 levels.

#### 3.2.2. Alterations in Phosphorylation of Phosphoproteins Involved in Ras GTPase-Mediated Signal Transduction Associated with the Grb2/Sos/Ras/Raf/MEK/ERK Pathway

Figure 2 shows the changes in phosphorylation of members of the Grb2/Sos/Ras/Raf/MEK/ERK signaling pathways. Although the adapter protein Grb2, which mediates signal transduction from the activated membrane receptor to Ras, was not directly affected by the phosphorylation changes, its upstream and downstream interacting partners Gab1 and Sos1, respectively, were differentially phosphorylated. After TRH treatment, the docking protein Gab1 was hypophosphorylated at Ser438 (Figure 2, Table S7), which is located upstream near a Met-binding domain (MBD) [73] and corresponds to Ser439 in human Gab1 with ID Q13480. In contrast, the second interaction partner, Sos1, was affected to a greater extent, as β-arrestin2 knockdown caused differential phosphorylation of seven phosphosites (Ser1078, Ser1082, Thr1249, Ser1251, Thr1255, Ser1318, Ser1319) (Figure 2, Table S1) located in the C-terminal G domain containing the Grb2 binding site [74]. While Ser1078 and Ser1082 correspond to the same phosphoresidues at the same positions in human Sos1 with ID Q07889, Thr1249, Ser1251, Thr1255, Ser1318, and Ser1319 correspond to Thr1263, Ser1265, Thr1269, Ser1332, and Ser1333, respectively. Docking of this region in Sos1 to Grb2, which binds to the activated receptors, mediates the primary anchoring of Sos1 to the plasma membrane [75] suggesting that the presence of β-arrestin2 is essential for the formation and/or function of the Grb2/Sos1 complex. Whereas no change in phosphorylation of Sos1 was detected after TRH treatment, treatment with TAL resulted in a slight hypophosphorylation at Ser1078 and Ser1082 (Figure 2, Table S3). In contrast to this difference, phosphorylation patterns were very similar after TRH or TAL treatments of β-arrestin2-deficient cells. Both ligands caused hyperphosphorylation at Ser1072 and at Ser1082 and hypophosphorylation at Thr1255; only Ser1319 was hyperphosphorylated after treatment with TAL (Figure 2, Tables S4 and S5). It is evident that the presence of β-arrestin2 diversifies the signaling triggered by TRH receptors activated by TRH or TAL and that its absence abolishes the differences.

The MEK1/2 and ERK1/2 kinases were not directly affected in their phosphorylation patterns, but their interacting partners and downstream effectors were differentially phosphorylated. Two pseudokinases, the kinase suppressors of Ras 1 and 2 (KSR1 and KSR2), serve as scaffolds linking Raf isoforms and their substrates [76]. The KSR2 protein was hyperphosphorylated at Thr272 and Thr276 in β-arrestin2-deficient cells and hypophosphorylated at Thr276 in TAL-treated cells and at Thr272 in β-arrestin2-deficient cells treated with TAL (Figure 2, Tables S1, S3 and S5). These phosphosites are located outside the binding sites with Raf, MEK, and ERK [77]. The interacting protein of KSR1, connector enhancer of kinase suppressor of Ras1 (Cnksr1), was hyperphosphorylated in β-arrestin2-deficient cells at Thr284 and Ser288 (Figure 2, Table S1). In contrast, phosphosite Thr284 was hypophosphorylated in β-arrestin2-deficient cells treated with TAL (Figure 2, Table S5). These phosphosites in Cnksr1 are located in disordered regions outside the domain structures [78].

The activated ERK phosphorylates many substrates, including Rps6ka3 (Rsk2) [79], Rreb1 [80], and Cdk4 [64]. The data suggest that the transcription factor Rreb1, which is differentially phosphorylated in all five experimental groups, plays a key role in signaling triggered by TRH or TAL, and that β-arrestin2 is a mediator of this signaling, possibly affecting Rreb1 function.

Doublecortin-like kinase protein 1 (Dclk1) was found to be differentially phosphorylated on ten phosphosites with different phosphorylation patterns under different experimental conditions (Figure 2, Tables S1–S5). All phosphosites corresponding to the same residues at the same positions in human Dclk1 with ID O15075 are located in a Pro-Ser-rich linker targeted by calpain for proteolytic cleavage and involved in the regulation of Dclk1 kinase activity and tubulin polymerization [81,82,83]. Interestingly, knockdown of Dclk1 resulted in downregulation of KRas and Rreb1 [84], suggesting that if phosphorylation in the Pro-Ser-rich linker is related to Dclk1 proteolysis, expression of KRas and Rreb1 can also be affected. The Dclk1 and Rreb1 proteins represent other key points of differential effects of TRH and TAL on signaling pathways. Downregulation of Dclk1 resulted in increased expression of the miR-143/145 cluster and downregulation of its downstream targets KRas and Rreb1 [84]. The transcription factor Rreb1 is activated by the MAPK pathway and negatively regulates the miR-143/145 promoter by binding to the Ras responsive element (RRE), thereby increasing the expression of KRas and Rreb1 [85].

#### 3.2.3. Alterations in Phosphorylation of Phosphoproteins Involved in Rho GTPase-Mediated Signal Transduction

The second class of small GTPases affected by β-arrestin2 knockdown and/or TRH/TAL treatment is a Rho/Rac/Cdc42 class. The changes in phosphorylation occurred in guanine nucleotide exchange factors (GEFs), GTPase-activating proteins (GAPs), and downstream effectors. For clarity, proteins with changes in phosphorylation were sorted according to their association with Rho GTPase (Figure 3), Rac GTPase (Figure 4), and Cdc42 GTPase (Figure 5). The proteins with GEF or GAP activities are highlighted in green and red, respectively. In many cases, phosphoproteins are phosphorylated at phosphosites that are close to each other, forming clusters of phosphosites that are usually phosphorylated with the same phosphorylation patterns.

When proteins with GEF or GAP activities were compared, the larger set of GEFs that activate Rho GTPases were found to be differentially phosphorylated. β-Arrestin2 knockdown resulted in hyperphosphorylation of GEFs and GAPs, whereas treatment of wild-type cells with TRH and TAL resulted in hypophosphorylation of GEFs and GAPs. Treatment with TAL had a more pronounced effect on differential phosphorylation of GEFs than treatment with TRH (Figure 3).

Of the phosphosites with changes in phosphorylation in GEFs, only one was in functional domains. Phosphosite Ser48 in dishevelled segment polarity protein 3 (Dvl3) was hyperphosphorylated in β-arrestin2-deficient cells (Figure 3, Table S1) and was located in the DIX domain in the N terminus that binds to the DIX domain in Dvl1 [90]. It corresponds to Ser48 in human Dvl3 with ID Q92997. Other phosphosites were located in disordered regions by determining their positions using the UniProt database.

All GAPs were also differentially phosphorylated at phosphosites located in disordered regions by determining their position using the UniProt database. The two GAPs, Myo9b and Arhgap35, were hypophosphorylated at several phosphosites after TRH or TAL treatments (Figure 3, Tables S2 and S3), but knockdown of β-arrestin2 abolished the decreased phosphorylation induced by TRH or TAL, except for hyperphosphorylation at Ser1982 in Myo9b after TAL treatment (Figure 3, Table S5). In addition to GEFs and GAPs, several Rho-GTP downstream effectors and their associated proteins were differentially phosphorylated (Figure 3).

Whereas TRH and TAL treatments caused hypophosphorylation of Rho downstream effectors in wild-type cells, they affected phosphorylation of effectors in both directions in β-arrestin2-deficient cells. The phosphosite Ser1124 in Rock2 was hyperphosphorylated in β-arrestin2-deficient cells and in wild-type cells treated with TRH or TAL, but hypophosphorylated in β-arrestin2-deficient cells treated with TRH or TAL (Figure 3, Tables S1–S5). Three downstream Map3 kinases (Map3k5, Zak, Map3k7) were differentially phosphorylated (Figure 3, Tables S6, S8 and S10). In β-arrestin2-deficient cells, TRH and TAL induced changes in phosphorylation of Ser/Thr protein kinase 1 (Pkn1) and protein kinase Cε (Figure 3, Tables S9 and S10). According to the UniProt database, the differentially phosphorylated phosphosite Ser920 in Pkn1 is located in the C-terminal domain of the AGC kinase and corresponds to Ser916 in human Pkn1 with ID Q16512. The most affected protein kinase is protein kinase D1 (PKD1), which was differentially phosphorylated in three phosphorylation clusters.

#### 3.2.4. Alterations in Phosphorylation of Phosphoproteins Involved in Rac GTPase-Mediated Signal Transduction

Overall, treatment with TRH and TAL induced hypophosphorylation of GEFs, GAPs, and downstream effectors of Rac GTPases (Figure 4). The number of GEFs and GAPs with changes in phosphorylation pattern was similar (Figure 4). Several GEFs that affect the activity of Rho GTPases (e.g., dishevelled proteins, Prex2, Camk2β, Mcf2l, and Vav2) are also known to control the GTP/GDP cycle of Rac GTPases [94,95,101,110]. Treatment with TAL strongly affected the phosphorylation pattern of Vav2, Dvl1, Dvl2, and CK2β (Figure 4, Tables S3 and S8). CK2β is a subunit of casein kinase 2 that phosphorylates Dvl proteins, resulting in a change in the signaling function of Dvl and displacing it from the Rac1 pathway [118]. CK2β was hyperphosphorylated and hypophosphorylated at Ser154 in β-arrestin2-deficient cells and after treatment with TAL, whereas phosphosite Ser158 was hypophosphorylated after treatment with TAL (Figure 4, Tables S6, S8 and S10). Comparison of the sequences in the UniProt database revealed that this phosphosite Ser158 in rat CK2β with protein ID A0A096MJD3 corresponds to Ser209 in the C-terminal end of human CK2β with ID P67870. Phosphorylation at Ser209 in human CK2β is mediated by p34cdc2 (Cdk1) [49].

The most important Rac GEF appears to be Dock7, which was differentially phosphorylated in all five pairwise experimental groups (Figure 4, Tables S1–S5), resulting in different phosphorylation patterns. The GAP proteins were frequently affected by both TRH and TAL treatments (Figure 4, Tables S1–S5). The Ser/Thr protein kinase Wnk2 was differentially phosphorylated in the first phosphorylation cluster located in the N-terminus [119] in wild-type cells treated with TAL and in β-arrestin2-deficient cells treated with TRH (Figure 4, Tables S3 and S4). However, treatment with TAL of β-arrestin2-deficient cells induced hypophosphorylation of Wnk2 (Figure 4, Table S5) in the region between the conserved region 3 and the WNK homology region III [119]. Wild-type cells treated with TRH exhibited changes in phosphorylation in both clusters (Figure 4, Table S2).

Several upstream and downstream Rac GTPase effectors were found to be differentially phosphorylated (Figure 4). The Erbb2 receptor was hypophosphorylated after treatment with TAL (Figure 4, Table S3). Erbb2-mediated signaling involves the PI3K-Rac-Pak pathway and stabilizes an actin cytoskeletal complex containing PI3K, Vav2, Rac1, and Pak1 [120]. In our study, treatment with TAL affected the phosphorylation patterns of Erbb2, Vav2, Pak1, and Pak2 (Figure 4, Tables S3 and S8), suggesting that Pak2 may be involved in this pathway. In contrast to Pak2, which was affected only by TAL, TRH treatment caused hypophosphorylation on Ser174, Ser219, and Ser228 in wild-type Pak1 but TAL only on Ser219 (Figure 4, Tables S7 and S8). This protein was also hypophosphorylated after TRH or TAL treatments of β-arrestin2-deficient cells, but with different phosphorylation patterns (Figure 4, Tables S9 and S10). Rac1 and PAK1 formed a complex with Arhgef7 [121], which is also a regulator of the Hippo pathway involving the Lats1 protein [122]. The downstream effector of Pak2, Map3k1, was hyperphosphorylated at Ser513 after treatment of β-arrestin2-deficient cells with TAL (Figure 4, Table S10).

Another interacting partner of PI3K with the stimulating effect on Rac activity is the kinase Ptk2β which was found to be hypophosphorylated at Ser389, Ser392, Ser394, Ser396, and Ser399 in wild-type cells after treatment with TRH or TAL and in β-arrestin2-deficient cells after treatment with TAL (Figure 4, Tables S2, S3 and S5). Two SRSF protein kinases, Srpk1 and Srpk2, involved in Rac1 alternative splicing [123] were found to be differentially phosphorylated (Figure 4).

#### 3.2.5. Alterations in Phosphorylation of Phosphoproteins Involved in Cdc42 GTPase-Mediated Signal Transduction

Most Cdc42-GEFs were differentially phosphorylated compared with Cdc42-GAPs (Figure 5). Some Cdc42-GEFs also activate Rac (Dock6, Dock7, Tiam1, Marcks), Rho (Itsn1), or both Rac and Cdc42 (Dvl1-Dvl3, Prex2, Mcfl2) (Figure 3, Figure 4 and Figure 5). The Cdc42-GEF Farp2 is also a GAP for Rac (Figure 4 and Figure 5). The long variant of Arhgef7 (βPIX, UniProt ID A0A0G2QC21) with 862 amino acids and a calponin homology (CH) domain at the N terminus was hyperphosphorylated at Thr159 in β-arrestin2-deficient cells treated with TRH or TAL and at Ser497 in β-arrestin2-deficient cells (Figure 5, Tables S1, S4 and S5). The phosphosite Thr159 is located between the domains CH and SH3 (according to the UniProt database). Comparison of the evolutionarily conserved sequence ASPRM^340^SGFIYQ [146] with the sequence position of differentially phosphorylated Ser497 revealed that phosphosite Ser497 in the long variant of Arhgef7 with 862 amino acids corresponds to phosphosite Ser340 in the conserved sequence ASPRM340SGFIYQ. This phosphosite, corresponding to Ser518 in human Arhgef7 with ID Q14155, is located in the domain PH, which interacts with phosphatidylinositol lipid in membranes, PKC and Cdc42 and is involved in the regulation of GEF activity of Sos [146]. Phosphorylation of Ser340 is indirectly mediated by PKC and involved in the regulation of dopamine release [146].

Arhgef7 also interacts with several binding partners, such as Git1, Scribble, Pak kinases, Rac1, and Cblb [121,147]. Git1 was differentially phosphorylated in two phosphorylation clusters (Figure 5). The first cluster (Ser376, Ser379, Thr383) was affected in all five pairwise experimental groups. Whereas knockdown of β-arrestin2 or treatment with TAL induced hyperphosphorylation, treatment of wild-type and β-arrestin2-deficient cells with TRH caused hypophosphorylation (Figure 5, Tables S1–S5). The second cluster (Ser583, Ser587, Tyr589) was hyperphosphorylated after β-arrestin2 knockdown (Figure 5, Table S1). These three phosphoresidues correspond to Ser592, Ser596, and Tyr598 in human Git1 with ID Q9Y2X7. Comparison of the conserved sequences in Git1 [148], with the sequence location of the detected phosphosites with changes revealed that these phosphosites are located in the SLD domain that Git1 localizes to synapses [148]. Comparison of the two amino acid sequences revealed that the phosphosites Ser376, Ser379, Thr383 and Ser583, Ser587, Tyr589 found in Git1 (Figure 5) match Ser394, Ser397, Thr401 and Ser601, Ser605, Tyr607, respectively, in Git1 described by Webb et al. [148]. The putative kinases of Ser601 or Tyr607 (Ser583 and Tyr589 in the second cluster in our study) were determined to be PKC, PKA, GSK3, and EGFR, respectively [148]. In our study, EGFR, the regulatory subunits of PKA (Prkar1a, Prkar2a; Figure 2), and protein kinase Cδ (PKCδ, Figure 3) were changed in β-arrestin2-deficient cells (Tables S1 and S6). Scrib was differentially phosphorylated in three phosphorylation clusters (Figure 5). The first and second clusters were affected in β-arrestin2-deficient cells with or without ligand treatment (Figure 5, Tables S1, S4 and S5). The phosphosite Ser1483 in the third cluster was hypophosphorylated after treatment with TAL (Figure 5, Table S3). All phosphosites are located outside the PDZ domain (protein ID A0A0G2QC21; UniProt database) through which Scrib binds to Arhgef7 [145]. The Scrib-Arhgef7-Git1 complex is involved in vesicle transport in neurons and its binding partners are Pak kinases activated by Cdc42 or Rac [145].

Cblb belongs to ubiquitin E3 ligases of the Cbl family, which bind to Arhgef7 after Cdc42 activation, leading to an increase in GEF activity of Arhgef7 in a positive feedback loop [147]. Cblb was hypophosphorylated at Ser476, Ser480, Ser483, and Ser484 after TRH or TAL treatment in wild-type cells, whereas these phosphosites were hyperphosphorylated in different phosphorylation patterns after TRH or TAL treatment in β-arrestin2-deficient cells (Figure 5, Tables S2–S5). Ser484 is phosphorylated by an unknown kinase and its phosphorylation creates a GSK3 consensus phosphorylation site on Ser480 [149]. Ser480 was found to be phosphorylated by GSK3, creating another GSK3 consensus phosphorylation site on Ser476, which in turn is phosphorylated by GSK3. Phosphorylation of both sites is required for maintenance of Cblb stability [149]. All four phosphoresidues correspond the same residues at the same positions in human Cblb with ID Q13191.

Only five Cdc42-GAPs were differentially phosphorylated (Figure 5, Tables S1–S5). All Cdc42-GAPs inactivated not only Cdc42 but also Rac and/or Rho GTPases (Figure 3, Figure 4 and Figure 5). The affected Cdc42 downstream effectors included Pak kinases (Pak1, Pak2, and Pak6), Map3k7, Cdc42-binding protein kinases (Cdc42bpa, Cdc42bpb), and Cdc42 effector protein (Cdc42ep4) (Figure 5, Tables S1–S5).

#### 3.2.6. Alterations in Phosphorylation of Phosphoproteins Involved in Arf GTPase-Mediated Signal Transduction

As mentioned above, Arhgef7 is a binding partner for Scrib and Git1 and a scaffold for members of the Hippo pathway (Lats1, Stk3). Git1/2 are proteins with GAP activity for Arf GTPases [147]. The Git1-Arhgef7 complex regulates the activity of Arf6 to control phospholipase C (PLCδ), which promotes vesicle fusion with the membrane [147]. In our study, PLCδ1 was differentially phosphorylated in a phosphorylation cluster (Figure 6). While Ser454 and Ser460 were hyperphosphorylated in wild-type cells after ligand treatment, knockdown of β-arrestin2 abolished these increased phosphorylations and caused hyperphosphorylation at Thr457 only after treatment with TAL (Figure 6, Tables S2, S3 and S5).

Five proteins with GEF activity for Arf proteins were found to have differential phosphorylation. Gbf1, an Arf1 GEF, was hyperphosphorylated after β-arrestin2 knockdown and after TRH treatment of wild-type cells and hypophosphorylated in wild-type and in β-arrestin2-deficient cells treated with TAL (Figure 6, Tables S1–S3 and S5). Arf1 interacts with protein kinase D2 [158]. In our study, PKD2 was hyperphosphorylated after β-arrestin2 knockdown (Figure 6, Table S6). Arf1 activates PI3K with subsequent phosphorylation of Akt [159]. Arf1 interacts with AP3 complex via its β-subunit [160]. Btbd8 protein (APache, Kiaa1107) interacts with the δ subunit of AP3 [161]. Both AP3-β1 and Btbd8 were found to be hypophosphorylated in wild-type cells after treatment with TRH or TAL (Figure 6, Table S11).

Three Arfgef proteins were found to be differentially phosphorylated, Arfgef1 and Arfgef2 in three phosphorylation clusters. More Arf-GAPs were differentially phosphorylated than Arf-GEFs (Figure 6). Git2 was differentially phosphorylated in two phosphorylation clusters. In the first cluster (Tyr392, Ser394, and Ser397) it was differentially phosphorylated in β-arrestin2-deficient cells, hypophosphorylated after treatment with TRH or TAL, and hyperphosphorylated in β-arrestin2-deficient cells treated with TAL (Figure 6, Tables S1–S3 and S5). In the second cluster (Ser415, Ser418, and Ser421) it was hyperphosphorylated only in β-arrestin2-deficient cells treated with TAL (Figure 6, Table S5). By sequence comparison, Ser394 and Ser397 in Git2 correspond to Ser376 and Ser379 in Git1, respectively (protein IDs A0A0G2K527 and Q66H91; UniProt Database) [148]. Tyr392 is phosphorylated by Src/FAK kinases and is required for binding to paxillin at focal adhesions and binding to the SH2-SH3 adaptor proteins Nck1 and Nck2 [147]. Phosphorylated Ser415 is a binding site for 14-3-3 proteins [147]. All six altered phosphoresidues in Git2 correspond to the same residues at the same positions in human Git2 with ID Q14161.

#### 3.2.7. Alterations in Phosphorylation of Phosphoproteins Involved in Rab GTPase-Mediated Signal Transduction

Rab GTPases are important regulators of membrane transport and are involved in membrane fission, transport, tethering, docking, and fusion [165]. In our study, many phosphoproteins involved in membrane trafficking by Rab GTPases were differentially phosphorylated (Figure 7). The process of membrane trafficking begins with early endocytosis, which is regulated by Rab5 GTPases localized in early endosomes (EEs) [166]. Phosphorylation patterns of Rab5-associated proteins (Rabep1, Rabep2, Itsn2, Gapvd1, and PLCβ3) were markedly affected in β-arrestin2-deficient cells (Figure 7, Tables S1 and S6). Rabep1, Rabep2, Itsn2, and Ulk1 were hypophosphorylated in wild-type cells after treatment with TAL (Figure 7, Tables S3 and S8), and treatment with TRH had an effect only on Gapvd1 phosphorylation in wild-type and β-arrestin2-deficient cells (Figure 7, Tables S2 and S4). Rabep1, Rabep2, Gapvd1, and Ulk1 regulate Rab5 activity or localize and recruit Rab5 to different intracellular compartments and are thus involved in the formation of early endosomes [166,167,168,169]. Intersectin-2 (Itsn2) interacts with Rabep1 and stimulates Rabep1 degradation to regulate endocytosis and endosome transport [166]. Rabep1 was differentially phosphorylated in the Rabaptin domain (protein ID G3V9J7; UniProt Database). The phosphoresidues Ser407 and Thr408 correspond to Ser407 and Thr408 in human Rabep1 with ID Q15276. Rabep1 was shown to be a downstream effector of phospholipase C β3 (PLCβ3). Activation of the Gαq protein via the protease-activated receptor induces phosphorylation of PLCβ3 at Ser537, which is located in the X-Y linker that is close to the C-terminal coiled-coil domain of PLCβ3 and the N-terminus of the Gαq protein in the crystal structure of the complex [170,171] and corresponds to the same phosphoresidue at the same position in human PLCβ3 with ID Q01970. At the same time, Rabep1 was found to be hypophosphorylated at Ser407, in parallel with hyperphosphorylation of protein kinase D1 at Ser745 and Ser910 [171]. In our study, these two phosphosites in Rabep1 were differentially phosphorylated after knockdown of β-arrestin2, but Ser407 was slightly hyperphosphorylated (Figure 7) and protein kinase D1 was not differentially phosphorylated at Ser745 and Ser910 (Figure 3), suggesting that activation of Gαq protein via TRH-R and subsequent PLCβ3 phosphorylation at Ser537 involves the other downstream mechanism of Rabep1 phosphorylation. The data suggest that TAL and, to a lesser extent, TRH regulate Rab5 activity and early endosome formation and that this effect is abolished by β-arrestin2 deficiency.

Rabenosyn (Rbsn) serves as a molecular link between Rab5 and a specific phosphatidylinositol 3-kinase called hVPS45 and is recruited to early endosomes in a PI3K-dependent manner [172]. It also binds to Rab4 [173]. In our study, it was differentially phosphorylated in wild-type cells after treatment with TAL and after knockdown of β-arrestin2 without or with subsequent ligand treatment (Figure 7, Tables S1 and S3–S5). The differentially phosphorylated phosphosites Ser208, Ser216, and Ser218 are located in the FYVE finger domain [173], which is essential for the rabenosyn recruitment to PI3P-enriched Rab5 endosomes [172]. They correspond to Ser209, Ser217, and Ser219 in human Rabenosyn with ID Q9H1K0.

The other interacting Rab protein, nischarin (Nisch), interacts with Rab4, Rab9, and Rab14 localized to endosomes and can be localized to endosomes by binding PI3P through its PX domain [130]. In our present study, Nisch was hyperphosphorylated at Ser1282 after knockdown of β-arrestin2 (Figure 7, Table S1). This phosphosite is located in the C-terminal domain (CTD) responsible for interaction with Rab14, Rab4, Rab9, and GTP-Rac1 [130] and corresponds to Ser1284 in human Nischarin with ID Q9Y2I1.

The early endosomes can be recycled to the plasma membrane via recycling endosomes [174]. Dennd6a (FAM116A) has GEF activity toward Rab14 and, to a lesser extent, Rab35, and is required for recruitment of Rab14 to recycling endosomes [175]. In our study, Dennd6a was hyperphosphorylated or hypophosphorylated at N-terminal phosphosites after β-arrestin2 knockdown and after treatment of β-arrestin2-deficient cells with TAL (Figure 7, Tables S1 and S5). Rab11 and Rab35 are the essential Rabs involved in endosome recycling to the plasma membrane [165]. Three proteins associated with Rab35 were found to be differentially phosphorylated. Dennd1a, which was hyperphosphorylated and hypophosphorylated in the disordered region at Ser521 after β-arrestin2 knockdown and in wild-type cells treated with TAL (Figure 7, Table S1 and S3), is a Rab35-GEF that recruits Rab35 to clathrin-coated pits in early endosomes and activates Rab35 primarily to enable the formation of membrane carriers that mediate recycling of selective cargo [176]. Tbc1d10a and Tbc1d10b are Rab35 GAPs that control the formation of recycling carriers from endosomes [176]. While Tbc1d10a was differentially phosphorylated only at Ser45 in the N-terminal disordered region after treatment with TRH or TAL and by β-arrestin2 knockdown (Figure 7, Tables S1–S3), Tbc1d10b was differentially phosphorylated at eight phosphosites, with TAL treatment of wild-type or β-arrestin2-deficient cells having the greatest effect (Figure 7, Tables S1–S3 and S5).

Rabgap1 and Tbc1d9b have GAP activity to Rab11 [177,178]. Phosphosites Ser988 and Thr992 in Rabgap1 were hypophosphorylated after β-arrestin2 knockdown and after treatment with TRH or TAL and hyperphosphorylated in β-arrestin2-deficient cells treated with TAL (Figure 7, Tables S1–S3 and S5). Tbc1d9b was hypophosphorylated at Ser1084 after treatment with TRH and hyperphosphorylated at Ser1089 in β-arrestin2-deficient cells after treatment with TAL (Figure 7, Tables S2 and S5), suggesting that TRH treatment and β-arrestin2 knockdown may affect GTP hydrolysis of Rab11. Rab11fip proteins serve as effectors of Rab11 and regulate trafficking by recycling endosomes [179]. In wild-type cells, both Rab11fip1 and Rab11fip5 were hypophosphorylated after treatment with TRH and TAL; however, knockdown of β-arrestin2 had an effect only on Rab11fip1 phosphorylation (Figure 7, Tables S1–S5). Phosphoresidues Thr972, Ser973, and Ser978 correspond to Thr974, Ser975, and Ser980, respectively, in human Pdzd8 with ID Q8NEN9.

Rab7 regulates cargo transport from early endosomes to late endosomes and subsequently for degradation in lysosomes [180]. An interacting Rab7 protein, PDZD8, was found to be differentially phosphorylated in two phosphorylation clusters (Figure 7). The phosphosites in the second cluster are located in the area between regions C1 and CC, which is responsible for the interaction of GTP-Rab7 with late endosomes [181].

Autophagy is responsible for the degradation of intracellular components by transporting them to lysosomes to maintain cellular homeostasis [180]. Rab12 is an autophagic regulator that controls the degradation of an amino acid transporter [182]. In our study, Rab12 was hyperphosphorylated at Ser20 and Ser24 in its N-terminal disordered region (Figure 7, Table S1). One of the major sensors of intracellular amino acid levels is mTORC1 [183], whose components and associated proteins were differentially phosphorylated (Figure 1). mTORC1 activity is regulated by Rab12 [182]. Ulk1, which is regulated by mTORC1, is involved in the initiation of autophagosome formation and autophagosome-lysosome fusion [184,185]. However, it is not clear whether phosphorylation of Ulk1 on Ser450 and of Rab12 on Ser20 and Ser24 has effects on autophagy processes.

Several Rab proteins are associated with the Golgi network [165,183]. Dennd5a was hyperphosphorylated after β-arrestin2 knockdown at Thr1079 and Ser1085 but hypophosphorylated in β-arrestin2-defficient cells after treatment with TAL (Figure 7, Tables S1 and S5). Dennd5a binds to Rab11 and Rab6 [186]. Both altered phosphosites are located between the PLAT and RUN2 domains (protein ID G3V7Q0; UniProt Database), outside the uDENN (upstream DENN) module interacting with Rab11 [186], suggesting that the altered phosphorylation pattern may be associated with Rab6 in the Golgi rather than Rab11. They correspond to the same residues at the same positions in human Dennd5a with ID Q6IQ26. A vesicle transport pathway from the trans-Golgi network to the plasma membrane is mediated by secretory vesicles via Rab3 and Rab27, whose activities are influenced by Madd [180,187].

#### 3.2.8. Alterations in Phosphorylation of Phosphoproteins Involved in Ral GTPase-Mediated Signal Transduction

Although only a few proteins associated with Ral GTPases were found to be differentially phosphorylated, some alterations might be associated with a variety of signaling pathways and biological processes. Ralgps2, a GEF protein for RalA [194], was differentially phosphorylated on several phosphosites in sequence from Ser293 to Ser329 in all five pairwise comparisons, with greater effect of TAL than TRH in both cells (Figure 8, Tables S1–S5). The phosphosite Ser329, corresponding to Ser329 in human Ralgps2 with ID Q86X27, is located in the sequence ^321^LLPXTPP^329^SP with PXXP motif (protein ID Q0VGK1; UniProt Database) which is required for Grb2 binding and regulation in Ralgps1 [195], suggesting Ralgps2 might also bind Grb2 and this interaction might be regulated by phosphorylation at Ser329.

Ral-binding protein 1 (Ralbp1/Rlip76) was found to be differentially phosphorylated in its N-terminal domain in three phosphorylation clusters (Figure 8, Tables S1 and S3–S5). All phosphoresidues correspond to the same residues at the same positions in human Ralbp1 with ID Q15311. In the first cluster, Ser29 and Ser30 were hyperphosphorylated after knockdown of β-arrestin2 (Figure 8, Table S1). After treatment of β-arrestin2-deficient cells with TRH and TAL, phosphosites Ser30 and Ser34 were hypophosphorylated and hyperphosphorylated, respectively (Figure 8, Tables S4 and S5). Phosphosites Ser29 and Ser30 are responsible for binding ARNO, the ArfGEF that activates Arf6 [196]. In the second cluster, Ser48 and Ser62 were hyperphosphorylated after knockdown of β-arrestin2 and hypophosphorylated after treatment with TAL (Figure 8, Tables S1 and S3). It was shown that phosphorylation at Ser48 was not detected in peptides lacking Ser62, suggesting that phosphorylation of Ser62 is mandatory for Ser48 to be phosphorylated or that Ser62 is constitutively phosphorylated [197]. In the third cluster, Ser92, Ser93, and Ser99 were hyperphosphorylated in β-arrestin2-deficient cells after treatment with TRH or TAL (Figure 8, Tables S4 and S5). Phosphorylation of Ser99 was not detected in peptides that were not phosphorylated at Ser92 and Ser93, which were always modified simultaneously [197]. Computationally derived putative kinases include MAPK, CK2, and cyclin-dependent kinase [197], suggesting that changes in Ralbp1 phosphorylation may be associated with phosphorylation changes in CK2 and MAPK signaling pathways (Figure 2, Figure 3 and Figure 4). The N-terminal sequence of Ralbp1 (residues 1–190) is involved in an interaction with Ras GTPase that leads to ARNO and subsequent activation of Arf6 [196]. Arf6 can regulate Rac1 activity by interacting with kalirin [198]. Arf6-dependent membrane trafficking is associated with the dynamics of Cdc42-positive vesicles and controls the localization of Cdc42 and Arhgef7 [199]. This sequence of Ralbp1 also interacts with the interdomain linker of the μ2-subunit of the clathrin adaptor complex, AP2, resulting in receptor-mediated endocytosis of EGFR [196]. The α1-subunit of AP2, which interacts with the μ2-subunit and differentially phosphorylates Btbd8, was hypophosphorylated after knockdown of β-arrestin2 and hyperphosphorylated in β-arrestin2-deficient cells treated with TAL (Figure 8, Table S11).

The μ2-subunit of AP2 can be phosphorylated by the adaptor-associated kinase AAK1, which is colocalized with many components of the endocytic machinery such as clathrin, AP2, and dynamin 1 [200]. In our study, AAK1 was hyperphosphorylated after β-arrestin2 knockdown at Thr608 and at Ser626 after treatment of wild-type and β-arrestin2-deficient cells with TRH (Figure 8, Tables S6, S7, and S9). After treatment with TAL, it was hypophosphorylated at Ser622 (Figure 8, Table S8). Comparing this AAK1 isoform with a sequence of 963 amino acids (protein ID F1LRI7; UniProt Database) with the AAK1 isoform with 962 amino acids (protein ID P0C1X8; UniProt Database), the phosphosite Thr622 in AAK1 with 963 AA corresponds to Thr621 in AAK1 with 962 AA. The putative kinase that phosphorylates Thr621 in AAK1 is ERK1 according to ScanSite 4.0 (https://scansite4.mit.edu, accessed on 9 December 2021). The cyclin G-associated kinase (Gak) has a region between 752 and 979 amino acids with interaction sites for clathrin, AP2, and dynamin [201]. Gak was hypophosphorylated or hyperphosphorylated at both Ser824 and Ser827 after β-arrestin2 knockdown or after treatment of β-arrestin2-deficient cells with TRH (Figure 8, Tables S6 and S9).

#### 3.2.9. Alterations in Phosphorylation of Phosphoproteins Involved in Ran GTPase-Mediated Signal Transduction

RanGAP1 with the GAP activity for Ran was hyperphosphorylated at Ser427 after treatment of wild-type cells with TRH and after treatment of β-arrestin2-deficient cells with TRH or TAL (Figure 9, Tables S2, S4 and S5). This phosphosite, corresponding to Ser428 in human RanGAP1 with ID P46060, is located in the 50-amino acid sequence between 420 and 470, which is required for its SUMO-1 modification and subsequent exposure or creation of the Ranbp2 (Nup358) binding site leading to the localization of RanGAP1 at the nuclear pore complex [206]. Ranbp2 was found differentially phosphorylated at six phosphosites in three phosphorylation clusters (Figure 9, Tables S1–S5). Two phosphosites, Ser2511 and Ser2735, are located in a 455 amino acid-long segment between Ran-binding domains three and four (protein ID D4A054; UniProt Database). This segment in the Ranbp2 isoform of 3059 amino acids corresponds to a 470 amino acid long fragment in the Nup358 isoform of 3224 amino acids [206]. The phosphoresidue Ser2511 corresponds to Ser2668 in human Ranbp2 with ID P49792. It binds specifically to SUMO-1-modified RanGAP1 [206]. TRH caused hyperphosphorylation in the third cluster on Ser2088, Ser2092, Ser2096, and Ser2097, but TAL induced hypophosphorylation at Ser2092 and Ser2097 (Figure 9, Tables S2 and S3). These phosphosites are located in the region between Ran-binding domains two and three (protein ID D4A054; UniProt Database) and correspond to Ser2242, Ser2246, Ser2250, and Ser2251 in human Ranbp2 with ID P49792. The RanGAP1-Ranbp2 molecular complex is one of the key points in the signaling pathways on which TRH and TAL act in different ways.

RanGAP1 physically interacts with a RanGEF Mycbp2, inhibits its E3 ubiquitin ligase activity and transports it to the nucleus. At the same time, Mycbp2 was a weak inhibitor of the GAP activity of RanGAP1 [207]. Mycbp2 was hyperphosphorylated in two phosphorylation clusters (Ser2644 and Ser2646 in the first cluster and Thr3791 and Ser3792 in the second cluster), which were located in disordered regions after knockdown of β-arrestin2 (Figure 9, Table S1; protein ID D4A2D3; UniProt Database).

Ran-binding proteins coordinate with Ran to regulate nuclear import and export [208]. In addition to Ranbp2, other Ran-binding proteins (Ranbp1, Ranbp3, Ranbp9, and Ranbp10) were also differentially phosphorylated (Figure 9).

#### 3.2.10. Alterations in Phosphorylation of Phosphoproteins Involved in Rap GTPase-Mediated Signal Transduction

Rapgef1 (C3G) was hyperphosphorylated at Ser375 after knockdown of β-arrestin2 and hypophosphorylated at Ser239 after treatment with TAL (Figure 10, Tables S1 and S3). This protein targets members of the Ras GTPases (Rap1, Rap2, and R-Ras) and Rho GTPases [212]. Other interacting proteins include Src kinase, Grb2, and Crk. The interaction of Crk and Rapgef1 is influenced by the Cbl-b protein and the Bcr-Abl2 complex [212]. Bcr and Abl2 proteins were hyperphosphorylated after knockdown of β-arrestin2 (Figure 10, Tables S1 and S6), concomitant with phosphosite Ser375 in Rapgef1. Crk was hyperphosphorylated after treatment with TAL, concomitant with hypophosphorylation of Ser239 in Rapgef1 (Figure 10, Tables S3 and S8). Cbl-b was hypophosphorylated after β-arrestin2 knockdown and in wild-type cells treated with TRH or TAL but hyperphosphorylated in β-arrestin2-deficient cells treated with TRH or TAL (Figure 10, Tables S1–S5).

#### 3.2.11. Alterations in Phosphorylation of Phosphoproteins Involved in the β-Catenin Signaling Pathway

Stimulation of TRH-R also resulted in altered phosphorylation of phosphoproteins involved in the β-catenin signaling pathway. The β-arrestin complex with GPCR interacts with Src and EGFR or axin and GSK3β to affect phosphorylation or stabilization of β-catenin [219]. While both EGFR and Src were affected by β-arrestin2 knockdown or by treatment of these cells with TAL, the complex of axin1 and GSK3β was affected in wild-type cells treated with both agonists or in β-arrestin2-deficient cells treated with TRH (Figure 11). GSK3β was hypophosphorylated at Ser389 in β-arrestin2-deficient cells treated with TRH or in wild-type cells treated with TAL (Figure 11, Tables S3 and S4). Axin was hypophosphorylated at Ser75, Ser77, and Thr79 in wild-type cells treated with TRH or TAL and hyperphosphorylated at Ser75 and Thr79 in β-arrestin2-deficient cells after treatment with TRH (Figure 11, Tables S2–S4). The phosphoresidues Ser75, Ser77, and Thr79 in rat axin correspond to the same residues at the same positions in human axin-1 with ID O15169. Comparison of the sequences of the N-terminal segment of axin with the sequence of the detected axin with protein ID O70239 indicated that phosphosite Ser75 is located in the second peptide segment that interacts with tankyrase, an enzyme that modifies target proteins with mono- or poly-ADP-ribose [220]. Tankyrase 1-binding protein 1 (Tnks1bp1) was found to be differentially phosphorylated at nine phosphosites with distinct phosphorylation patterns for each of the five experimental groups (Figure 11 and Figure S11).

β-Catenin was differentially phosphorylated at Ser191 and Ser552, which was hypophosphorylated after β-arrestin2 knockdown or in wild-type cells treated with TAL and hyperphosphorylated in β-arrestin2-deficient cells treated with TAL (Figure 11, Table S11). Both phosphoresidues correspond to the same residues at the same positions in human β-catenin with ID P35222. β-Catenin interacts with presenilin 1 (Psen1) which inhibits β-catenin signaling [71,221]. In the present study, Psen1 was hyperphosphorylated in β-arrestin2-deficient cells treated with TRH or TAL (Figure 11, Tables S4 and S5). The altered phosphosites Ser366, Ser368, Thr371, and Ser372 are located in the 322–450 amino acid sequence required for the association of Psen1 with β-catenin [221]. While the phosphoresidues Ser366 and Ser368 correspond to Ser365 and Ser367, respectively, in human presenilin-1 with ID P49768, Thr371 and Ser372 correspond to the nonphosphorylated residues Ala370 and Gly371.

Activation of β-catenin signaling is mediated by PI3K/Akt or Wnt/Frizzled signaling pathways [222]. Frizzled receptors activated by Wnt ligand affect the GSK3β/axin complex via Dvl proteins that were differentially phosphorylated after β-arrestin2 knockdown or TRH and TAL treatments in wild-type cells (Figure 11, Tables S1–S3). The protein Dact3, a negative regulator of Wnt/β-catenin signaling via inhibition of the Dvl2 protein [223], was hyperphosphorylated at Ser6 and Ser10 after β-arrestin2 knockdown and at Ser165 after treatment of β-arrestin2-deficient cells with TRH or TAL (Figure 11, Table S11).

## 4. Discussion

In this study, we investigated the changes in the phosphoproteome induced by the activation of the TRH receptor in pituitary GH1 cells as well as in cells lacking β-arrestin2. There were hundreds of alterations in the phosphoproteome in each experimental group. Detailed analysis revealed that only some altered phosphosites were located in functional domains with enzyme activity or interaction sites with other proteins. According to the UniProt database, most phosphosites were located in disordered regions of phosphoproteins. The intrinsically disordered proteins, which lack stable tertiary structure, undergo stabilization or destabilization of transient secondary structure and more global disorder-to-order or order-to-disorder transitions upon ligand binding or post-translational modifications, including phosphorylation [227,228]. Our results support the hypothesis established by predicting protein phosphorylation sites using the web-based tool DISPHOS, which assumes that protein phosphorylations occur predominantly in intrinsically disordered protein regions [229]. The extensive alterations in the phosphoproteome of pituitary cells induced by TRH receptor activation or β-arrestin2 deficiency might be related to structural changes in phosphoproteins mediating distinct biological functions.

Although GPCRs are known to initiate signaling cascades through β-arrestin [230], this important regulator has mainly been studied in the context of TRH receptor desensitization, trafficking, and resensitization [17,231]. Interestingly, the β-arrestin–receptor interaction is not required for MAPK activation by TRH [18]. Both TRH and TAL have been shown to act via MAPK signaling [10,18]. TAL induced an increase in phosphorylation of ERK1/2 in the substantia nigra and striatum [10]. Treatment of pituitary GH3 cells with TRH at a concentration of 100 nM induced ERK phosphorylation and activation within 10 min, which lasted up to 60 min [232]. In our study, phosphorylation of ERK was not detected, which may be due to different experimental conditions, particularly a higher TRH concentration (1 μM) than in Oride’s study. However, we observed that both TRH and TAL can affect the function of the ERK upstream effector B-Raf by hypophosphorylating it. In addition, the components of the Raf/MEK/ERK scaffold, KSR2 and Cnksr1, were affected by β-arrestin2 knockdown or TAL treatment of wild-type and β-arrestin2-depleted cells (Figure 1). Comparison of amino acid sequences revealed that the phosphosite Thr276 in KSR2 (protein ID M0RBD3; UniProt Database) corresponds to Thr260 in KSR1 [233], which is one of the residues phosphorylated by activated ERK [234]. Docking of activated ERK to the KSR1 complex accelerates phosphorylation of these phosphosites in response to growth factor treatment [235], suggesting that TRH or TAL do not directly alter the phosphorylation and activity of ERK but likely affect the spatial distribution of Raf, MEK, ERK, and their scaffold via phosphorylation of their components. The distinct effect on phosphorylation of KSR2 and Cnksr1 has a knockdown of β-arrestin2, supporting the previous studies that reported the scaffold of β-arrestin with the Raf/MEK/ERK signaling cascade [236,237].

β-Arrestin interacts directly with some small GTPases and their regulatory or binding proteins [238]. It affects signaling pathways mediated by Ras, Ral, Rap, Rho, Rac, Cdc42, Arf, and Rab [30]. Our data confirm these associations and add Ran GTPase to the list (Figure 1, Figure 2, Figure 3, Figure 4, Figure 5, Figure 6, Figure 7, Figure 8, Figure 9 and Figure 10). Activation of the TRH receptor by TRH or TAL induced alterations in phosphorylation of proteins interacting with these GTPases (Figure 1, Figure 2, Figure 3, Figure 4, Figure 5, Figure 6, Figure 7, Figure 8, Figure 9 and Figure 10). Previously, only the Rho and Ras pathways were found to be involved in TRH signaling [19,20]. Our results also show that activation of the TRH receptor or absence of β-arrestin2 affects small GTPases via phosphorylation of their GEFs and GAPs (Figure 3, Figure 4, Figure 5, Figure 6, Figure 7, Figure 8, Figure 9 and Figure 10). The alterations in phosphorylation of some proteins after activation of the TRH receptor were abolished in β-arrestin2-deficient cells, e.g., B-Raf, which is scaffolded by β-arrestin2 (Figure 1), or Dvl proteins, which interact with β-arrestin (Figure 3). This effect occurred to a greater extent in the class of Rab GTPases and was observed for Rabep1, Rabep2, Rab3ip, Rab11fip5, Dennd1a, Tbd1d10a, and Ulk1 (Figure 7), suggesting that β-arrestin2 plays a role in the mechanism of endocytic and exocytic processes triggered by GPCR activation. There were few proteins for which TRH- and TAL-induced alterations in phosphorylation occurred in opposite directions in wild-type and β-arrestin2-deficient cells, such as Rptor (Figure 1), Rock2, Lats1, Myo9b, and PKD1 (Figure 3), Cbl-b and Farp2 (Figure 5), and Rab11fip1, Dennd4c, and Itsn2 (Figure 7). At the same time, there were only few proteins whose TRH- or TAL-induced alterations in phosphorylation were not affected by β-arrestin2 downregulation at least at some phosphosites, such as Arhgef28 (Figure 3), Ptk2β, Wnk2 (Figure 4), Git1, Farp2 (Figure 5), Tbc1d10b, Rabgap1 (Figure 7), Aak1 (Figure 8), and RanGAP1, and Vrk3 (Figure 9). These data suggest that β-arrestin scaffolds many cellular proteins and that its deficiency can cause major changes in the scaffolding of multiprotein complexes. Alternatively, it may be that β-arrestin assembles some proteins into multiple complexes and that the reduced scaffolding capacity of β-arrestin is reflected in components of other signaling pathways. The altered scaffolding capacity of β-arrestin affects the conformation and localization of many proteins, including protein kinases and phosphatases. This alters the accessibility of protein kinases and phosphatases to their substrates and subsequently the differential phosphorylation of proteins, leading to changes in protein function and cellular processes.

Some proteins were found to be associated with multiple small GTPases, so that there are multiple cross-talks between the signaling pathways of the members of the small GTPases. The crucial cross-talk is represented by a complex consisting of Arhgef7, Git1, and Scrib associated with Arf and Cdc42 signaling pathways that overlap with Rac and Ras signaling pathways via Lats1 (Figure 1, Figure 4, Figure 5 and Figure 6). Akt1 kinase overlaps with Ras and Arf signaling pathways (Figure 1 and Figure 6). The Ulk1, Src, and CK2β proteins connect the Ras signaling pathway to the Rab, Rho, and Rac signaling pathways, respectively (Figure 1, Figure 3, Figure 4 and Figure 7). A strongly affected Cblb protein mediates a cross-talk between the Cdc42 and Rap signaling pathways (Figure 5 and Figure 10). Another important cross-talk protein is Ralbp1 (Figure 8), a component of the Ral pathway that interacts with Ras GTPases and activates Arf6 [196].

Not only MEK and ERK, but also other MAP kinases (Map3k1, Map3k2, Map3k5, Map3k9, Map4k4) were not affected by TRH or TAL in wild-type GH1 cells. On the other hand, Map3k7 (TAK1) was slightly hypophosphorylated after TAL treatment and Map3k4 (MEKK4) and Map4k1 were markedly hypophosphorylated after TRH or TAL treatments of wild-type cells (Figure 1 and Figure 3, Table S11). All the above MAP kinases were affected in both untreated and ligand-treated β-arrestin2-deficient cells (Figure 1, Figure 2, Figure 3, Figure 4 and Figure 5), suggesting that β-arrestin2 scaffolded many MAP kinases and its deficiency altered MAPK signaling and activation induced by TRH or TAL. In addition to the Raf/MEK/ERK scaffold, β-arrestin also binds the component kinases of the JNK3 and p38 cascades, ASK1-MKK4/7-JNK3 and ASK1-MKK3/6-p38 [30]. We also detected alterations in the phosphorylation pattern of Map3k5 (ASK1) in β-arrestin2-deficient cells as well as in these cells treated with TAL (Figure 1), supporting the association of β-arrestin2 with the JNK3 and p38 cascades. The JNK1/2/3 kinase can also be activated by MEKK4 [236], whose phosphorylation was not affected by knockdown of β-arrestin2 but whose TRH/TAL-induced hypophosphorylation in wild-type cells was markedly suppressed in β-arrestin2-deficient cells (Table S11), suggesting that β-arrestin2 does not scaffold MEKK4 but mediates MEKK4 signaling triggered by TRH receptor activation. Increased expression of β-arrestin2 enhances the association of β-arrestin2 with TAK1 binding protein-1 (TAB1), leading to disruption of the TAK1-TAB1 interaction [239]. In our study, different phosphorylation patterns of Map3k7 (TAK1) were found in TAL-treated wild-type cells, in β-arrestin2-deficient cells and in TRH/TAL-treated β-arrestin2-deficient cells (Figure 3) supporting the notion that changes in β-arrestin2 expression may affect TAK1 signaling, as reflected by altered TAK1 phosphorylation. Map4k1 (HPK1) and Map4k4 (HGK) kinases are upstream kinases that phosphorylate TAK1 [235]. Map4k1 was hypophosphorylated after treatment of wild-type cells with TRH or TAL, similar to TAK1 after treatment with TAL (Figure 1), suggesting that at least TAL suppresses phosphorylation of key components of the HPK1-TAK1 pathway.

Map4k kinases were found to phosphorylate and activate Lats1/2 kinase, a component of the Hippo signaling pathway. Activated Lats1/2 in turn promotes inhibitory phosphorylation of the transcriptional co-activators YAP/TAZ [56]. Whereas Map4k4 kinase was hypophosphorylated in β-arrestin2-deficient cells and in TRH/TAL-treated wild-type cells and this phosphorylation pattern resembled phosphorylation at Ser1111 in Lats1/2, ligand binding to the TRH receptor in β-arrestin2-deficient cells abolished the phosphorylation of these two proteins (Figure 1). There is a cross-talk between the Hippo pathway and the Rho/Rac GTPase pathways. Receptors coupled to Gq/11, which include the TRH receptor, can inhibit Lats1/2 kinase activity via activation of Rho GTPase [55,56]. In addition to Map4k kinases, the Rho-ROCK-PAK or KRas-RASSF1A-MST1/2 signaling pathways can also mediate the changes in Lats1/2 activity [56]. Arhgef7 is a positive regulator of the Hippo pathway, acting as a scaffold for Lats1/2 and YAP/TAZ [127]. Some proteins from all three alternatives (Rock1, Rock2, Pak1, Pak2, MST2, and Arhgef7) were differentially phosphorylated at least in pairwise comparison (Figure 1, Figure 3 and Figure 4). Rock2 was also differentially phosphorylated in all five pairwise comparisons similar to Lats1/2, but in the opposite direction (Figure 3). The upstream KRas effectors Dclk1 and Rreb1 were also differentially phosphorylated in all five pairwise comparisons. These data suggest that multiple proteins may be involved in the regulation of Lats1/2 and that the Hippo pathway is one of the major signaling pathways affected by TRH receptor activation. To date, β-arrestins have not been found to affect the Hippo pathway. Only, re-expression of arrestin domain containing protein-3 (ARRDC3), which is structurally similar to β-arrestin, was able to attenuate GPCR-stimulated Hippo signaling [240]. β-Arrestin interacts with RhoA [238]. It appears that β-arrestin deficiency leads to disruption of the TRH receptor-mediated signaling pathway involving Gq-arrestin2-RhoA-Rock2-Lats1/2, when the signal is not transmitted from the Gq protein to RhoA. Our data support the idea that β-arrestin is a component of the Hippo signaling pathway and mediates the regulation of Lats1/2 function via its phosphorylation.

TRH has been reported to induce hyperphosphorylation of EGFR at tyrosine phosphosites and its transactivation via PLCβ/PKC or Gβγ/Src signaling pathways [241,242]. In our study, TRH or TAL did not induce alterations in phosphorylation of EGFR in wild-type cells. After knockdown of β-arrestin2, we detected hyperphosphorylation of EGFR at Ser1165 (Figure 2) but no alterations in phosphorylation of tyrosine phosphosites. Interestingly, treatment of β-arrestin2-deficient cells with TAL resulted in its hypophosphorylation (Figure 1). We detected changes in phosphorylation at Ser75 in Src (Figure 1). Hyperphosphorylation of Src is associated with its ubiquitin-dependent degradation, but its hypophosphorylation increases the availability of its active form [44]. Src hyperphosphorylation at Ser75 induced by knockdown of β-arrestin2, leading to degradation of active Src supports the idea that the interaction between β-arrestin2 and Src is constitutive [30]. β-Arrestins bind to activated GPCRs, stabilizing a state of high agonist affinity of the receptor [30]. The hypophosphorylation observed with active Src suggests that Src is active in a receptor-arrestin complex [26,30]. Here, we did not detect any alterations in Src phosphorylation state after ligand binding to the TRH receptor, but hypophosphorylation at Ser75 was found after stimulation of β-arrestin2-deficient cells, suggesting that Src is active in the TRH receptor-arrestin complex and that the amount of cellular β-arrestin2 is a critical factor determining the change in Src activity after ligand binding to the TRH receptor. β-arrestin is recruited to the endothelin-A receptor (ETAR) as an integral component of two multimeric functional complexes involved in β-catenin signaling [219]. The multimeric ETAR/β-arrestin complexes consist of Src and EGFR or axin and GSK3β and result in β-catenin Tyr phosphorylation and stabilization, respectively [219]. The phosphorylation patterns of many proteins involved in the β-catenin signaling pathway were affected in β-arrestin2-deficient cells either treated or not treated with TRH/TAL. Knockdown of β-arrestin2 resulted in hyperphosphorylation of both EGFR and Src, which was abolished in β-arrestin2-deficient cells by TAL (Figure 11). The component of the second multimeric complex, axin, was hypophosphorylated by TRH or TAL in wild-type cells and this phosphorylation was abolished by TRH treatment of β-arrestin2-deficient cells (Figure 11). These data suggest that multimeric complexes consisting of β-arrestin2/Src/EGFR or β-arrestin2/axin are affected by TRH receptor activation and that TRH or TAL might induce a different conformation of the TRH receptor manifested by different regulation of these complexes in β-arrestin2-deficient cells. The next piece of evidence suggesting that TRH receptor activation affects β-catenin signaling is the differential phosphorylation of presenilin-1 (Figure 11), whose altered phosphosites are located in the region responsible for interaction with β-catenin [221]. β-Catenin is affected by the PI3K/Akt signaling pathway [71]. Activation of the TRH receptor can lead to a translocation of β-catenin via PKAα [243]. Here, we observed that the PI3K/Akt pathway was affected when both TRH and TAL treatment of β-arrestin2-deficient cells induced differential phosphorylation of Akt and presenilin-1 (Figure 11), whereas PKAα was not altered. Akt appears to be another downstream effector of the TRH receptor that affects β-catenin signaling. The phosphosite Ser552 in β-catenin was differentially phosphorylated both after β-arrestin2 knockdown and after treatment of wild-type or β-arrestin2-deficient cells with TAL (Figure 11). β-catenin is phosphorylated at Ser552 through Akt activation downstream from EGFR signaling and/or induced by Ras/PI3K [222,224,226]. Activated PI3K/Akt cooperates with the Wnt signaling pathway to activate β-catenin signaling [222]. In the absence of a Wnt ligand, the axin scaffold facilitates GSK3β-mediated phosphorylation of cytoplasmic β-catenin, initiating its degradation [117]. In canonical Wnt signaling, Wnt ligands bind to Frizzled receptors, dishevelled (Dvl) proteins are activated, and the axin/GSK3β effect on β-catenin is prevented, leading to increased β-catenin transcriptional activity [117]. In noncanonical Wnt signaling, Wnt ligands signal to Rho and Rac GTPases via Frizzled to promote changes in the actin cytoskeleton [117]. β-Arrestins and Disheveled proteins are important mediators of Wnt signaling. In canonical Wnt signaling, β-arrestins bind to receptors via Dvl proteins to sequester axin and GSK3β from β-catenin, leading to its stabilization [117]. In noncanonical Wnt signaling, β-arrestin forms a complex with Dvl proteins and AP2 to activate RhoA and Rac1, leading to activation of Rock and JNK, respectively [117]. In our study, EGFR and Akt involved in the Ras/PI3K/Akt pathway were affected in β-arrestin2-deficient cells and after TAL treatment of β-arrestin2-deficient cells, but Dvl proteins, axin and GSK3β, were affected after TRH or TAL treatments of wild-type cells and TRH treatment of β-arrestin2-deficient cells (Figure 11). These results suggest that β-catenin signaling is differentially activated by TRH or TAL depending on the amount of β-arrestin2 and the differential TRH receptor activation.

The changes in phosphorylation of proteins involved in the Rab, Arf, and Ral signaling pathways suggest the effect of TRH, TAL, and β-arrestin2 deficiency on clathrin-dependent and -independent endocytosis and cargo transport between membrane-enclosed organelles [174,180,196]. The heterotetrameric adapter protein (AP) complexes are required at multiple endomembranes for cargo binding. AP2 is required for cargo recognition and transport between the plasma membrane and the early endosome, whereas AP3 is localized to the tubular endocytic compartment and trans-Golgi and requires Arf1 for membrane binding [160]. The AP2-coated vesicles depend on clathrin for their formation but the AP3 vesicle formation does not require clathrin [160]. In our study, the α subunit of AP2 was differentially phosphorylated only after knockdown of β-arrestin2 and treatment of β-arrestin2-deficient cells with TAL (Figure 8). The β subunit of AP3 was differentially phosphorylated after treatments with TRH or TAL, whose effects were abolished in β-arrestin2-deficient cells. The same effect was observed in AP2-interacting clathrin-endocytosis proteins (Btbd8) (Figure 6). These data suggest that both TRH and TAL affect cargo transport at the plasma membrane or Golgi and that β-arrestin2 is a key factor in mediating this effect. The AP2 complex is a component of clathrin-coated pits which have been shown to be not only isolated structures but often tightly interconnected to form clathrin plaques [244]. These plaques may be involved in adhesion or act as signaling platforms that concentrate activated GPCRs or RTKs. Clathrin-coated structures, including clathrin-coated pits and clathrin plaques, are essential for activation of several signaling pathways, including EGFR-mediated stimulation of AKT signaling, activation of the canonical Wnt pathway and β-arrestin2-mediated ERK signaling [244]. The α subunit of AP2 as well as EGFR, Akt1, components of ERK scaffold (KSR2 and Cnksr1) and β-catenin involved in the Wnt signaling pathway, were differentially phosphorylated in β-arrestin2-deficient cells and after treatment of β-arrestin2-deficient cells with TAL (Figure 1, Figure 8 and Figure 11), suggesting that β-arrestin2 functions as a component in clathrin-coated structures and that the altered phosphosites in these phosphoproteins may be regulatory sites that mediate signal transduction from clathrin-coated structures to their downstream signaling pathways.

There are two limitations in this study. The first limitation is the use of a cancer-derived cell line. It is known that cancer cell lines may alter signaling pathways. Therefore, the results of this study may correspond more to phosphosignaling processes in pituitary tumors than in normal pituitary cells. The second limitation is that β-arrestin2 silencing was not complete under our conditions. It is likely that different levels of β-arrestin2 may affect cell signaling and biological processes to different extent. Nonetheless, it is clear that deficiency of β-arrestin2 can have significant effects on biological processes, including TRH receptor-mediated signaling and it can trigger adaptive cellular processes via phosphosignaling to compensate for the changes in β-arrestin2 scaffolding and function.

Activation of the TRH receptor by TRH or TAL resulted in different phosphorylation patterns in the phosphoproteome observed in both wild-type GH1 cells and β-arrestin2-deficient cells (Figure 1, Figure 2, Figure 3, Figure 4, Figure 5, Figure 6, Figure 7, Figure 8, Figure 9, Figure 10 and Figure 11). This suggests that TRH and TAL may act at least in part as biased agonists for the TRH receptor by promoting specific signaling pathways that mediate different cellular processes and may lead to different physiological consequences. The differences between the effects of TRH and TAL may be due to lower affinity of TAL for the receptor and the lower signaling effect compared with TRH [13]. Interestingly, TAL exhibits higher activity in stimulating CNS effects than TRH [13]. Since rat pituitary cells predominantly and perhaps exclusively express the TRH-R1 subtype [15] and the CNS effects of TAL in mice are mediated primarily, if not exclusively, by TRH-R1 [245], we hypothesize, that at least the effects of TAL on phosphosignaling dynamics are mediated entirely via TRH-R1 activation, and TRH induces phosphosignaling changes predominantly via TRH-R1 activation and marginally via TRH-R2 activation, leading to the observed differences between the effects of TRH and TAL on phosphosignaling dynamics. TRH can be implicated in neurodegenerative diseases associated with aging, including Alzheimer’s disease and Parkinson’s disease [9]. TRH and its analogs, including TAL, have neuroprotective, neurotrophic, and anti-apoptotic effects [10]. We found TRH- or TAL-induced changes in some signaling pathways associated with neurodegenerative disease pathology. Alzheimer’s disease neuropathology is associated with hyperactivation of the PI3K/Akt/mTor pathway, decreased activity of AMPK, increased activity of p38 MAPK, and inhibition of the Wnt pathway [246]; whereas Parkinson’s disease neuropathology has been found to involve alterations in the autophagic/lysosomal pathway that include disruption of Rab7 function and intracellular trafficking and involvement of the p38/JNK pathway [247,248]. Our results provide the molecular background of TRH and TAL effects depending on the amount of β-arrestin2 in cells of the nervous system, which should be taken into account when thinking about potential neuroprotective effects of these ligands in the treatment of neurodegenerative diseases.

## 5. Conclusions

In the present study, TRH and TAL were found to have a strong effect on the phosphorylation of GEFs, GAPs, and other members of the small GTPase signaling pathways. They affect the phosphorylation patterns of all small GTPase classes. Simultaneously, changes in the phosphorylation of MAP kinases, Ser/Thr protein kinases, Tyr protein kinases, members of the Wnt/β-catenin and Hippo signaling pathways were detected. The phosphorylation patterns triggered by TRH and TAL were found to be different, suggesting that these ligands exhibit biased agonism at the TRH receptor. It was also shown that β-arrestin2 is a key mediator that determines TRH- and TAL-induced phosphoregulation of several phosphosignaling pathways. Downregulation of β-arrestin2 resulted in alterations in the phosphorylation of EGFR and Src kinase with potential implications for their activities. These findings may be useful in the treatment of neurodegenerative disorders and neurological pathologies with TRH and TAL. Modulators of kinase and phosphatase activities and cellular levels of β-arrestin2 can be used as additives along with TRH and TAL to support their beneficial effects.

## Data Availability

Data are contained within the article.

## Supplementary Materials

The following supporting information can be downloaded at: https://www.mdpi.com/article/10.3390/cells11091473/s1, Supplementary Results. Figure S1. Downregulation of β-arrestin2 was performed using siRNA gene silencing technology. Figures S2–S21: Quantitative changes in phosphoproteins and networks of differentially phosphorylated proteins. Table S1. A list of differentially phosphorylated proteins involved in the regulation of small GTPase activity in GH1 cells after siRNA-mediated β-arrestin2 knockdown. Table S2. A list of differentially phosphorylated proteins involved in the regulation of small GTPase activity in GH1 cells after stimulation with 1 μM TRH. Table S3. A list of differentially phosphorylated proteins related to regulation of small GTPase activity in GH1 cells after stimulation with 1 μM TAL. Table S4. A list of differentially phosphorylated proteins involved in the regulation of small GTPase activity in GH1 cells after siRNA-mediated β-arrestin2 knockdown and stimulation with 1 μM TRH. Table S5. A list of differentially phosphorylated proteins related to regulation of small GTPase activity in GH1 cells after siRNA-mediated β-arrestin2 knockdown and stimulation with 1 μM TAL. Table S6. A list of differentially phosphorylated proteins involved in MAP kinase-mediated signaling in GH1 cells after siRNA-mediated β-arrestin2 knockdown. Table S7. A list of differentially phosphorylated proteins involved in MAP kinase-mediated signaling in GH1 cells after stimulation with 1μM TRH. Table S8. A list of differentially phosphorylated proteins involved in MAP kinase-mediated signaling in GH1 cells after stimulation with 1μM TAL. Table S9. A list of differentially phosphorylated proteins involved in MAP kinase-mediated signaling in GH1 cells after siRNA-mediated β-arrestin2 knockdown and stimulation with 1 μM TRH. Table S10. A list of differentially phosphorylated proteins involved in MAP kinase-mediated signaling in GH1 cells after siRNA-mediated β-arrestin2 knockdown and stimulation with 1 μM TAL. Table S11. A list of differentially phosphorylated proteins involved in regulation of small GTPase activity and MAPK signaling in GH1 cells after siRNA knockdown of β-arrestin2 and stimulation with 1 μM TRH or TAL. References [79,89,101,162,164,186,205,211,217,213,214,249,250,251,252,253,254,255] are cited in the supplementary materials.
