# Supplementary material for: β-Arrestin2 Is Critically Involved in the Differential Regulation of Phosphosignaling Pathways by Thyrotropin-Releasing Hormone and Taltirelin
Source: Cells. 2022 Apr 27;11(9):1473. doi: 10.3390/cells11091473 (PMC9103620; doi:10.3390/cells11091473)
Supplement: Supplementary file 1 [file cells-11-01473-s001.zip › Supplementary Materials.pdf]

Supplementary Materials

β-Arrestin2 Is Critically Involved in the Differential Regulation of Phosphosignaling Pathways by Thyrotropin-releasing Hormone and Taltirelin

Zdenka Drastichova, Radka Trubacova and Jiri Novotny

I/ Supplementary Results

The paragraph numbers in this section correspond to the paragraph numbers in the Results section of the article.

S2. Alterations in Phosphorylation of Phosphoproteins Involved in GTPase-mediated Signal Transduction and Protein Phosphorylation

When the changes found in the TRH/C pairwise comparison were compared with the Arr-TRH/Arr comparison, 30 and 29 changes in phosphorylation of 22 and 21 phosphoproteins, respectively, were found in the same and opposite directions, respectively. In the pairwise comparison of TAL/C with Arr-TAL/Arr, 36 and 44 changes in phosphorylation of 21 and 35 phosphoproteins, respectively, were detected, going in the same and opposite directions, respectively. Approximately half of these phosphoproteins with changes in the same or opposite direction in pairwise comparisons included other phosphosites found only in pairwise comparisons. Many of the alterations in phosphoproteins detected in both pairwise comparisons were present only in one pairwise comparison. Some altered phosphoproteins were found in only one of four pairwise comparisons (TRH/C, Arr-TRH/Arr, TAL/C, Arr-TAL/Arr), suggesting that TRH and TAL act, at least in part, through different signaling pathways and that β-arrestin2 is an important scaffold protein regulating signaling pathways triggered by TRH or TAL.

S2.1. Alterations in Phosphorylation of Phosphoproteins Involved in Ras GTPase-mediated Signal Transduction Associated with the PI3K/Akt/mTOR Pathway

The different phosphorylation patterns in Akt1 were found in β-arrestin2-deficient cells treated with TRH or TAL. Ser126 was hypophosphorylated by both ligands, but TRH or TAL also induced hypophosphorylation at Ser124 and Ser122, respectively (Fig. 1, Tables S4 and S5). This could lead to differences in the phosphorylation patterns of downstream Akt effectors. Whereas TRH induced hypophosphorylation at Ser541 in Map3k9 in β-arrestin2-deficient cells, treatment with TAL caused hypophosphorylation at Ser1027 and Ser1031 in Map3k5 (Fig. 1, Tables S9 and S10). In β-arrestin2-deficient cells, the differences were also seen in TSC1/TSC2 and interacting proteins of the mTOR complex. TSC1 was hypophosphorylated by both ligands at Ser561 and Ser565, but TAL induced hyperphosphorylation at Ser1097 in TSC1 and TRH induced hypophosphorylation at Ser1389 in TSC2 (Fig. 1, Table S4 and S5).

While Map4k4 was hyperphosphorylated in β-arrestin2-deficient cells at Ser852 after TAL treatment (Fig. 1, Table S10), Map4k1 was differentially phosphorylated in two clusters of phosphosites with distinct phosphorylation patterns (Fig. 1, Tables S6-S8 and S10). Phosphosites Ser370 and Tyr379 were hypophosphorylated after TRH or TAL treatments in wild-type cells but Ser373 and Ser375 were hypophosphorylated after knockdown of β-arrestin2 and TRH treatment in wild-type cells and hyperphosphorylated in β-arrestin2-deficient cells treated with TAL.

S2.2. Alterations in Phosphorylation of Phosphoproteins Involved in Ras GTPase-mediated Signal Transduction Associated with the Grb2/Sos/Ras/Raf/MEK/ERK Pathway

In the present study, Ras was not differentially phosphorylated, but several associated proteins were affected. Knockdown of β-arrestin2 resulted in hyperphosphorylation at Ser821, Ser824, Ser2488,

and Thr2432 in neurofibromin (Nf1) and at Ser1114, Ser1179, Ser1180, Ser1189, and Ser1779 in afadin (Afdn) (Fig. 2, Tables S1 and S6). Treatment of β-arrestin2-deficient cells with TAL caused hyperphosphorylation at Ser177 in Rassf5 and hypophosphorylation at Ser719 in Dab2ip (Fig. 2, Table S5). Ras protein mediates signaling to the Adcy6/PKA and MEK/ERK pathways via Raf. As mentioned above, Raf was hypophosphorylated at Ser94 after treatment with TRH and TAL (Fig. 2, Tables S7 and S8). Adenylyl cyclase 6 (Adcy6) was hyperphosphorylated at Ser67 and Thr69 in β-arrestin2-deficient cells and hypophosphorylated at Ser67 and Thr69 after treatment of wild-type and β-arrestin2-deficient cells with TAL, respectively (Fig. 2, Tables S1, S3, and S5). Protein kinase A (PKA), a downstream protein of Adcy6, was differentially phosphorylated in three regulatory subunits. Whereas the Prkar1a and Prkar2a subunits were hyperphosphorylated at Ser83 and Ser97, respectively, in β-arrestin2-deficient cells, the Prkar2b subunit was hypophosphorylated at Ser83 and Ser85 in TAL-treated cells (Fig. 2, Tables S6 and S8). TRH treatment had no effect on phosphorylation of the Adcy6/PKA pathway in wild-type or β-arrestin2-deficient cells, highlighting the difference between TRH- and TAL-triggered pathways.

MEK1/2 function is influenced by Map3k2 and Map3k9 kinases [1,2]. Map3k2 kinase (MEKK2) was hyperphosphorylated in β-arrestin2-deficient cells at Ser163, Ser239, Ser331, Ser334, and Ser337 (Fig. 2, Table S6). Both TRH and TAL treatment of these cells resulted in hyperphosphorylation at Ser135 (Fig. 2, Tables S9 and S10), and TAL treatment resulted in hypophosphorylation at Ser337 (Fig. 2, Table S9). These phosphosites are located in disordered regions between the paxilin-binding domain and the kinase domain [3]. Map3k9 kinase was hyperphosphorylated in β-arrestin2-deficient cells at Ser541 and Ser545 and hypophosphorylated at Ser541 in β-arrestin2-deficient cells treated with TRH (Fig. 2, Tables S6 and S9). Both these phosphosites are located in disordered regions [4].

The kinase Rps6ka3 was hyperphosphorylated at Thr365 and Ser369 after TRH treatment in β-arrestin2-deficient cells (Fig. 2, Table S4). Both phosphosites are located in the linker region and are phosphorylated by activated ERK [5], suggesting that at least simultaneous β-arrestin2 knockdown and TRH treatment triggers ERK activation, which could be mediated by phosphorylation of Map3k2 (MEKK2) under these conditions. The Riok2 factor for pre-40S particle assembly is a substrate of Rps6ka3 [6]. In our study, it was found to be hyperphosphorylated at Ser437 after treatment with TAL of β-arrestin2-deficient cells (Fig. 2, Table S10). It is not known whether this phosphosite is phosphorylated by Rps6ka3.

Another ERK substrate, Cdk4, was hyperphosphorylated in β-arrestin2-deficient cells or TRH-treated cells at Ser300 (Fig. 2, Tables S6 and S7). The transcription factor Rreb1 was hyperphosphorylated in β-arrestin2-deficient cells at Ser1177, Ser1178, and Ser1306 (Fig. 2, Table S1) and hyperphosphorylated in β-arrestin2-deficient cells or TRH-treated cells at Ser1137, Ser1138, and Ser1590 (Fig. 2, Tables S1 and S2), hypophosphorylated at Ser1603 in TRH- or TAL-treated cells (Fig. 2, Tables S2 and S3), hyperphosphorylated at Thr1595 and Ser1597 in β-arrestin2-deficient cell treated with TRH (Fig. 2, Table S4). Phosphosites Ser1361 and Ser1364 were hypophosphorylated in β-arrestin2-deficient cells and hyperphosphorylated in β-arrestin2-deficient cells after TAL treatment (Fig. 2, Tables S1 and S5).

While knockdown of β-arrestin2 resulted in hyperphosphorylation of six phosphosites in Dclk1, treatments with TRH and TAL in wild-type or β-arrestin2-deficient cells caused hypophosphorylation with one exception, hyperphosphorylation at Ser340 in β-arrestin2-deficient cells after treatment with TAL. Three phosphosites (Ser330, Ser363, and Ser364) were differentially phosphorylated only after treatment of wild-type cells with TRH or TAL, and the phosphorylation patterns after treatments with TRH or TAL were markedly influenced by β-arrestin2 downregulation.

S2.3. Alterations in Phosphorylation of Phosphoproteins Involved in Rho GTPase-mediated Signal Transduction

Arhgef2 was hyperphosphorylated at Ser925, Ser937, Ser940, and Ser944 in β-arrestin2-deficient cells, and hypophosphorylated at Ser925, Ser937, and Ser940 after TAL treatment (Fig 3, Tables S1 and S3). All phosphosites with unknown function are located in the C-terminal region [7]. The protein Arhgef11 was differentially phosphorylated in three areas (Fig. 3), located in disordered regions outside domains [8]. Phosphosites in the first area (Ser270, Ser273, and Thr311) were hyperphosphorylated after treatment of β-arrestin2-deficient cells with TRH or TAL and hypophosphorylated in wild-type cells (Ser308) treated with TRH or TAL (Fig. 3, Tables S2-S5). Phosphosites in the second area (Ser720, Thr725, Thr729) and third area (Ser1511, Ser1512, Thr1515, and Thr1516) were hyperphosphorylated in β-

arrestin2-deficient cells (Fig. 3, Table S1). Protein Arhgef28 was differentially phosphorylated in two areas. Phosphosites Ser312 and Ser314 in the first area were hyperphosphorylated in β-arrestin2-deficient cells, and Ser314 was hypophosphorylated after treatment with TRH or TAL (Fig. 3, Tables S1-S3). Phosphosites Thr1197, Ser1198, and Ser1200 were hypophosphorylated in wild-type and β-arrestin2-deficient cells treated with TRH or TAL (Fig. 3, Tables S2-S5). The phosphorylation patterns of Arhgef12 and Arhgef40 were affected only by TAL treatment (Fig. 3, Table S1, S3, and S5). The phosphosites in Arhgef12, Arhgef28, and Arhgef40 were determined to be located in disordered regions by comparison with the amino acid sequence and the position of the functional domains according to the protein IDs in the UniProt database. Their functions are not known.

Dishevelled proteins (Dvl1, Dvl2, and Dvl3), Camk2β, and Vav2 were markedly phosphorylated by TRH or TAL treatments of wild-type cells (Fig. 3, Tables S2 and S3). Intersectin-1 (Itsn1) appears to be a key protein, as it was differentially phosphorylated in all five paired experimental groups (Fig. 3, Tables S1-S5).

Some of the Rho downstream effectors (e.g. Rock1, Snrk, Pak6, PKCδ, PKD2) were differentially phosphorylated only in β-arrestin2-deficient cells (Fig. 3, Tables S1 and S6). Only one phosphosite was found in the functional domain, phosphosite Ser711 in PKD2 in the protein kinase domain [9]. Map3k5 (ASK1) was affected with or without TAL in β-arrestin2-deficient cells (Fig. 3, Tables S6 and S10). Map3k20 (Zak) was hypophosphorylated at Ser434 and Ser452 after treatment with TRH or TAL (Fig. 3, Tables S7 and S8). Both kinases were affected with the same phosphorylation pattern at their altered phosphosites. Treatment with TAL decreased the level of phosphorylation at Ser439 in Map3k7, but β-arrestin2 knockdown induced hyperphosphorylation at its phosphosites Ser439 and Ser454 (Fig. 3, Tables S6, S8-10). In addition to PKCδ and PKD2, other protein kinases were also differentially phosphorylated, and not only in β-arrestin2-deficient cells.

In protein kinase D1, the first cluster (Ser161 and Ser164) was hyperphosphorylated in β-arrestin2-deficient cells and hypophosphorylated after treatment with TAL (Fig. 3, Tables S6 and S8). The second cluster (Ser189 and Ser192) was identically hypophosphorylated in wild-type cells treated with TRH or TAL (Fig. 3, Tables S7 and S8), whereas phosphosites in the third cluster (Ser361 and Thr364) were identically hypophosphorylated or hyperphosphorylated only after TRH treatment or after TRH or TAL treatments of β-arrestin2-deficient cells, respectively (Fig. 3, Tables S6-S10).

S2.4. Alterations in Phosphorylation of Phosphoproteins Involved in Rac GTPase-mediated Signal Transduction

In contrast to TAL treatment, TRH treatment induced changes in the phosphorylation patterns of Dvl3 and Marcks (Fig. 4, Table S2). In β-arrestin2-deficient cells, treatment with TRH or TAL affected the phosphorylation of other GEFs such as Tiam1, Mcf2l, Prex2, and Dock6 (Fig. 4, Tables S4 and S5).

The GAPs Myo9b and Arhgap35 affected both Rho and Rac GTPases [10]. Three other GAPs, Arhgap17, Arhgap35, and Farp2, were hypophosphorylated after treatment with TRH or TAL (Fig. 4, Tables S2 and S3), but both ligands induced hypophosphorylation of Farp2 and Arhgap35 in β-arrestin2-deficient cells (Fig. 4, Tables S4 and S5). In contrast to TRH, treatment of β-arrestin2-deficient cells with TAL did not affect the phosphorylation state of Arhgap 17 (Fig. 4, Tables S4 and S5).

Srpk1 kinase was significantly affected in three phosphorylation clusters. The first cluster, comprising Ser33, Ser37, Ser39, and Ser51, was hypophosphorylated after TRH treatment. Phosphosite Ser51 was also hypophosphorylated in TAL-treated cells and after treatment of β-arrestin2-deficient cells with TRH or TAL (Fig. 4, Tables S2-S5). The second cluster, involving Ser309 and Ser311, was hyperphosphorylated after β-arrestin knockdown or after treatment of β-arrestin2-deficient cells with TAL (Fig. 4, Tables S1 and S5). The third cluster, involving Thr453 and Ser455, was hypophosphorylated after treatment of wild-type cells with TRH or TAL (Fig. 4, Tables S2-S3). In contrast, Srpk2 was differentially phosphorylated only in β-arrestin2-deficient cells before and after treatment with TRH or TAL (Fig. 4, Tables S1, S4, and S5).

S2.5. Alterations in Phosphorylation of Phosphoproteins Involved in Cdc42 GTPase-mediated Signal Transduction

The altered Pak kinases and Map3k7 are downstream effectors that are also typical for other members of the Rho class (Figs. 3-5). Both Cdc42bpa and Cdc42bpb were differentially phosphorylated at their C-termini outside the functional domains (protein IDs A0A0G2K5Z1 and Q7TT49; UniProt database). Both were hyperphosphorylated in β-arrestin2-deficient cells (Fig. 5, Table S1), whereas

Cdc42bpa or Cdc42bpb were hypophosphorylated in β-arrestin2-deficient cells treated with TAL or in wild-type cells treated with TRH or TAL (Fig. 5, Tables S3 and S5). Phosphosite Ser116 in Cdc42ep4 was hyperphosphorylated in β-arrestin2-deficient cells after treatment with TRH or TAL (Fig. 5, Tables S4-S5).

S2.6. Alterations in Phosphorylation of Phosphoproteins Involved in Arf GTPase-mediated Signal Transduction

Arfgef1 was hypophosphorylated in the first and second clusters after treatment with TAL and only at Ser394 in the second cluster after treatment with TRH. The different phosphorylation patterns were found after TRH or TAL treatment of β-arrestin2-deficient cells (Fig. 6, Tables S1-S5). Arfgef2 was hyperphosphorylated in β-arrestin2-deficient cells in all three clusters and hypophosphorylated in the first cluster after treatment with TRH or TAL (Fig. 6, Tables S1-S3). Arfgef3 was hypophosphorylated in β-arrestin2-deficient cells treated with TRH or TAL (Fig. 6, Tables S4-S5).

Four Arf GAPs (Arfgap2, Asap1, Asap2, and Agfg1) were hypophosphorylated in wild-type cells after treatment with TAL (Fig. 6, Table S3) and only Agfg1 was hypophosphorylated in wild-type cells after treatment with TRH (Fig. 6, Table S2). Two proteins (Asap1 and Arap1) were hyperphosphorylated in β-arrestin2-deficient cells after treatment with TRH or TAL (Fig. 6, Tables S4 and S5). These data suggest that β-arrestin knockdown abolished the TRH/TAL effect on hypophosphorylation of Arf-GAPs, which could lead to a change in their GAP activities.

Arfip1 (arfaptin-1), an interacting protein for Arf [11], was hypophosphorylated at the N-terminus in wild-type cells after treatment with TRH or TAL and in β-arrestin2-deficient cells treated with TRH (Fig. 6, Tables S2-S4). Bin1, which forms a complex with Arf6 and the GluA1 receptor [12], was hyperphosphorylated after β-arrestin2 knockdown and hypophosphorylated in wild-type cells after treatment with TRH and TAL and in β-arrestin2-deficient cells after treatment with TRH (Fig. 6, Tables S1-S4).

S2.7. Alterations in Phosphorylation of Phosphoproteins Involved in Rab GTPase-mediated Signal Transduction

Phosphosites in the first cluster were hyperphosphorylated after knockdown of β-arrestin2 and hypophosphorylated in wild-type cells after treatment with TAL (Fig. 7, Tables S1 and S5). The second cluster (Thr972, Ser973, Ser978) was hypophosphorylated after treatment with TAL, and the hypophosphorylation was more pronounced in β-arrestin2-deficient cells than in wild-type cells (Fig. 7, Tables S3 and S5). In the case of PDZD8, β-arrestin2 deficiency deepened the TAL effect on hypophosphorylation.

The Rab8-GEF, Rab3ip, was hypophosphorylated at Ser272 in wild-type cells after treatment with TRH or TAL (Fig. 7, Tables S2 and S3). Dennd4c, which has GEF activity toward Rab10 [13], was hypophosphorylated after β-arrestin2 knockdown and TRH or TAL treatments of wild-type cells but hyperphosphorylated in β-arrestin2-deficient cells treated with TAL (Fig. 7, Tables S1-S3 and S5), suggesting that β-arrestin2 knockdown reverses the TAL effect on Dennd4c phosphorylation. Dennd5a was hyperphosphorylated after β-arrestin2 knockdown at Thr1079 and Ser1085 but hypophosphorylated in β-arrestin2-deficient cells after treatment with TAL (Fig. 7, Tables S1 and S5).
Tbc1d4 and Tbc1d25, which interact with several Rab proteins associated with the Golgi, were hyperphosphorylated in β-arrestin2-deficient cells (Fig. 7, Table S1).

Madd possesses a DENN domain, which is also found in Dennd proteins, and GEF activity for both Rab3 and Rab27 [13]. In our study, this protein was differentially phosphorylated at two phosphosites, Ser828 and Ser1196, both located in disordered regions (protein ID O08873; UniProt Database). Both phosphosites exhibit very different phosphorylation patterns. Whereas Ser828 was hypophosphorylated after TRH or TAL treatments in wild-type cells, Ser1196 was hypophosphorylated after β-arrestin2 knockdown and hyperphosphorylated after TRH and TAL treatments of β-arrestin2-deficient cells (Fig. 7, Tables S1-S5).

S2.8. Alterations in Phosphorylation of Phosphoproteins Involved in Ral GTPase-mediated Signal Transduction

RalGAP, which inactivates Ral GTPases, is a heterodimeric complex consisting of an α (RalGAPa1 or RalGAPa2) catalytic subunit and a β regulatory subunit (RalGAPb) [14]. The RalGAPa1 and RalGAPb subunits were found to be hyperphosphorylated after β-arrestin2 knockdown, RalGAPa2 was hypophosphorylated after treatment with TAL and RalGAPb was hypophosphorylated in β-arrestin2-deficient cells after treatment with TRH (Fig. 8, Tables S1, S3, and S4).

S2.9. Alterations in Phosphorylation of Phosphoproteins Involved in Ran GTPase-mediated Signal Transduction

Only Ranbp10 was affected by TRH and TAL treatments in wild-type cells. The opposite effect on phosphorylation was observed after TRH treatment of β-arrestin2-deficient cells (Fig. 9, Tables S2-S4). Ranbp1, Ranbp3, and Ranbp9 were differentially phosphorylated after β-arrestin2 knockdown and after treatment of β-arrestin2-deficient cells with TRH or TAL (Fig. 9, Tables S1, S4, and S5).

Vrk3 has been shown to interact with Ran GTPases [15]. It was differentially phosphorylated after β-arrestin2 knockdown, in wild-type cells treated with TAL, and in β-arrestin2-deficient cells treated with TRH or TAL (Fig. 9, Tables S6 and S8-S10). Phosphosites Ser82, Ser83, and Ser85 form the first phosphorylation cluster and Ser88 the second (Fig. 9).

S2.10. Alterations in Phosphorylation of Phosphoproteins Involved in Rap GTPase-mediated Signal Transduction

Rapgef6 was mainly affected in β-arrestin2-deficient cells after treatment with TAL. Other phosphorylation patterns were also found after β-arrestin2 knockdown and in wild-type or β-arrestin2-deficient cells treated with TRH (Fig. 10, Tables S1, S2, S3, and S5).

Rap1gap2, which has GTPase-stimulating activity toward Rap1 [16], was hypophosphorylated after knockdown of β-arrestin2 at Ser361, as it was in wild-type cells treated with TRH. On the other hand, treatment of β-arrestin2-deficient cells with TRH or TAL induced hyperphosphorylation of this phosphosite (Fig. 10, Tables S1, S2, S4, and S5). The phosphosite Ser365 was hypophosphorylated after treatment of wild-type cells with TRH (Fig. 10, Tables S2), suggesting that only Ser361 is affected by β-arrestin2 knockdown.

The protein Sipa1l1 with RapGAP activity was affected by treatment of wild-type cells with TRH, but β-arrestin2 knockdown resulted in effects of both ligands on its phosphorylation state (Fig. 10, Tables S1, S2, S4, and S5). Radil protein, which interacts with Rap GTPase [17], was hypophosphorylated after treatment of wild-type cells with TRH or TAL (Fig. 10, Tables S2 and S3). Tnik, a downstream effector of Rap2 [18], was hypophosphorylated in wild-type cells after treatment with TAL (Fig. 10, Table S3).

References

1. Bok, S.; Shin, D.Y.; Yallowitz, A.R.; Eiseman, M.; Cung, M.; Xu, R.; Li, N.; Sun, J.; Williams, A.L.; Scott, J.E.; et al. MEKK2 mediates aberrant ERK activation in neurofibromatosis type I. *Nat. Commun.* **2020**, *11*, doi:10.1038/s41467-020-19555-6.
2. Fawdar, S.; Trotter, E.W.; Li, Y.Y.; Stephenson, N.L.; Hanke, F.; Marusiak, A.A.; Edwards, Z.C.; Ientile, S.; Waszkowycz, B.; Miller, C.J.; et al. Targeted genetic dependency screen facilitates identification of actionable mutations in FGFR4, MAP3K9, and PAK5 in lung cancer. *Pro. Natl Acad. Sci. U. S. A.* **2013**, *110*, 12426-12431, doi:10.1073/pnas.1305207110.
3. Kahle, M.P.; Cuevas, B.D. Interaction with the Paxillin LD1 Motif Relieves MEKK2 Auto-inhibition. *J. Mol. Signal.* **2015**, *10*, 4, doi:10.5334/1750-2187-10-4.
4. Stark, M.S.; Woods, S.L.; Gartside, M.G.; Bonazzi, V.F.; Dutton-Regester, K.; Aoude, L.G.; Chow, D.; Sereduk, C.; Niemi, N.M.; Tang, N.Y.; et al. Frequent somatic mutations in MAP3K5 and MAP3K9 in metastatic melanoma identified by exome sequencing. *Nat. Gen.* **2012**, *44*, 165-169, doi:10.1038/ng.1041.
5. Vaidyanathan, H.; Opoku-Ansah, J.; Pastorino, S.; Renganathan, H.; Matter, M.L.; Ramos, J.W. ERK MAP kinase is targeted to RSK2 by the phosphoprotein PEA-15. *Proc. Natl. Acad. Sci. U. S. A.* **2007**, *104*, 19837-19842, doi:10.1073/pnas.0704514104.
6. Cerezo, E.L.; Houles, T.; Lie, O.; Sarthou, M.K.; Audoynaud, C.; Lavoie, G.; Halladjian, M.; Cantaloube, S.; Froment, C.; Burlet-Schiltz, O.; et al. RIOK2 phosphorylation by RSK promotes synthesis of the human small ribosomal subunit. *Plos Gen.* **2021**, *17*, doi:10.1371/journal.pgen.1009583.
7. Ravindran, E.; Hu, H.; Yuzwa, S.A.; Hernandez-Miranda, L.R.; Kraemer, N.; Ninnemann, O.; Musante, L.; Boltshauser, E.; Schindler, D.; Hubner, A.; et al. Homozygous ARHGEF2 mutation causes intellectual disability and midbrain-hindbrain malformation. *Plos Gen.* **2017**, *13*, doi:10.1371/journal.pgen.1006746.

8. Lee, S.; Cieply, B.; Yang, Y.Q.; Peart, N.; Glaser, C.; Chan, P.; Carstens, R.P. Esrp1-Regulated Splicing of Arhgef11 Isoforms Is Required for Epithelial Tight Junction Integrity. *Cell Rep.* **2018**, *25*, 2417-+, doi:10.1016/j.celrep.2018.10.097.
9. Rykx, A.; De Kimpe, L.; Mikhalap, S.; Vantus, T.; Seufferlein, T.; Vandenheede, J.R.; Van Lint, J. Protein kinase D: a family affair. *FEBS Lett.* **2003**, *546*, 81-86, doi:10.1016/s0014-5793(03)00487-3.
10. Muller, P.M.; Rademacher, J.; Bagshaw, R.D.; Wortmann, C.; Barth, C.; van Unen, J.; Alp, K.M.; Giudice, G.; Eccles, R.L.; Heinrich, L.E.; et al. Systems analysis of RhoGEF and RhoGAP regulatory proteins reveals spatially organized RAC1 signalling from integrin adhesions. *Nat. Cell Biol.* **2020**, *22*, doi:10.1038/s41556-020-0488-x.
11. Sztul, E.; Chen, P.W.; Casanova, J.E.; Cherfils, J.; Decks, J.B.; Lambright, D.G.; Lee, F.J.S.; Randazzo, P.A.; Santy, L.C.; Schurmann, A.; et al. ARF GTPases and their GEFs and GAPs: concepts and challenges. *Mol. Biol. Cell* **2019**, *30*, 1249-1271, doi:10.1091/mbc.E18-12-0820.
12. Schürmann, B.; Bermingham, D.P.; Kopeikina, K.J.; Myczek, K.; Yoon, S.; Horan, K.E.; Kelly, C.J.; Martin-de-Saavedra, M.D.; Forrest, M.P.; Fawcett-Patel, J.M.; et al. A novel role for the late-onset Alzheimer's disease (LOAD)-associated protein Bin1 in regulating postsynaptic trafficking and glutamatergic signaling. *Mol. Psychiatry* **2020**, *25*, 2000-2016, doi:10.1038/s41380-019-0407-3.
13. Marat, A.L.; Dokainish, H.; McPherson, P.S. DENN Domain Proteins: Regulators of Rab GTPases. *Journal of Biological Chemistry* **2011**, *286*, 13791-13800, doi:10.1074/jbc.R110.217067.
14. Personnic, N.; Lakisic, G.; Gouin, E.; Rousseau, A.; Gautreau, A.; Cossart, P.; Bierne, H. A role for Ral GTPase-activating protein subunit beta in mitotic regulation. *FEBS J.* **2014**, *281*, 2977-2989, doi:10.1111/febs.12836.
15. Sanz-Garcia, M.; Lopez-Sanchez, I.; Lazo, P.A. Proteomics Identification of Nuclear Ran GTPase as an Inhibitor of Human VRK1 and VRK2 (Vaccinia-related Kinase) Activities. *Mol. Cell. Prot.* **2008**, *7*, 2199-2214, doi:10.1074/mcp.M700586-MCP200.
16. Schultess, J.; Danielewski, O.; Smolenski, A.P. Rap1GAP2 is a new GTPase-activating protein of Rap1 expressed in human platelets. *Blood* **2005**, *105*, 3185-3192, doi:10.1182/blood-2004-09-3605.
17. Smolen, G.A.; Schott, B.J.; Stewart, R.A.; Diederichs, S.; Muir, B.; Provencher, H.L.; Look, A.T.; Sgroi, D.C.; Peterson, R.T.; Haber, D.A. A Rap GTPase interactor, RADIL, mediates migration of neural crest precursors. *Genes Dev.* **2007**, *21*, 2131-2136, doi:10.1101/gad.1561507.
18. Chen, X.; Shibata, A.C.E.; Hendi, A.; Kurashina, M.; Fortes, E.; Weilinger, N.L.; MacVicar, B.A.; Murakoshi, H.; Mizumoto, K. Rap2 and TNIK control Plexin-dependent tiled synaptic innervation in C. elegans. *Elife* **2018**, *7*, doi:10.7554/eLife.38801.

II/ Supplementary Figures and Tables

Figure S1. Downregulation of β-arrestin2 was performed using siRNA gene silencing technology. To suppress the expression of β-arrestin2 (β-Arr2), GH1 cells were transfected with the appropriate siRNA as described in Materials and Methods. Two days after transfection, total cell lysates (20 µg per lane) were subjected to SDS-PAGE and immunoblotted with β-Arr2 (A) or β-Arr1 (B) antibody. Transfection of GH1 cells with nonsilencing (CTRL) or specific siRNA is indicated at the top of each immunoblot (middle panel of the figure). Signal intensities were normalized to total protein by staining membranes with Ponceau S (middle panel of the figure). Transfection of GH1 cells with β-arrestin2 resulted in a decrease of more than 60% in the expression of β-arrestin2 and, in parallel, there was no significant change in β-arrestin1 (lower panel of the figure). Values represent the mean ± S.E.M. (**, $p < 0.01$ compared with CTRL) of three independent experiments.

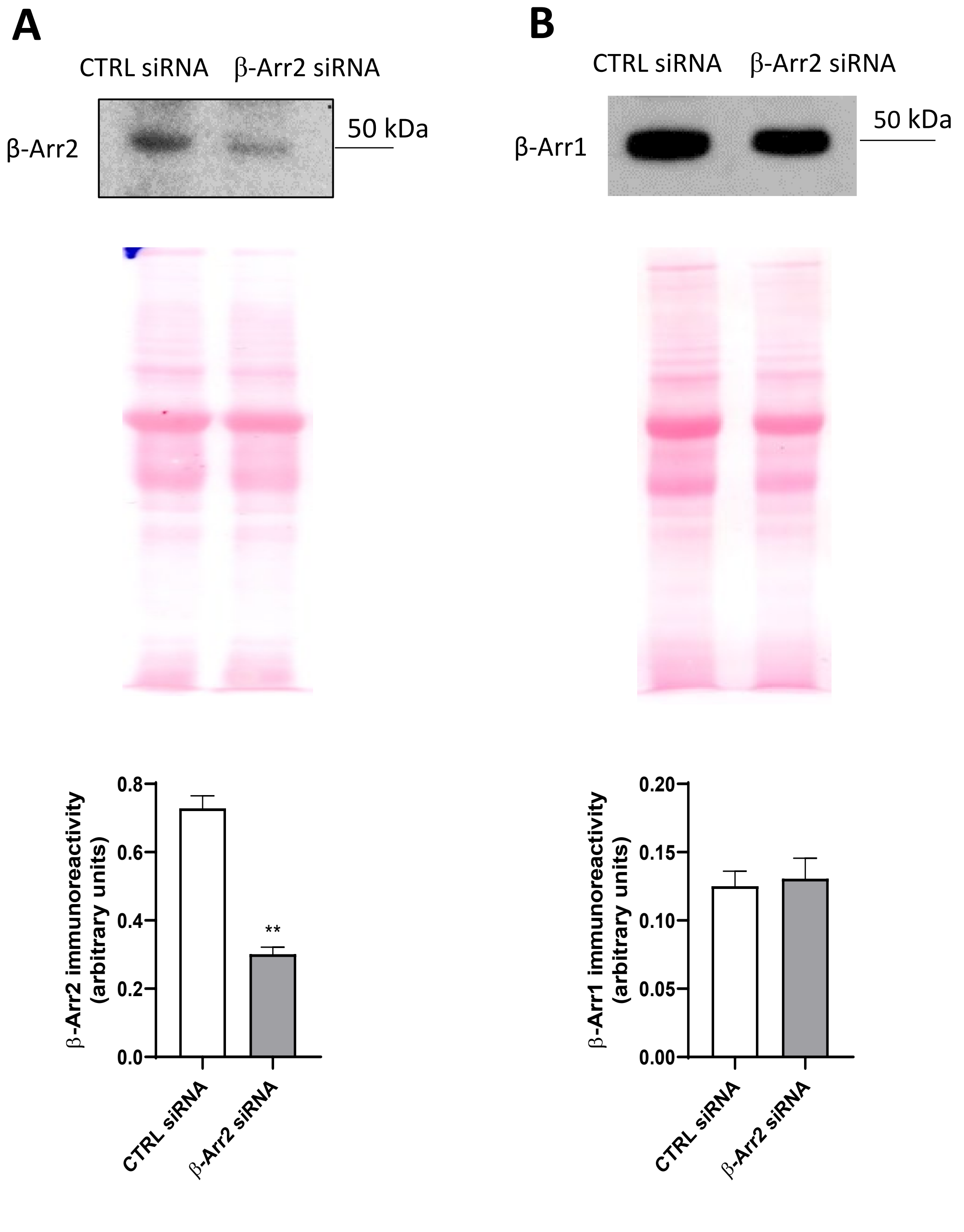

Table S1. A list of differentially phosphorylated proteins involved in the regulation of small GTPase activity in GH1 cells after siRNA-mediated β-arrestin2 knockdown

Uniprot ID	Gene ID	Gene name	Phosphorylated regions
F1LRI7	Aak1	AP2 associated kinase 1	Thr608 ↑[12.88] (_1)
F1M0N1	Abl2	ABL proto-oncogene 2, non-receptor tyrosine kinase	Ser629 ↑[70.75] (_1)
A0A0G2K429	Adcy6	adenylate cyclase 6	Ser67 ↑[37.83] (_1), Thr69 ↑[AR] (_1)
O35889	Afdn	afadin, adherens junction formation factor	Ser1114 ↑[15.02] (_1), Ser1179 ↑[33.60] (_3), Ser1180 ↑[33.60] (_3), Ser1189 ↑[33.60] (_3), Ser1779 ↑[4.40] (_1)
F1M9N7	Agfg1	ArfGAP with FG repeats 1	Thr177 ↑[65.90] (_2), Thr179 ↑[AR] (_2), Ser181 ↑[84.71] (_2)
P47196	Akt1	AKT serine/threonine kinase 1	Ser124 ↑[169.68] (_2), Ser 124 ↑[93.14] (_1), Ser126 ↑[AR] (_2), Ser126 ↑[155.76] (_3), Ser129 ↑[155.76] (_3)
P0C5Y8	Als2	alsin Rho guanine nucleotide exchange factor	Ser477 ↑[9.23] (_2), Ser486 ↑[9.67] (_2)
Q3MID3	Arfgap2	ADP-ribosylation factor GTPase activating protein 2	Ser431 ↑[AR] (_1)
Q4KLN7	Arfgap3	ADP-ribosylation factor GTPase activating protein 3	Ser332 ↑[17.40] (_1)
D4A631	Arfgef1	ADP ribosylation factor guanine nucleotide exchange factor 1	Ser393 ↓[C] (_2), Ser394 ↓[C] (_2), Ser1563 ↑[AR] (_2), Ser1566 ↑[AR] (_2)
Q7TSU1	Arfgef2	ADP ribosylation factor guanine nucleotide exchange factor 2	Ser218 ↑[32.01] (_2), Ser355 ↑[AR] (_2), Ser356 ↑[AR] (_2), Ser1518 ↑[AR] (_2), Ser1520 ↑[AR] (_2), Ser1521 ↑[AR] (_2)
A0A0G2JYA2	Arfip1	ADP-ribosylation factor interacting protein 1	Ser9 ↑[AR] (_1)
D4A6C5	Arhgap1	Rho GTPase activating protein 1	Ser51 ↑[AR] (_2)
Q6TUE6	Arhgap5	Rho GTPase activating protein 5	Thr1171 ↑[AR] (_2), Ser1173 ↑[5.30] (_2), Ser1176 ↑[5.30] (_2), Thr1217 ↑[191.85] (_1)
D4AAV4	Arhgap17	Rho GTPase activating protein 17	Ser574 ↑[16.53] (_1), Thr748 ↑[74.11] (_2), Ser751 ↑[74.11] (_2), Thr752 ↓[C] (_2) ↑[AR] (_3)
F1M2D4	Arhgap23	Rho GTPase activating protein 23	Ser341 ↑[AR] (_2), Ser352 ↑[AR] (_2)
F1MAK3	Arhgap32	Rho GTPase activating protein 32	Ser720 ↑[AR] (_2), Ser723 ↑[AR] (_2), Ser870 ↑[18.24] (_1), Ser966 ↑[AR] (_1)
D4AD82	Arhgap35	Rho GTPase activating protein 35	Ser773 ↑[AR] (_2), Ser975 ↑[14.54] (_2), Ser985 ↑[13.43] (_2), Ser1127 ↑[AR] (_2), Ser1176 ↑[AR] (_2), Ser1179 ↑[41.86] (_1)
A0A1B0GWY5	Arhgef2	Rho/Rac guanine nucleotide exchange factor 2	Ser925 ↑[9.67] (_3), Ser937 ↑[9.08] (_2), Ser940 ↑[8.32] (_2), Ser944 ↑[9.82] (_3)
A0A0G2QC21	Arhgef7	Rho guanine nucleotide exchange factor 7	Ser497 ↑[AR] (_1)
A0A0G2JZC6	Arhgef11	Rho guanine nucleotide exchange factor 11	Ser720 ↑[7.48] (_3), Thr725 ↑[7.48] (_3), Thr729 ↑[7.48] (_3), Ser1511 ↑[AR] (_2), Ser1512 ↑[AR] (_2), Thr1515 ↑[AR] (_2), Thr1516 ↑[AR] (_2)
D3ZYR0	Arhgef12	Rho guanine nucleotide exchange factor 12	Ser 341 ↓[C] (_1), Ser1176 ↑[AR] (_1)
P0C6P5	Arhgef28	Rho guanine nucleotide exchange factor 28	Ser312 ↑[AR] (_1), Ser314 ↑[12.33] (_1)

A0A0G2JZE7	Arhgef40	Rho guanine nucleotide exchange factor 40	Ser958 ↑[AR] (_1)
Q4V8I5	Arl6ip4	ADP-ribosylation factor like GTPase 6 interacting protein 4	Ser142 ↓[C] (_1)
A0A0G2K451	Asap1	ArfGAP with SH3 domain, ankyrin repeat and PH domain 1	Ser852 ↑[AR] (_2), Ser855 ↑[AR] (_2)
B2GUV8	Bcl6	B-cell CLL/lymphoma 6	Ser308 ↑[AR] (_2), Ser309 ↑[AR] (_2)
F1LXF1	Bcr	BCR, RhoGEF and GTPase activating protein	Ser253 ↑[AR] (_2), Ser256 ↑[AR] (_2)
F1LMX1	Bin1	bridging integrator 1	Ser299 ↑[477.71] (_2), Ser305 ↑[13.43] (_2), Ser325 ↑[36.60] (_2)
D3ZML2	Brsk2	BR serine/threonine kinase 2	Ser424 ↑[13.84] (_2), Ser428 ↓[C] (_3) ↑[13.84] (_2), Ser436 ↓[C] (_3) ↑[AR] (_2), Ser440 ↑[AR] (_2), Ser490 ↑[AR] (_1)
Q8K4S7	Cblb	Cbl proto-oncogene B	Ser476 ↓[C] (_3), Ser480 ↓[C] (_3), Ser483 ↓[C] (_3), Ser484 ↓[C] (_2)
A0A0G2K5Z1	Cdc42bpa	CDC42 binding protein kinase alpha	Ser1618 ↑[AR] (_3), Ser1622 ↑[AR] (_2), Ser1625 ↑[AR] (_2)
Q7TT49	Cdc42bpb	CDC42 binding protein kinase beta	Ser1688 ↑[5.81] (_3), Ser1692 ↑[5.81] (_3), Ser1695 ↑[5.81] (_3)
Q6P751	Cdk2	cyclin dependent kinase 2	Thr14 ↑[134.24] (_2), Tyr15 ↑[134.24] (_2)
E2E1S0	Cdkl5	cyclin-dependent kinase-like 5	Ser407 ↑[17.43] (_1)
G3V894	Chrm4	cholinergic receptor, muscarinic 4	Ser246 ↑[AR] (_2), Ser252 ↑[AR] (_2)
G3V8W8	Cnksr1	connector enhancer of kinase suppressor of Ras 1	Thr284 ↑[AR] (_2), Ser288 ↑[200.85] (_2)
A0A1B0GWS4	Cttn	cortactin	Tyr139 ↓[C] (_2)
A0A0G2JTF2	Dab2ip	DAB2 interacting protein	Ser719 ↑[AR] (_1)
A0A0G2KB92	Dclk1	doublecortin-like kinase 1	Ser305 ↑[AR] (_2), Ser307 ↑[AR] (_2), Ser332 ↑[AR] (_3), Thr336 ↑[AR] (_2), Ser337 ↑[AR] (_3)
F1M241	Dennd1a	DENN domain containing 1A	Ser521 ↑[4.07] (_2)
F1LTD7	Dennd4c	DENN domain containing 4C	Ser1310 ↓[C] (_2), Ser1323 ↑[52.69] (_2), Ser1336 ↑[52.69] (_2)
G3V7Q0	Dennd5a	DENN domain containing 5A	Thr1079 ↑[AR] (_2), Ser1085 ↑[AR] (_2)
D4A544	Dennd6a	DENN domain containing 6A	Ser13 ↑[AR] (_2), Ser16 ↑[AR] (_1), Ser16 ↑[AR] (_2)
A0A0G2K9I2	Dlc1	DLC1 Rho GTPase activating protein	Ser163 ↑[AR] (_1)
A0A0G2KAH4	Dock6	dedicator of cytokinesis 6	Ser1409 ↑[AR] (_1)
F1LRS2	Dock7	dedicator of cytokinesis 7	Ser904 ↑[72.55] (_2), Ser906 ↑[72.55] (_2), Ser1408 ↑[28.12] (_1), Ser1410 ↑[AR] (_2), Ser1416 ↑[22.61] (_1)
Q9WVB9	Dvl1	dishevelled segment polarity protein 1	Ser194 ↑[AR] (_1)
D3ZB71	Dvl2	dishevelled segment polarity protein 2	Ser211 ↓[C] (_1)
D4ADV8	Dvl3	dishevelled segment polarity protein 3	Ser48 ↑[AR] (_1)
G3V6K6	Egfr	epidermal growth factor receptor	Ser1165 ↑[AR] (_1)
Q07205	Eif5	eukaryotic translation initiation factor 5	Ser387 ↑[66.50] (_2), Ser388 ↑[66.50] (_2), Ser417 ↑[AR] (_1)
F7DLY1	Eps8l2	EPS8-like 2	Ser482 ↑[AR] (_1), Ser483 ↑[AR] (_1)
B5DFE2	Ezh2	enhancer of zeste 2 polycomb repressive complex 2 subunit	Ser362 ↑[AR] (_2), Ser363 ↑[AR] (_2)
Q9JHY1	F11r	F11 receptor	Ser285 ↑[AR] (_2), Ser288 ↑[AR] (_2)

F1LYQ8	Farp1	FERM, ARH/RhoGEF and pleckstrin domain protein 1	Thr371 ↑[AR] (_2), Ser373 ↑[AR] (_2), Ser893 ↑[16.79] (_3), Ser900 ↑[16.67] (_3), Ser903 ↑[64.70] (_3)
D3ZFK8	Farp2	FERM, ARH/RhoGEF and pleckstrin domain protein 2	Thr374 ↑[AR] (_2), Ser375 ↑[AR] (_2), Thr378 ↑[AR] (_2), Ser474 ↑[AR] (_2), Ser477 ↓[C] (_2), Ser511 ↑[AR] (_2)
Q2HWF0	Fnbp1l	formin binding protein 1-like	Ser501 ↑[AR] (_3) ↓[C] (_1), Ser505 ↑[3.13] (_2)
D4A022	Gapvd1	GTPase activating protein and VPS9 domains 1	Ser466 ↑[25.30] (_2), Ser785 ↑[AR] (_2), Ser788 ↑[65.34] (_2), Thr789 ↑[65.34] (_2), Ser971 ↑[60.26] (_2), Ser802 ↑[AR] (_3)
A0A0G2K3N1	Gbf1	golgi brefeldin A resistant guanine nucleotide exchange factor 1	Ser340 ↑[AR] (_2), Ser345 ↑[19.84] (_2), Ser1293 ↑[AR] (_1)
A0A0G2K527	Git1	GIT ArfGAP 1	Ser376 ↑[AR] (_2), Ser376 ↑[AR] (_3), Thr383 ↑[AR] (_2), Ser583 ↑[AR] (_2), Ser587 ↑[AR] (_2), Tyr589 ↑[AR] (_2)
Q66H91	Git2	GIT ArfGAP 2	Tyr392 ↑[AR] (_2), Ser394 ↓[C] (_3)
Q9WVE9	Itsn1	intersectin 1	Ser334 ↑[8.48] (_2), Ser335 ↑[8.48] (_2), Ser894 ↓[C] (_2), Ser896 ↑[AR] (_1)
M0R7A6	Itsn2	intersectin 2	Ser231 ↓[C] (_1), Ser908 ↓[C] (_1)
F1LZV1	Kalrn	kalirin, RhoGEF kinase	Ser1790 ↑[AR] (_1), Ser1808 ↑[AR] (_1)
M0RBD3	Ksr2	kinase suppressor of ras 2	Thr272 ↑[19.47] (_2), Thr276 ↑[6.67] (_2)
Q6P791	Lamtor1	late endosomal/lysosomal adaptor, MAPK and MTOR activator 1	Ser26 ↑[AR] (_1)
G3V6I1	Llgl1	LLGL1, scribble cell polarity complex component	Ser997 ↑[AR] (_3)
D3ZBH5	Lmtk2	lemur tyrosine kinase 2	Ser576 ↑[AR] (_2), Ser704 ↑[AR] (_1), Ser712 ↑[AR] (_2), Ser746 ↑[7.46] (_2), Ser750 ↑[9.64] (_2), Ser756 ↑[AR] (_2), Ser1035 ↑[AR] (_1), Ser1334 ↑[AR] (_3), Ser1524 ↑[AR] (_2), Ser1525 ↑[AR] (_2)
O08873	Madd	MAP-kinase activating death domain	Ser1196 ↓[C] (_1)
P15205	Map1b	microtubule-associated protein 1B	Ser1148 ↑[AR] (_1), Ser1305 ↓[C] (_1), Ser1371 ↓[C] (_1), Thr1781 ↓[C] (_3)
D3Z8I4	Map4k1	mitogen activated protein kinase kinase kinase kinase 1	Ser373 ↓[C] (_2), Ser375 ↓[C] (_2), Tyr379 ↑[4.06] (_2)
A0JN25	Mapt	Microtubule-associated protein tau	Ser306 ↓[C] (_2)
F1LMW7	Marcks	Myristoylated alanine rich protein kinase C substrate	Ser27 ↓[C] (_1)
A0A0G2K7H9	Mark1	microtubule affinity regulating kinase 1	Ser348 ↑[AR] (_2)
D3ZL30	Mast3	microtubule associated serine/threonine kinase 3	Ser354 ↑[12.14] (_1), Ser1201 ↑[AR] (_2), Ser1213 ↑[AR] (_2)
Q63406	Mcf2l	MCF.2 cell line derived transforming sequence-like	Ser964 ↑[AR] (_2)
A0A0G2K382	Mink1	misshapen-like kinase 1	Ser760 ↑[AR] (_1), Ser777 ↑[AR] (_2), Ser781 ↑[AR] (_1)
A0A0G2K2Y8	Mpdz	multiple PDZ domain crumbs cell polarity complex component	Ser1816 ↑[AR] (_2), Ser1818 ↑[AR] (_3), Ser1822 ↑[AR] (_3)
A0A140TA95	Mprip	myosin phosphatase Rho interacting protein	Ser230 ↑[9.91] (_2), Ser294 ↑[AR] (_2)
A0A0G2JX74	Mtor	mechanistic target of rapamycin	Ser2478 ↑[AR] (_2), Ser2481 ↑[AR] (_2)

D4A2D3	Mycbp2	MYC binding protein 2, E3 ubiquitin protein ligase	Ser2644 ↑[19.17] (_2), Ser2646 ↑[19.30] (_2), Ser3791 ↑[AR] (_2), Ser3792 ↑[AR] (_2)
Q4W1H3	Myo9b	myosin IXb	Ser1207 ↑[AR] (_1), Ser1250 ↑[19.19] (_2), Ser1252 ↑[19.19] (_2), Ser1259 ↑[AR] (_2), Ser1267 ↑[AR] (_2), Ser1982 ↓[C] (_1) ↑[25.47] (_2)
A0A0G2JWL3	Nf1	neurofibromin 1	Ser821 ↑[AR] (_2), Ser824 ↑[AR] (_2), Ser2488 ↑[AR] (_1)
Q4G017	Nisch	nischarin	Ser1282 ↑[AR] (_1)
P35465	Pak1	p21 (RAC1) activated kinase 1	Thr184 ↑[AR] (_1), Ser219 ↑[AR] (_2), Ser222 ↑[AR] (_1), Thr228 ↓[C] (_1), Thr229 ↑[AR] (_2)
F1M785	Pdzd2	PDZ domain containing 2	Ser788 ↑[3.54] (_2), Thr793 ↑[3.54] (_2), Ser797 ↑[AR] (_2), Ser887 ↑[AR] (_1), Ser1205 ↑[AR] (_2), Ser1894 ↑[AR] (_2), Ser1898 ↑[AR] (_2)
D3ZXY2	Pdzd8	PDZ domain containing 8	Ser520 ↑[AR] (_1), Ser537 ↑[AR] (_1)
M0R7T1	Phactr4	phosphatase and actin regulator 4	Ser136 ↑[8.38] (_2)
D3ZYT8	Pikfyve	phosphoinositide kinase, FYVE-type zinc finger containing	Ser487 ↑[AR] (_2), Thr489 ↑[AR] (_2)
A0A0G2K6J2	Pkn2	protein kinase N2	Ser468 ↑[22.69] (_1)
F1M2K6	Pkp4	plakophilin 4	Ser220 ↑[12.99] (_2), Ser230 ↑[12.99] (_2), Ser272 ↑[AR] (_2), Ser509 ↑[AR] (_2), Ser511 ↑[AR] (_1)
Q45QJ4	Plcb3	phospholipase C beta 3	Ser537 ↑[AR] (_1)
G3V9D1	Plcd1	phospholipase C, delta 1	Ser454 ↑[AR] (_2), Thr457 ↓[C] (_2), Ser460 ↑[AR] (_2)
Q6J4I0	Ppp1r1b	protein phosphatase 1, regulatory (inhibitor) subunit 1B	Ser102 ↑[11.17] (_1)
P54645	Prkaa1	protein kinase AMP-activated catalytic subunit alpha 1	Thr488 ↑[AR] (_2), Ser527 ↑[AR] (_2) ↓[C] (_3)
A0A0G2K5Q0	Prkcb	protein kinase C, beta	Ser639 ↑[AR] (_1)
D4A0U0	Prkcd	protein kinase C, delta	Ser642 ↑[AR] (_1)
A0A0G2K928	Prkd1	protein kinase D1	Ser161 ↑[24.30] (_2), Ser164 ↑[24.30] (_2), Ser361 ↓[C] (_2), Ser361 ↑[25.40] (_1), Thr364 ↓[C] (_2)
Q5XIS9	Prkd2	protein kinase D2	Ser197 ↑[AR] (_2), Ser198 ↑[AR] (_2), Ser206 ↑[AR] (_2), Ser711 ↑[AR] (_1)
G3V8J5	Psd	pleckstrin and Sec7 domain containing	Ser719 ↑[AR] (_1)
A0A0G2K064	Ptpn6	protein tyrosine phosphatase, non-receptor type 6	Ser10 ↑[AR] (_1)
A0A0G2JYK2	Rab11fip1	RAB11 family interacting protein 1	Ser990 ↓[C] (_2)
P35284	Rab12	RAB12, member RAS oncogene family	Ser20 ↑[AR] (_2), Ser24 ↑[AR] (_2)
G3V9J7	Rabep1	rabaptin, RAB GTPase binding effector protein 1	Ser407 ↑[5.09] (_1), Thr408 ↑[AR] (_2)
Q5EBC7	Rabep2	rabaptin, RAB GTPase binding effector protein 2	Ser176 ↑[13.04] (_2), Ser180 ↑[13.04] (_2)
D3ZX42	Rabgap1	RAB GTPase activating protein 1	Ser988 ↓[C] (_2), Thr992 ↓[C] (_2)
D3ZKH6	Rabgap1l	RAB GTPase activating protein 1-like	Ser128 ↑[24.39] (_2)

Q5FVT1	Ralbp1	ralA binding protein 1	Ser29 ↑[AR] (_2), Ser30 ↑[AR] (_2), Ser48 ↑[190.30] (_2), Ser62 ↑[190.30] (_2)
A0A140TAA3	Ralgapa1	Ral GTPase activating protein catalytic alpha subunit 1	Thr540 ↑[AR] (_1), Ser647 ↑[AR] (_3)
A0A0G2KA57	Ralgapb	Ral GTPase activating protein non-catalytic beta subunit	Thr313 ↑[AR] (_1)
Q0VGK1	Ralgps2	Ral GEF with PH domain and SH3 binding motif 2	Ser293 ↑[AR] (_1), Ser296 ↑[2.12] (_2), Ser308 ↑[2.12] (_2), Ser315 ↑[AR] (_3), Ser316 ↓[C] (_3) ↑[4.92] (_2), Thr326 ↓[C] (_3), Ser329 ↓[C] (_3)
D4A2G9	Ranbp1	RAN binding protein 1	Ser14 ↑[AR] (_3), Thr15 ↑[AR] (_3), Ser21 ↑[AR] (_1)
D4A054	Ranbp2	RAN binding protein 2	Ser1154 ↑[AR] (_1), Ser2511 ↑[62.50] (_1)
M0R920	Ranbp3	RAN binding protein 3	Ser27 ↓[C] (_3), Ser30 ↓[C] (_3), Ser31 ↓[C] (_3), Ser31 ↑[AR] (_2), Ser409 ↑[AR] (_2)
F1LVV3	Ranbp9	RAN binding protein 9	Ser440 ↑[AR] (_3), Ser446 ↑[57.73] (_2), Ser459 ↑[AR] (_1)
F1LV89	Rap1gap	Rap1 GTPase-activating protein	Ser589 ↑[AR] (_1)
D3ZPI4	Rap1gap2	RAP1 GTPase activating protein 2	Ser361 ↓[C] (_2)
F1M8L9	Rapgef1	Rap guanine nucleotide exchange factor 1	Ser375 ↑[AR] (_1)
D3ZTL8	Rapgef6	Rap guanine nucleotide exchange factor 6	Ser1241 ↑[21.40] (_2) ↑[AR], Ser1245 ↑[AR] (_3), Ser1595 ↓[C] (_1)
D4ADX8	Raph1	Ras association (RalGDS/AF-6) and pleckstrin homology domains 1	Ser5 ↑[7.37] (_2), Ser17 ↑[7.37] (_2)
P33568	Rb1	RB transcriptional corepressor 1	Thr363 ↑[AR] (_2), Thr366 ↑[AR] (_2), Ser600 ↑[12.79] (_2), Ser604 ↑[12.79] (_2)
D3ZL11	Rbsn	rabenosyn, RAB effector	Ser216 ↑[AR] (_2), Ser218 ↑[10.22] (_2)
D4AB55	Rgs12	regulator of G-protein signaling 12	Ser104 ↑[AR] (_2), Ser105 ↑[AR] (_2), Ser106 ↑[AR] (_2)
B1H241	Ric8a	RIC8 guanine nucleotide exchange factor A	Tyr434 ↑[2.57] (_2), Ser435 ↑[13.73] (_2), Thr440 ↑[13.73] (_2)
D3ZN37	Rock1	Rho-associated coiled-coil containing protein kinase 1	Ser1105 ↑[8.38] (_2)
F1LQT3	Rock2	Rho-associated coiled-coil containing protein kinase 2	Ser1124 ↑[AR] (_2) ↑[AR] (_1), Ser1127 ↑[6.58] (_2)
D3Z8E0	Rps6ka3	ribosomal protein S6 kinase A3	Ser715 ↑[AR] (_1)
Q0D2L6	Rragc	Ras-related GTP binding C	Ser94 ↑[28.17] (_1)
D3ZI11	Rreb1	ras responsive element binding protein 1	Ser1137 ↑[AR] (_2), Ser1138 ↑[AR] (_2), Ser1177 ↑[AR] (_2), Ser1178 ↑[AR] (_2), Ser1306 ↑[AR] (_1), Ser1361 ↓[C] (_2), Ser1364 ↓[C] (_2), Ser1590 ↑[AR] (_2)
G3V7X2	Scg2	secretogranin II	Ser176 ↑[AR] (_1), Ser495 ↓[C] (_1)
D3ZWS0	Scrib	scribbled planar cell polarity protein	Ser692 ↑[AR] (_1), Ser1204 ↑[AR] (_2), Ser1207 ↑[11.30] (_2), Ser1209 ↑[2.85] (_2)
M0R617	Sh2b1	SH2B adaptor protein 1	Ser126 ↑[AR] (_1) ↑[AR] (_3), Ser127 ↑[AR] (_1) ↑[AR] (_3)
Q9Z200	Sh2b2	SH2B adaptor protein 2	Ser584 ↑[24.73] (_2)
E9PSX8	Sipa1	signal-induced proliferation-associated 1	Ser53 ↑[AR] (_2), Ser65 ↑[AR] (_2)
A0A0G2KAW2	Sipa1l1	signal-induced proliferation-associated 1 like 1	Ser310 ↑[AR] (_1), Thr1530 ↑[AR] (_1), Ser1544 ↑[AR] (_2), Ser1547 ↑[AR] (_2), Ser1618 ↑[AR] (_3) ↑[AR] (_2), Ser1624 ↑[AR] (_2), Ser1626 ↑[AR] (_2), Ser1629 ↑[AR] (_2) ↑[AR] (_3)

F1LYG2	Sipa1l3	signal-induced proliferation-associated 1 like 3	Ser1358 ↑[AR] (_1)
A0A0G2K9N0	Smap2	small ArfGAP2	Ser193 ↑[AR] (_1)
Q63553	Snrk	SNF related kinase	Ser569 ↑[AR] (_1)
B1H267	Snx5	Sorting nexin 5	Ser20 ↓[C] (_1), Ser22 ↓[C] (_1)
P07632	Sod1	superoxide dismutase 1, soluble	Ser99 ↑[AR] (_1)
D4A3T0	Sos1	SOS Ras/Rac guanine nucleotide exchange factor 1	Ser1078 ↓[C] (_2), Ser1082 ↓[C] (_2), Thr1249 ↑[AR] (_2), Ser1251 ↑[AR] (_2), Thr1255 ↑[AR] (_2), Ser1318 ↑[AR] (_1), Ser1319 ↑[AR] (_1)
D3ZEX7	Spire1	Spire-type actin nucleation factor 1	Ser399 ↓[C] (_3), Ser400 ↓[C] (_3), Ser402 ↓[C] (_3)
Q9WUD9	Src	SRC proto-oncogene, non-receptor tyrosine kinase	Ser75 ↑[10.91] (_1)
D4A208	Srgap2	SLIT-ROBO Rho GTPase activating protein 2	Ser994 ↑[6.28] (_2)
E9PTN4	Srpk1	SRSF protein kinase 1	Ser51 ↑[3.40] (_2), Ser309 ↑[AR] (_2), Ser311 ↑[AR] (_2)
A0A0G2JX62	Srpk2	SRSF protein kinase 2	Ser487 ↑[AR] (_2), Thr491 ↑[AR] (_2)
B1WBQ5	Stk3	serine/threonine kinase 3	Ser316 ↑[29.15] (_1)
A0A096MK73	Stmn1	stathmin 1	Ser25 ↑[12.28] (_1), Ser38 ↑[23.17] (_1), Ser46 ↑[AR] (_1)
A0A0G2K8P5	Stmn3	stathmin 3	Ser50 ↑[AR] (_2), Ser53 ↑[AR] (_2)
A0A0G2K6Y9	Stx7	Syntaxin 7	Ser196 ↑[7.69] (_2), Ser203 ↑[7.69] (_2)
Q9WU70	Stxbp5	syntaxin binding protein 5	Ser724 ↑[AR] (_2)
D3Z881	Tbc1d4	TBC1 domain family, member 4	Ser597 ↑[AR] (_2), Ser600 ↑[AR] (_2)
F1LWZ7	Tbc1d8	TBC1 domain family, member 8	Ser464 ↑[AR] (_1)
Q587K3	Tbc1d10a	TBC1 domain family, member 10a	Ser45 ↑[5.73] (_2)
D3ZSY8	Tbc1d10b	TBC1 domain family, member 10b	Ser234 ↑[AR] (_2), Ser644 ↑[AR] (_2)
B1WBS1	Tbc1d25	TBC1 domain family, member 25	Ser506 ↑[AR] (_1)
D3ZWV8	Tiam1	T-cell lymphoma invasion and metastasis 1	Ser725 ↑[AR] (_1), Ser1462 ↑[AR] (_2)
F1LPP2	Tlk2	tousled-like kinase 2	Ser749 ↑[AR] (_1)
D3ZZQ0	Tnik	TRAF2 and NCK interacting kinase	Ser640 ↑[15.17] (_1), Ser678 ↑[AR] (_2), Ser680 ↑[AR] (_2), Ser769 ↑[AR] (_1)
O08629	Trim28	tripartite motif-containing 28	Ser27 ↑[AR] (_2), Ser52 ↑[AR] (_1), Ser502 ↑[AR] (_1), Ser595 ↑[AR] (_2) ↓[-46.22] (_3), Ser597 ↓[-38.19] (_3)
A0A1P0PBZ6	Trio	trio Rho guanine nucleotide exchange factor	Ser2408 ↑[AR] (_2), Ser2412 ↑[AR] (_2)
Q9Z136	Tsc1	tuberous sclerosis 1	Ser561 ↑[AR] (_2), Ser565 ↑[AR] (_2)
D3ZLW4	Tsc2	tuberous sclerosis 2	Ser1389 ↑[3.55] (_2)
A0A0G2K3A0	Wnk1	WNK lysine deficient protein kinase 1	Ser2154 ↑[AR] (_1)
D3ZMJ7	Wnk2	WNK lysine deficient protein kinase 2	Ser45 ↑[AR] (_1), Ser1774 ↑[AR] (_1), Ser1830 ↑[16.53] (_2), Ser1831 ↑[16.53] (_2)
A0A1W2Q6C5	Wnk3	WNK lysine deficient protein kinase 3	Ser436 ↑[AR] (_3), Thr449 ↑[AR] (_3)

↑, elevated phosphorylation; ↓, decreased phosphorylation; ↓[C], detected only in control (negative siRNA-treated) cells; ↑[AR], detected only in β-arrestin2-deficient cells; (_1, _2, _3), multiplicity

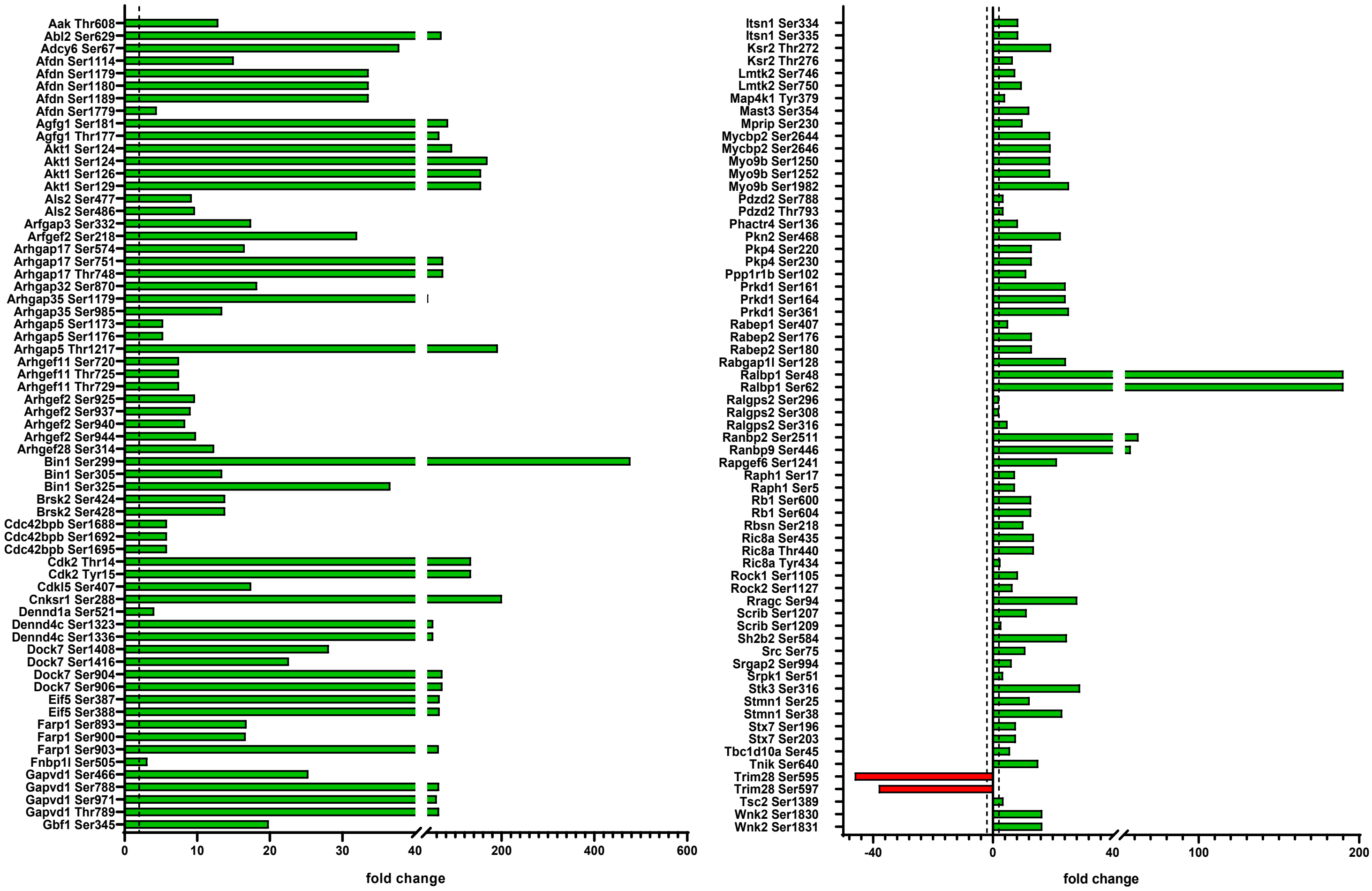

Figure S2. Quantitative changes in phosphoproteins involved in the regulation of small GTPase activity in GH1 cells after siRNA-mediated β-arrestin2 knockdown

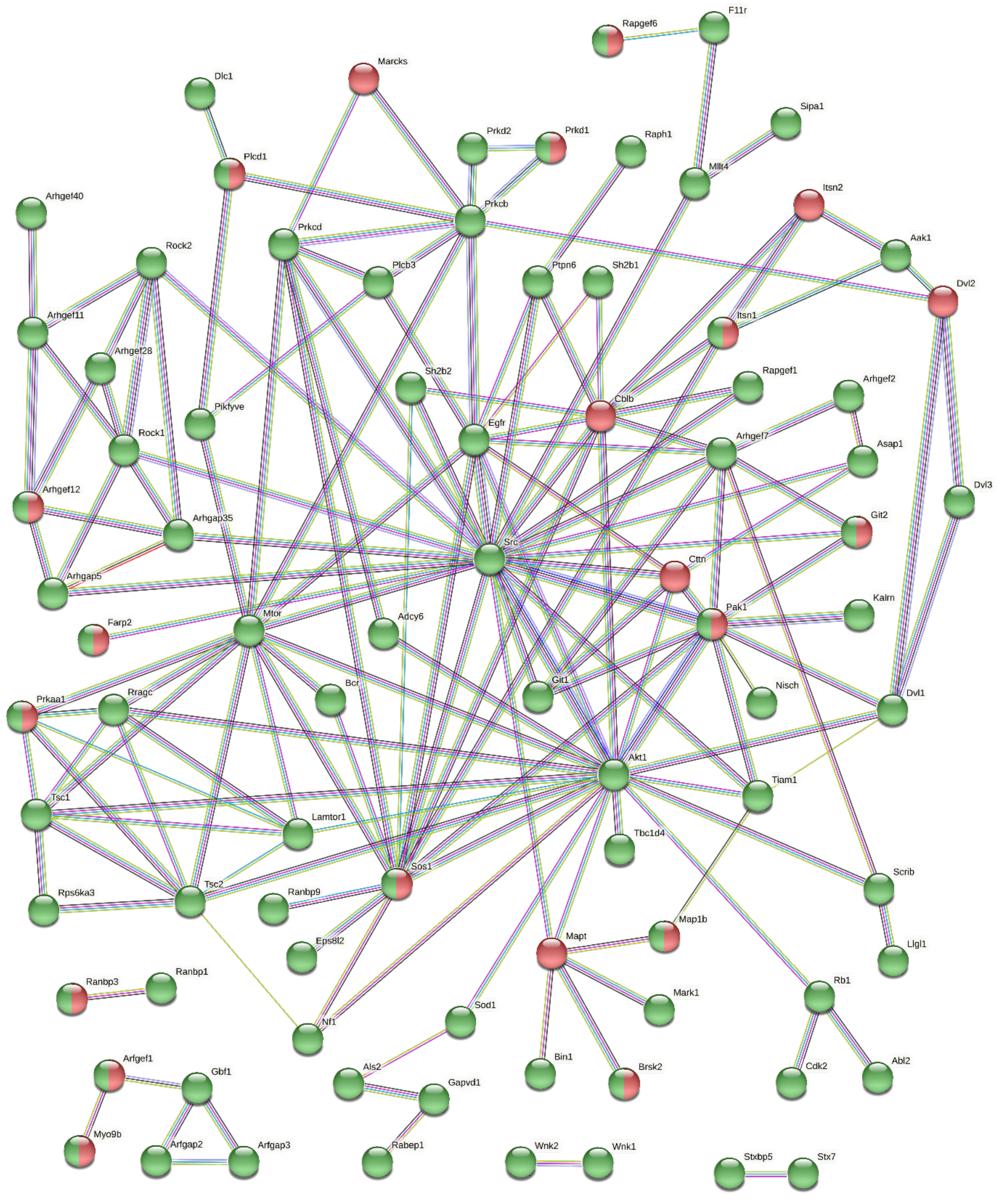

Figure S3. A network of differentially phosphorylated proteins involved in the regulation of small GTPase activity in GH1 cells after siRNA-mediated βarrestin-2 knockdown. Elevated phosphorylation in green, decreased phosphorylation in red.

Table S2. A list of differentially phosphorylated proteins involved in the regulation of small GTPase activity in GH1 cells after stimulation with 1 μM TRH

Uniprot ID	Gene ID	Gene name	Phosphorylated regions
F1LRI7	Aak1	AP2 associated kinase 1	Ser626 ↑[TRH] (_2)
F1M9N7	Agfg1	ArfGAP with FG repeats 1	Thr177 ↓[C] (_2), Ser181 ↓[C] (_2)
P47196	Akt1	AKT serine/threonine kinase 1	Ser126 ↑[TRH] (_2) ↓[C] (_1)
A0A0G2JWD6	Ap3b1	adaptor-related protein complex 3, beta 1 subunit	Ser276 ↓[C] (_1)
D4A631	Arfgef1	ADP ribosylation factor guanine nucleotide exchange factor 1	Ser394 ↓[C] (_2)
Q7TSU1	Arfgef2	ADP ribosylation factor guanine nucleotide exchange factor 2	Ser227 ↓[C] (_1)
A0A0G2JYA2	Arfip1	ADP-ribosylation factor interacting protein 1	Ser5 ↓[C] (_1)
D4AAV4	Arhgap17	Rho GTPase activating protein 17	Ser574 ↓[C] (_1), Thr742 ↓[C] (_3), Thr746 ↓[C] (_3), Thr748 ↓[C] (_3), Ser751 ↓[C] (_3)
D4AD82	Arhgap35	Rho GTPase activating protein 35	Ser975 ↓[C] (_1), Ser1174 ↓[C] (_2), Ser1179 ↓[C] (_2),
A0A1B0GWY5	Arhgef 2	Rho/Rac guanine nucleotide exchange factor 2	Ser937 ↓[C] (_2), Ser940 ↓[C] (_2)
A0A0G2JZC6	Arhgef11	Rho guanine nucleotide exchange factor 11	Ser308 ↓[C] (_3)
P0C6P5	Arhgef28	Rho guanine nucleotide exchange factor 28	Ser314 ↓[C] (_1), Thr1197 ↓[C] (_2), Ser1198 ↓[C] (_2), Ser1200 ↓[C] (_2)
Q4V8I5	Arl6ip4	ADP ribosylation factor like GTPase 6 interacting protein 4	Ser142 ↓[C] (_1)
F1LMX1	Bin1	bridging integrator 1	Ser297 ↓[-25.16] (_1), Ser325 ↓[C] (_2), Ser333 ↓[C] (_2) ↓[C] (_1)
D3ZML2	Brsk2	BR serine/threonine kinase 2	Ser424 ↓[C] (_2), Ser428 ↓[C] (_2) ↓[C] (_3), Ser436 ↓[C] (_3)
Q8K4S7	Cblb	Cbl proto-oncogene B	Ser476 ↓[C] (_3), Ser480 ↓[C] (_3), Ser483 ↓[C] (_3), Ser484 ↓[C] (_2)
Q7TT49	Cdc42bpb	CDC42 binding protein kinase beta	Ser1692 ↓[C] (_1)
A0A1B0GWS4	Cttn	cortactin	Tyr139 ↓[C] (_2)
A0A0G2KB92	Dclk1	doublecortin-like kinase 1	Ser363 ↓[C] (_2), Ser364 ↓[C] (_2), Ser364 ↓[C] (_1)
F1LTD7	Dennd4c	DENN domain containing 4C	Ser1310 ↓[C] (_2)
F1LRS2	Dock7	dedicator of cytokinesis 7	Ser904 ↓[C] (_2), Ser906 ↓[C] (_2), Ser918 ↓[C] (_2)
D4ADV8	Dvl3	dishevelled segment polarity protein 3	Ser125 ↓[C] (_1)
D3ZFK8	Farp2	FERM, ARH/RhoGEF and pleckstrin domain protein 2	Ser474 ↓[C] (_3), Ser477 ↓[C] (_2) ↓[C] (_3)
Q2HWF0	Fnbp1l	formin binding protein 1-like	Ser501 ↓[C] (_2) ↓[C] (_1), Ser505 ↓[-5.52] (_2)
D4A022	Gapvd1	GTPase activating protein and VPS9 domains 1	Ser788 ↓[C] (_2)
A0A0G2K3N1	Gbf1	golgi brefeldin A resistant guanine nucleotide exchange factor 1	Ser340 ↑[TRH] (_2)
A0A0G2K527	Git1	GIT ArfGAP 1	Ser379 ↓[C] (_3), Thr383 ↓[C] (_3)
Q66H91	Git2	GIT ArfGAP 2	Ser394 ↓[C] (_2) ↓[-5.24] (_3)
Q9WVE9	Itsn1	intersectin 1	Ser894 ↓[C] (_1) ↓[C] (_2)
D3ZM20	Kif13a	kinesin family member 13A	Ser510 ↓[C] (_3), Ser511 ↓[C] (_3)
A0A1B0GWT8	Klc1	kinesin light chain 1	Ser7 ↓[C] (_2)

O08873	Madd	MAP-kinase activating death domain	Ser828 ↓[C] (_1)
P15205	Map1b	microtubule-associated protein 1B	Ser1305 ↓[C] (_1), Ser1368 ↓[C] (_2), Ser1369 ↓[C] (_2), Ser1371 ↓[C] (_1), Ser1772 ↓[C] (_3), Ser1775 ↓[C] (_3), Thr1781 ↓[C] (_3)
A0A0G2K3R1	Map3k4	mitogen activated protein kinase kinase kinase 4	Ser59 ↓[C] (_3) ↓[C] (_2), Ser77 ↓[C] (_3) ↓[C] (_2)
D3Z8I4	Map4k1	mitogen activated protein kinase kinase kinase kinase 1	Ser370 ↓[C] (_2), Ser373 ↓[C] (_2), Ser375 ↓[C] (_2), Tyr379 ↓[C] (_2)
A0JN25	Mapt	microtubule-associated protein tau	Ser306 ↓[-38.03] (_2)
F1LMW7	Marcks	myristoylated alanine rich protein kinase C substrate	Ser27 ↓[C] (_1)
Q4W1H3	Myo9b	myosin IXb	Ser1982 ↓[C] (_1)
P35465	Pak1	p21 (RAC1) activated kinase 1	Ser174 ↓[-5.65] (_1), Ser219 ↓[C] (_1), Thr228 ↓[C] (_1)
F1M785	Pdzd2	PDZ domain containing 2	Ser788 ↓[-7.58] (_2), Thr793 ↓[-7.58] (_2), Ser2157 ↓[C] (_2), Ser2159 ↓[C] (_2)
G3V9D1	Plcd1	phospholipase C, delta 1	Ser460 ↑[TRH] (_2)
D3ZA21	Plekhg3	pleckstrin homology and RhoGEF domain containing G3	Ser636 ↓[C] (_2)
P54645	Prkaa1	protein kinase AMP-activated catalytic subunit alpha 1	Ser486 ↓[-13.17] (_2)
A0A0G2K928	Prkd1	protein kinase D1	Ser189 ↓[C] (_2), Ser192 ↓[C] (_2), Ser361 ↓[C] (_1), Ser361 ↓[C] (_2), Thr364 ↓[C] (_2)
P70600	Ptk2b	protein tyrosine kinase 2 beta	Ser389 ↓[C] (_3), Ser392 ↓[C] (_3), Ser394 ↓[C] (_3), Ser396 ↓[C] (_3), Ser399 ↓[C] (_3)
A0A0G2K1B4	Rab3ip	RAB3A interacting protein	Ser272 ↓[C] (_1)
A0A0G2JYK2	Rab11fip1	RAB11 family interacting protein 1	Ser990 ↓[C] (_2)
A0A0G2K1W1	Rab11fip5	RAB11 family interacting protein 5	Ser1058 ↓[C] (_2)
D3ZX42	Rabgap1	RAB GTPase activating protein 1	Ser988 ↓[C] (_2), Thr992 ↓[C] (_2)
D3ZKH6	Rabgap1l	RAB GTPase activating protein 1-like	Ser128 ↓[C] (_1)
D4A1Z8	Radil	Rap associating with DIL domain	Ser206 ↓[C] (_1)
Q0VGK1	Ralgps2	Ral GEF with PH domain and SH3 binding motif 2	Ser316 ↓[-6.27] (_2)
D4A054	Ranbp2	RAN binding protein 2	Ser2088 ↑[TRH] (_3), Ser2092 ↑[TRH] (_3), Ser2096 ↑[TRH] (_3), Ser2097 ↑[TRH] (_3)
D3Z7Z5	Ranbp10	RAN binding protein 10	Ser463 ↓[C] (_2), Ser467 ↓[C] (_2) ↓[C] (_3)
F1MAA5	Rangap1	RAN GTPase activating protein 1	Ser427 ↑[TRH] (_2)
D3ZPI4	Rap1gap2	RAP1 GTPase activating protein 2	Ser361 ↓[C] (_2), Ser365 ↓[C] (_2)
D3ZTL8	Rapgef6	Rap guanine nucleotide exchange factor 6	Ser1595 ↓[C] (_1)
D4ADX8	Raph1	Ras association (RalGDS/AF-6) and pleckstrin homology domains 1	Ser5 ↓[C] (_2), Ser17 ↓[C] (_2)
B1H241	Ric8a	RIC8 guanine nucleotide exchange factor A	Tyr434 ↓[C] (_2), Ser435 ↓[C] (_2), Thr440 ↓[C] (_2)
A0A096P6M3	Rims2	regulating synaptic membrane exocytosis 2	Ser1143 ↓[C] (_2), Ser1485 ↓[C] (_2), Ser1486 ↓[C] (_2)
D3ZI11	Rreb1	ras responsive element binding protein 1	Ser1590 ↓[C] (_3), Ser1603 ↓[C] (_3)

G3V7X2	Scg2	secretogranin II	Ser491 ↑[TRH] (_1), Ser494 ↑[TRH] (_1), Ser495 ↓[-3.50] (_1)
A0A0G2KAW2	Sipa1l1	signal-induced proliferation-associated 1 like 1	Ser1629 ↑[TRH] (_3), Ser1690 ↓[C] (_2)
B1H267	Snx5	sorting nexin 5	Ser20 ↓[C] (_1), Ser22 ↓[C] (_1)
E9PTN4	Srpk1	SRSF protein kinase 1	Ser33 ↓[C] (_3), Ser37 ↓[C] (_3), Ser39 ↓[C] (_3), Ser51 ↓[C] (_3) ↓[C] (_2), Thr453 ↓[C] (_2), Ser455 ↓[C] (_2)
G3V8I4	Stx4	syntaxin 4	Ser15 ↓[-28.99] (_1)
A0A0G2K6Y9	Stx7	syntaxin 7	Ser196 ↓[C] (_2), Ser203 ↓[C] (_2)
F1LRL4	Tbc1d9b	TBC1 domain family, member 9B	Ser1084 ↓[C] (_1)
Q587K3	Tbc1d10a	TBC1 domain family, member 10a	Ser45 ↓[C] (_2)
D3ZSY8	Tbc1d10b	TBC1 domain family, member 10b	Ser231 ↑[TRH] (_2), Ser234 ↑[TRH] (_2)
D3ZLW4	Tsc2	tuberous sclerosis 2	Ser1389 ↓[C] (_2)
A0A0G2K3A0	Wnk1	WNK lysine deficient protein kinase 1	Ser1809 ↓[C] (_2)
D3ZMJ7	Wnk2	WNK lysine deficient protein kinase 2	Ser45 ↑[TRH] (_1), Ser49 ↓[C] (_1), Ser1830 ↓[C] (_2), Ser1831 ↓[C] (_2)

↑, elevated phosphorylation; ↓, decreased phosphorylation; ↓[C], detected only in control (negative siRNA-treated) cells; ↑[TRH], detected only in cells stimulated with TRH; (_1, _2, _3), multiplicity

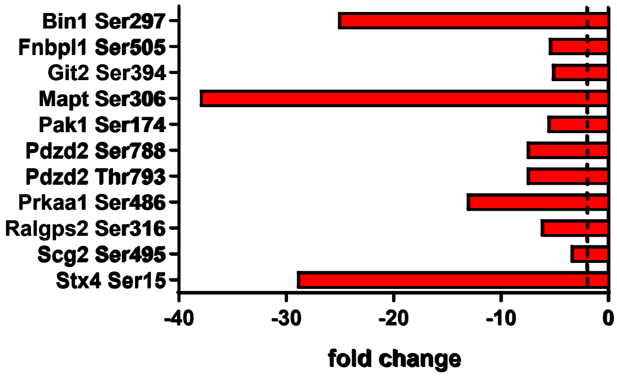

Figure S4. Quantitative changes in phosphoproteins involved in the regulation of small GTPase activity in GH1 cells after stimulation with 1 µM TRH

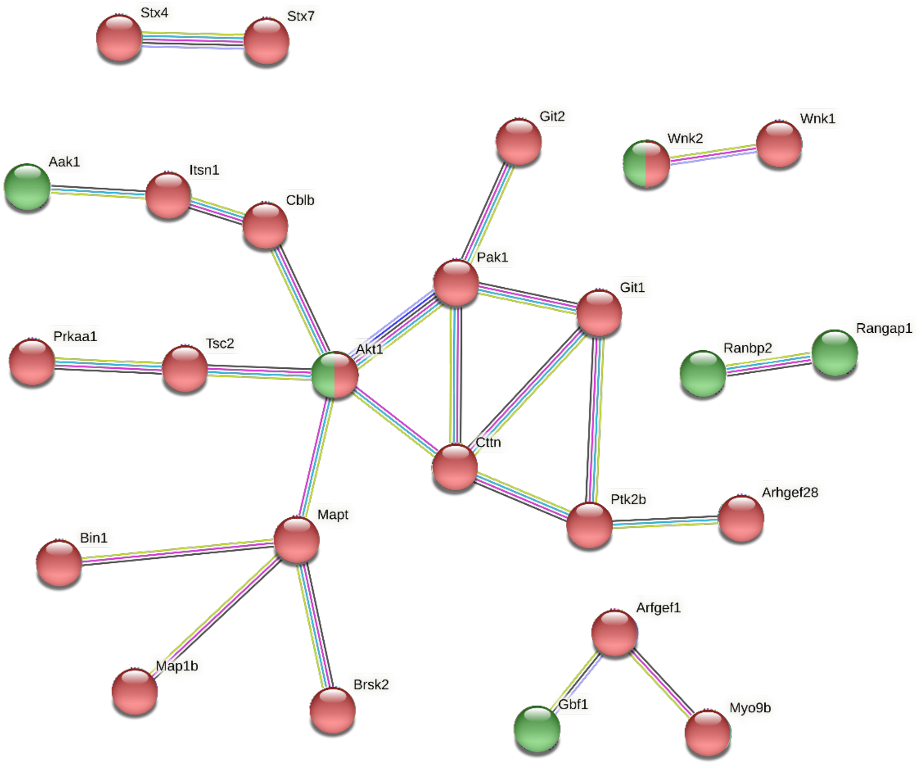

Figure S5. A network of differentially phosphorylated proteins involved in the regulation of small GTPase activity in GH1 cells after TRH treatment. Elevated phosphorylation in green, decreased phosphorylation in red.

Table S3. A list of differentially phosphorylated proteins related to regulation of small GTPase activity in GH1 cells after stimulation with 1 μM TAL

Uniprot ID	Gene ID	Gene name	Phosphorylated regions
O35889	Afdn	afadin, adherens junction formation factor	Ser1779 ↓[-3.44] (_1)
F1M9N7	Agfg1	ArfGAP with FG repeats 1	Thr177 ↓[-5.60] (_2), Ser181 ↓[-4.36] (_2)
P47196	Akt1	AKT serine/threonine kinase 1	Ser124 ↓[-5.22] (_1), Ser126 ↓[-363.47] (_1)
A0A0G2JWD6	Ap3v1	adaptor-related protein complex 3, beta 1 subunit	Ser276 ↓[C] (_1)
D4A631	Arfgef1	ADP ribosylation factor guanine nucleotide exchange factor 1	Ser286 ↓[C] (_1), Ser394 ↓[C] (_2)
Q7TSU1	Arfgef2	ADP ribosylation factor guanine nucleotide exchange factor 2	Ser218 ↓[-9.87] (_2), Ser227 ↓[C] (_1)
A0A0G2JYA2	Arfip1	ADP-ribosylation factor interacting protein 1	Ser5 ↓[C] (_1)
Q6TUE6	Arhgap5	Rho GTPase activating protein 5	Ser1173 ↓[-9.69] (_2), Ser1176 ↓[-10.49] (_2)
D4AAV4	Arhgap17	Rho GTPase activating protein 17	Ser574 ↓[-3.23] (_1), Thr748 ↓[-6.45] (_2), Ser751 ↓[-6.45] (_2), Thr752 ↓[-6.45] (_2)
D4AD82	Arhgap35	Rho GTPase activating protein 35	Ser975 ↓[C] (_1), Ser1174 ↓[C] (_2), Ser1179 ↓[C] (_2)
A0A1B0GWY5	Arhgef2	Rho/Rac guanine nucleotide exchange factor 2	Ser925 ↓[C] (_2), Ser937 ↓[-8.00] (_2), Ser940 ↓[-6.21] (_2)
A0A0G2JZC6	Arhgef11	Rho guanine nucleotide exchange factor 11	Ser308 ↓[C] (_3)
D3ZYR0	Arhgef12	Rho guanine nucleotide exchange factor 12	Ser341 ↓[-4.73] (_1)
P0C6P5	Arhgef28	Rho guanine nucleotide exchange factor 28	Ser314 ↓[C] (_1), Thr1197 ↓[C] (_2), Ser1198 ↓[C] (_2), Ser1200 ↓[C] (_2)
Q4V8I5	Arl6ip4	ADP-ribosylation factor like GTPase 6 interacting protein 4	Ser142 ↓[C] (_1)
A0A0G2K451	Asap1	ArfGAP with SH3 domain, ankyrin repeat and PH domain 1	Ser647 ↓[-6.25] (_2), Ser651 ↓[-6.25] (_2)
A0A0G2K808	Asap2	ArfGAP with SH3 domain, ankyrin repeat and PH domain 2	Ser653 ↓[-5.78] (_1)
F1LMX1	Bin1	bridging integrator 1	Ser297 ↓[-7.05] (_1), Ser305 ↓[-5.74] (_2), Ser325 ↓[-8.53] (_2), Ser333 ↓[-8.53] (_2)
D3ZML2	Brsk2	BR serine/threonine kinase 2	Ser424 ↓[C] (_2), Ser428 ↓[C] (_3) ↓[C] (_2), Ser436 ↓[C] (_3)
Q8K4S7	Cblb	Cbl proto-oncogene B	Ser476 ↓[C] (_3), Ser480 ↓[C] (_3), Ser483 ↓[C] (_3), Ser484 ↓[-11.87] (_2)
Q7TT49	Cdc42bpb	CDC42 binding protein kinase beta	Ser1692 ↓[-31.75] (_1), Ser1695 ↓[-17.53] (_2)
A0A1B0GWS4	Cttn	cortactin	Tyr139 ↓[C] (_2)
A0A0G2KB92	Dclk1	doublecortin-like kinase 1	Ser330 ↓[-12.50] (_2), Ser334 ↓[C] (_3), Ser337 ↓[C] (_2), Ser340 ↓[C] (_3), Ser363 ↓[C] (_2), Ser364 ↓[C] (_2)
F1M241	Dennd1a	DENN domain containing 1A	Ser521 ↓[C] (_2)
F1LTD7	Dennd4c	DENN domain containing 4C	Ser1310 ↓[C] (_2), Ser1323 ↓[-5.85] (_2), Ser1336 ↓[-5.85] (_2)
A0A0G2KAH4	Dock6	dedicator of cytokinesis 6	Ser1260 ↑[TAL] (_1)
F1LRS2	Dock7	dedicator of cytokinesis 7	Ser904 ↓[-5.08] (_2), Ser906 ↓[-5.08] (_2)
D3ZB71	Dvl2	dishevelled segment polarity protein 2	Ser211 ↓[-8.27] (_1)

D4ADV8	Dvl3	dishevelled segment polarity protein 3	Ser125 ↓[-3.22] (_1)
Q64350	Eif2b5	eukaryotic translation initiation factor 2B subunit 5 epsilon	Ser539 ↓[C] (_1)
Q07205	Eif5	eukaryotic translation initiation factor 5	Ser387 ↓[-5.56] (_2), Ser388 ↓[-5.56] (_2)
M0R4L1	Erbb2	erb-b2 receptor tyrosine kinase 2	Thr152 ↓[-5.36] (_2), Ser154 ↓[-5.36] (_2)
F1LYQ8	Farp1	FERM, ARH/RhoGEF and pleckstrin domain protein 1	Ser893 ↓[C] (_2), Thr902 ↓[-14.79] (_2) ↓[-6.76] (_3)
D3ZFK8	Farp2	FERM, ARH/RhoGEF and pleckstrin domain protein 2	Ser474 ↓[C] (_3), Ser477 ↓[C] (_3) ↓[C] (_2)
Q2HWF0	Fnbp1l	formin binding protein 1-like	Ser501 ↓[C] (_2) ↓[C] (_1), Ser505 ↓[C] (_2)
A0A0G2K3N1	Gbf1	golgi brefeldin A resistant guanine nucleotide exchange factor 1	Ser345 ↓[-4.26] (_2), Ser1291 ↓[C] (_1)
A0A0G2K527	Git1	GIT ArfGAP 1	Ser379 ↑[TAL] (_2)
Q66H91	Git2	GIT ArfGAP 2	Ser394 ↓[-2.92] (_2), Ser397 ↓[-3.83] (_1)
A0A0G2JSH4	Gsk3b	glycogen synthase kinase 3 beta	Ser389 ↓[-3.03] (_1)
D2XV59	Gtpbp1	GTP binding protein 1	Ser8 ↓[-4.55] (_2), Ser12 ↓[-2.06] (_3), Ser24 ↓[-2.50] (_3), Ser25 ↓[-4.55] (_2), Ser580 ↓[-61.26] (_1)
Q9WVE9	Itsn1	intersectin 1	Ser894 ↓[C] (_1)
M0R7A6	Itsn2	intersectin 2	Ser908 ↓[-2.95] (_1)
A0A1B0GWT8	Klc1	kinesin light chain 1	Ser7 ↓[C] (_2)
M0RBD3	Ksr2	kinase suppressor of ras 2	Thr276 ↓[-3.07] (_2)
G3V6I1	Llgl1	LLGL1, scribble cell polarity complex component	Ser997 ↓[C] (_2)
D3ZBH5	Lmtk2	lemur tyrosine kinase 2	Ser496 ↓[C] (_2), Ser580 ↓[C] (_2), Ser1334 ↑[TAL] (_3)
O08873	Madd	MAP-kinase activating death domain	Ser828 ↓[C] (_1)
P15205	Map1b	microtubule-associated protein 1B	Ser1305 ↓[C] (_1), Ser1368 ↓[C] (_2), Ser1369 ↓[C] (_2), Ser1371 ↓[-4.48] (_1), Ser1772 ↓[C] (_3), Ser1775 ↓[C] (_3)
A0A0G2K3R1	Map3k4	mitogen activated protein kinase kinase kinase 4	Ser59 ↓[C] (_3), Ser59 ↓[-11.89] (_2), Ser77 ↓[-11.89] (_2), Ser77 ↓[C] (_3)
Q810W7	Mast1	microtubule associated serine/threonine kinase 1	Ser346 ↓[-2.01] (_1)
D3ZL30	Mast3	microtubule associated serine/threonine kinase 3	Ser354 ↓[C] (_1)
Q4W1H3	Myo9b	myosin IXb	Ser1250 ↓[-5.20] (_2), Ser1252 ↓[-5.20] (_2), Ser1982 ↓[C] (_1)
P35465	Pak1	p21 (RAC1) activated kinase 1	Ser219 ↓[-2.29] (_1)
F1M785	Pdzd2	PDZ domain containing 2	Ser788 ↓[C] (_2), Thr793 ↓[C] (_2), Ser2157 ↓[C] (_2), Ser2159 ↓[C] (_2)
D3ZXY2	Pdzd8	PDZ domain containing 8	Ser495 ↓[-6.04] (_1), Thr972 ↓[-13.07] (_2), Ser978 ↓[-13.07] (_2)
D3ZYT8	Pikfyve	phosphoinositide kinase, FYVE-type zinc finger containing	Ser341 ↓[-4.02] (_1), Ser487 ↓[-5.75] (_1), Thr489 ↓[-9.17] (_1)
F1M2K6	Pkp4	plakophilin 4	Ser220 ↓[-7.30] (_2), Ser230 ↓[-7.30] (_2)
G3V9D1	Plcd1	phospholipase C, delta 1	Ser454 ↑[TAL] (_2), Ser460 ↑[TAL] (_2)
A0A0G2K4N6	Plcl1	phospholipase C-like 1	Thr458 ↓[C] (_1)
D3ZA21	Plekhg3	pleckstrin homology and RhoGEF domain containing G3	Ser636 ↓[C] (_2)
Q6J4I0	Ppp1r1b	protein phosphatase 1, regulatory (inhibitor) subunit 1B	Ser102 ↓[-4.19] (_1)

P54645	Prkaa1	protein kinase AMP-activated catalytic subunit alpha 1	Ser486 ↓[-6.03] (_2)
A0A0G2K928	Prkd1	protein kinase D1	Ser161 ↓[-4.59] (_2), Ser164 ↓[-4.59] (_2), Ser189 ↓[C] (_2), Ser192 ↓[C] (_2), Ser361 ↓[C] (_1)
D4A404	Psd3	pleckstrin and Sec7 domain containing 3	Ser490 ↓[-6.45] (_2)
P70600	Ptk2b	protein tyrosine kinase 2 beta	Ser389 ↓[C] (_3), Ser392 ↓[C] (_3), Ser394 ↓[C] (_3), Ser396 ↓[C] (_3), Ser399 ↓[C] (_3)
A0A0G2K1B4	Rab3ip	RAB3A interacting protein	Ser272 ↓[C] (_1)
A0A0G2JYK2	Rab11fip1	RAB11 family interacting protein 1	Ser990 ↓[C] (_2)
A0A0G2K1W1	Rab11fip5	RAB11 family interacting protein 5	Ser825 ↓[-14.04] (_1), Ser1058 ↓[C] (_2)
G3V9J7	Rabep1	rabaptin, RAB GTPase binding effector protein 1	Ser407 ↓[C] (_1)
Q5EBC7	Rabep2	rabaptin, RAB GTPase binding effector protein 2	Ser176 ↓[-3.38] (_2), Ser180 ↓[-3.38] (_2)
D3ZX42	Rabgap1	RAB GTPase activating protein 1	Ser988 ↓[-7.56] (_2), Thr992 ↓[-7.56] (_2)
D3ZKH6	Rabgap1l	RAB GTPase activating protein 1-like	Ser128 ↓[-10.66] (_1) ↓[-5.17] (_2)
D4A1Z8	Radil	Rap associating with DIL domain	Ser206 ↓[C] (_1)
Q5FVT1	Ralbp1	ralA binding protein 1	Ser48 ↓[-2.59] (_2), Ser62 ↓[-2.59] (_2)
D3ZKI6	Ralgapa2	Ral GTPase activating protein catalytic alpha subunit 2	Ser817 ↓[C] (_2), Ser818 ↓[C] (_2)
Q0VGK1	Ralgps2	Ral GEF with PH domain and SH3 binding motif 2	Ser296 ↓[-2.97] (_2), Ser308 ↓[-2.97] (_2), Ser316 ↓[-3.60] (_2)
D4A054	Ranbp2	RAN binding protein 2	Ser2092 ↓[C] (_2), Ser2097 ↓[C] (_2)
D3Z7Z5	Ranbp10	RAN binding protein 10	Ser467 ↓[-45.41] (_3)
F1M8L9	Rapgef1	Rap guanine nucleotide exchange factor 1	Ser239 ↓[C] (_2)
D4ADX8	Raph1	Ras association (RalGDS/AF-6) and pleckstrin homology domains 1	Ser5 ↓[C] (_2), Ser17 ↓[C] (_2)
P33568	Rb1	RB transcriptional corepressor 1	Ser30 ↓[-3.97] (_1)
D3ZL11	Rbsn	rabenosyn, RAB effector	Ser216 ↑[TAL] (_2), Ser218 ↓[-23.64] (_2)
B1H241	Ric8a	RIC8 guanine nucleotide exchange factor A	Tyr434 ↓[-7.35] (_2), Ser435 ↓[-13.23] (_2), Thr440 ↓[-13.23] (_2)
A0A096P6M3	Rims2	regulating synaptic membrane exocytosis 2	Ser1485 ↓[-6.98] (_2), Ser1486 ↓[-6.36] (_2)
D3ZI11	Rreb1	ras responsive element binding protein 1	Ser1590 ↑[TAL] (_2) ↓[C] (_3), Ser1603 ↓[C] (_3)
G3V7X2	Scg2	secretogranin II	Ser495 ↓[-4.13] (_1)
D3ZWS0	Scrib	scribbled planar cell polarity protein	Ser1483 ↓[C] (_1)
B2RZD1	Sec61b	sec61 translocon beta subunit	Ser14 ↑[TAL] (_1) ↓[C] (_2), Ser17 ↓[C] (_1)
D4A3T0	Sos1	SOS Ras/Rac guanine nucleotide exchange factor 1	Ser1078 ↓[-5.61] (_2), Ser1082 ↓[-5.61] (_2)
D3ZEX7	Spire1	spire-type actin nucleation factor 1	Ser399 ↓[-2.19] (_3), Ser400 ↓[-2.19] (_3), Ser402 ↓[-2.19] (_3)
D4A208	Srgap2	SLIT-ROBO Rho GTPase activating protein 2	Ser990 ↓[C] (_2)
E9PTN4	Srpk1	SRSF protein kinase 1	Ser33 ↓[-10.08] (_3), Ser37 ↓[-10.08] (_3), Ser39 ↓[-7.44] (_3), Ser51 ↓[-4.50] (_2) ↓[-10.08] (_3), Thr453 ↓[C] (_2), Ser455 ↓[C] (_2)
B1WBQ5	Stk3	serine/threonine kinase 3	Ser316 ↓[-13.41] (_1)
G3V8I4	Stx4	syntaxin 4	Ser15 ↓[-17.77] (_1)
A0A0G2K6Y9	Stx7	syntaxin 7	Ser196 ↓[C] (_2), Ser203 ↓[C] (_2)

F1LRL4	Tbc1d9b	TBC1 domain family member 9B	Ser1084 ↓[C] (_1)
Q587K3	Tbc1d10a	TBC1 domain family, member 10a	Ser45 ↓[C] (_2)
D3ZSY8	Tbc1d10b	TBC1 domain family, member 10b	Ser128 ↓[C] (_3), Thr135 ↓[C] (_3), Ser231 ↑[TAL] (_2), Ser234 ↑[TAL] (_2)
D3ZZQ0	Tnik	TRAF2 and NCK interacting kinase	Ser640 ↓[-10.91] (_1), Ser764 ↓[-5.33] (_2), Ser769 ↓[-5.33] (_2)
F1LN91	Tns3	tensin 3	Ser891 ↓[-5.14] (_1)
O08629	Trim28	tripartite motif-containing 28	Ser31 ↓[-5.71] (_1), Ser474 ↓[C] (_1), Ser595 ↓[-53.08] (_3), Ser597 ↓[-28.00] (_3)
D3ZMG0	Ulk1	unc-51 like autophagy activating kinase 1	Ser450 ↓[-5.76] (_1)
A0A0G2K9N2	Vav2	vav guanine nucleotide exchange factor 2	Ser703 ↓[C] (_2), Ser704 ↓[C] (_2), Ser706 ↓[C] (_2)
A0A0G2K3A0	Wnk1	WNK lysine deficient protein kinase 1	Ser1809 ↓[C] (_2)
D3ZMJ7	Wnk2	WNK lysine deficient protein kinase 2	Ser49 ↓[C] (_1)

↑, elevated phosphorylation; ↓, decreased phosphorylation; ↓[C], detected only in control (negative siRNA-treated) cells; ↑[TAL], detected only in cells stimulated with TAL; (_1, _2, _3), multiplicity

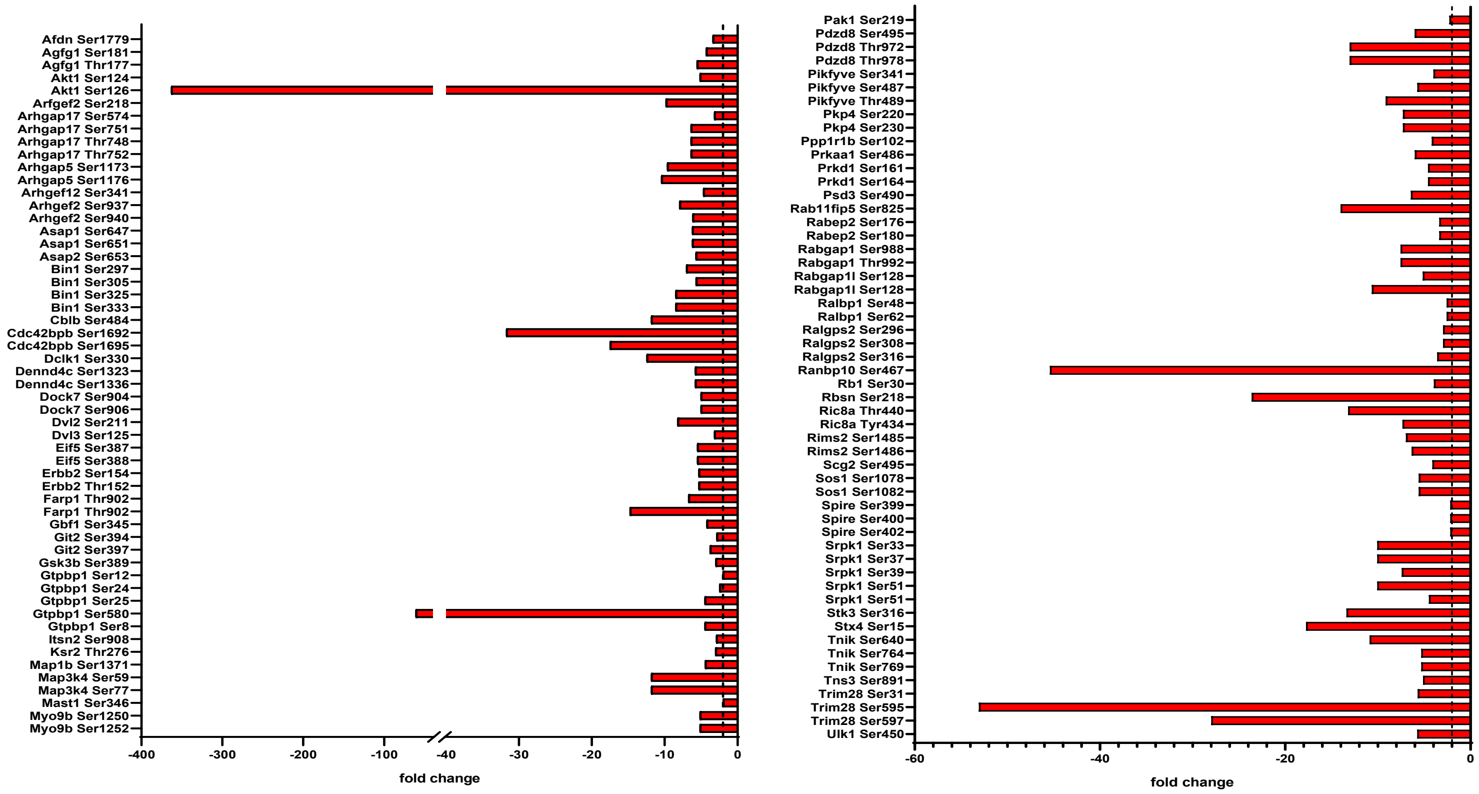

Figure S6. Quantitative changes in phosphoproteins involved in the regulation of small GTPase activity in GH1 cells after stimulation with 1 μM TAL.

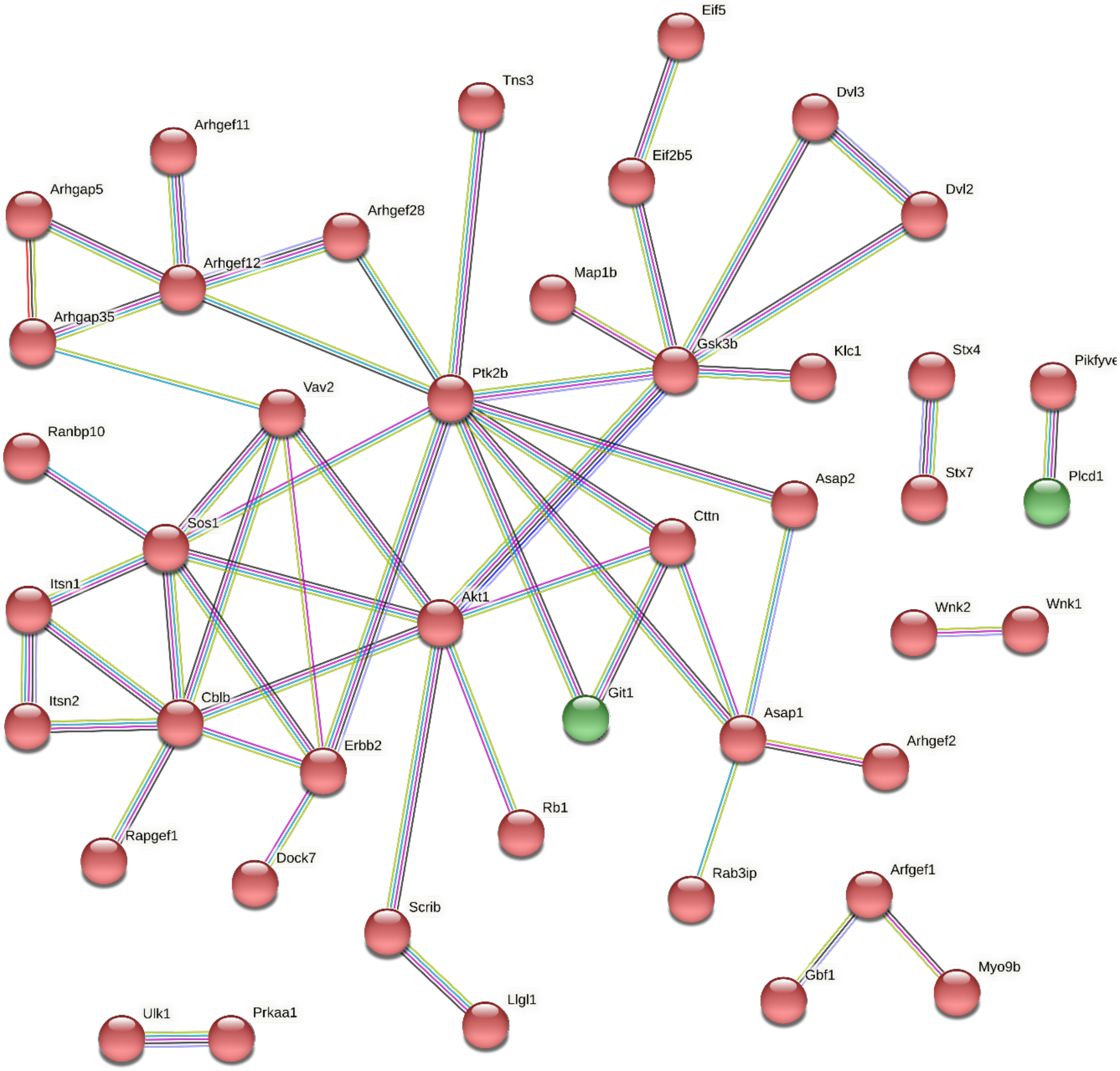

Figure S7. A network of differentially phosphorylated proteins involved in the regulation of small GTPase activity in GH1 cells after TAL treatment. Elevated phosphorylation in green, decreased phosphorylation in red.

Table S4. A list of differentially phosphorylated proteins involved in the regulation of small GTPase activity in GH1 cells after siRNA-mediated β-arrestin2 knockdown and stimulation with 1 μM TRH

Uniprot ID	Gene ID	Gene name	Phosphorylated regions
F1M3G7	Akap13	A-kinase anchoring protein 13	Ser2676 ↑[TRH] (_2)
P47196	Akt1	AKT serine/threonine kinase 1	Ser124 ↓[AR] (_2), Ser126 ↓[AR] (_1)
Q63484	Akt3	AKT serine/threonine kinase 3	Ser2 ↓[AR] (_2), Thr5 ↓[AR] (_2)
F1LM60	Arap1	ArfGAP with RhoGAP domain, ankyrin repeat and PH domain 1	Tyr235 ↑[TRH] (_1)
D4A631	Arfgef1	ADP ribosylation factor guanine nucleotide exchange factor 1	Ser286 ↓[AR] (_1), Ser1566 ↑[TRH] (_1)
D3ZF86	Arfgef3	ARFGEF family member 3	Ser387 ↓[AR] (_2)
A0A0G2JYA2	Arfip1	ADP-ribosylation factor interacting protein 1	Ser5 ↓[AR] (_1), Ser9 ↓[AR] (_1)
D4AAV4	Arhgap17	Rho GTPase activating protein 17	Ser574 ↓[AR] (_1), Thr752 ↑[TRH] (_2)
F1MAK3	Arhgap32	Rho GTPase activating protein 32	Ser720 ↑[TRH] (_1), Ser865 ↑[TRH] (_2), Ser870 ↑[TRH] (_2), Ser885 ↓[AR] (_1)
D4AD82	Arhgap35	Rho GTPase activating protein 35	Ser773 ↓[AR] (_2), Ser985 ↓[AR] (_2), Ser1127 ↓[AR] (_2)
A0A0G2QC21	Arhgef7	Rho guanine nucleotide exchange factor 7	Thr159 ↑[TRH] (_1)
A0A0G2JZC6	Arhgef11	Rho guanine nucleotide exchange factor 11	Ser270 ↑[TRH] (_2), Ser273 ↑[TRH] (_2), Thr311 ↑[TRH] (_2)
P0C6P5	Arhgef28	Rho guanine nucleotide exchange factor 28	Thr1197 ↓[AR] (_2), Ser1198 ↓[AR] (_2), Ser1200 ↓[AR] (_2)
A0A0G2K451	Asap1	ArfGAP with SH3 domain, ankyrin repeat and PH domain 1	Ser855 ↑[TRH] (_1)
O70239	Axin1	axin 1	Ser75 ↑[TRH] (_2), Thr79 ↑[TRH] (_2)
F1LMX1	Bin1	bridging integrator 1	Ser333 ↓[AR] (_1)
Q8K4S7	Cblb	Cbl proto-oncogene B	Ser476 ↑[TRH] (_3), Ser480 ↑[TRH] (_3), Ser484 ↑[TRH] (_3)
B1WC33	Cdc42ep4	CDC42 effector protein 4	Ser116 ↑[TRH] (_1)
Q63768	Crk	CRK proto-oncogene, adaptor protein	Ser41 ↑[TRH] (_1)
A0A1B0GWS4	Cttn	Cortactin	Tyr139 ↑[TRH] (_2)
A0A0G2KB92	Dclk1	doublecortin-like kinase 1	Ser334 ↓[AR] (_3), Ser340 ↑[TRH] (_1) ↓[AR] (_3)
A0A0G2KAH4	Dock6	dedicator of cytokinesis 6	Ser884 ↑[TRH] (_1), Ser1260 ↑[TRH] (_1)
F1LRS2	Dock7	dedicator of cytokinesis 7	Ser904 ↓[AR] (_3), Ser908 ↓[AR] (_3), Ser918 ↓[AR] (_3), Ser1408 ↓[AR] (_1), Ser1410 ↑[TRH] (_1), Ser1416 ↓[AR] (_1)
B5DFE2	Ezh2	enhancer of zeste 2 polycomb repressive complex 2 subunit	Ser362 ↓[AR] (_2)
F1LYQ8	Farp1	FERM, ARH/RhoGEF and pleckstrin domain protein 1	Thr371 ↓[AR] (_2), Ser373 ↓[AR] (_2), Ser376 ↑[TRH] (_2), Ser898 ↑[TRH] (_3), Ser900 ↑[TRH] (_2), Thr902 ↑[TRH] (_3) ↓[AR] (_2)
D3ZFK8	Farp2	FERM, ARH/RhoGEF and pleckstrin domain protein 2	Ser474 ↓[AR] (_2)
Q2HWF0	Fnbp1l	formin binding protein 1-like	Ser488 ↑[TRH] (_2), Ser501 ↑[TRH] (_1)
D4A022	Gapvd1	GTPase activating protein and VPS9 domains 1	Ser971 ↓[AR] (_2)
A0A0G2K3N1	Gbf1	golgi brefeldin A resistant guanine nucleotide exchange factor 1	Ser340 ↓[AR] (_2), Ser1293 ↓[AR] (_1)

A0A0G2K527	Git1	GIT ArfGAP 1	Ser379 ↓[AR] (_2), Thr383 ↓[AR] (_2)
A0A0G2JSH4	Gsk3b	glycogen synthase kinase 3 beta	Ser389 ↓[AR] (_1)
D2XV59	Gtpbp1	GTP binding protein 1	Ser24 ↓[AR] (_3)
Q9WVE9	Itsn1	intersectin 1	Ser896 ↓[AR] (_1)
D3ZM20	Kif13a	kinesin family member 13A	Ser510 ↓[AR] (_3), Ser511 ↓[AR] (_3)
Q6P791	Lamtor1	late endosomal/lysosomal adaptor, MAPK and MTOR activator 1	Ser26 ↓[AR] (_1)
O08873	Madd	MAP-kinase activating death domain	Ser1196 ↑[TRH] (_1)
P15205	Map1b	microtubule-associated protein 1B	Ser1305 ↑[TRH] (_1)
A0A0G2K3R1	Map3k4	mitogen activated protein kinase kinase kinase 4	Ser59 ↓[AR] (_2)
A0JN25	Mapt	microtubule associated protein tau	Ser306 ↑[TRH] (_2)
A0A0G2K6X6	Mark2	microtubule affinity regulating kinase 2	Ser567 ↑[TRH] (_1)
Q810W7	Mast1	microtubule associated serine/threonine kinase 1	Ser346 ↓[AR] (_1)
Q63406	Mcf2l	MCF.2 cell line derived transforming sequence-like	Ser966 ↓[AR] (_2), Thr978 ↓[AR] (_2)
A0A0G2K2Y8	Mpdz	multiple PDZ domain crumbs cell polarity complex component	Ser1819 ↓[AR] (_3)
P35465	Pak1	p21 (RAC1) activated kinase 1	Thr184 ↓[AR] (_1), Ser222 ↓[AR] (_1)
F1M785	Pdzd2	PDZ domain containing 2	Ser887 ↑[TRH] (_2), Ser891 ↑[TRH] (_2), Ser2157 ↓[AR] (_2), Ser2159 ↓[AR] (_2)
D3ZYT8	Pikfyve	phosphoinositide kinase, FYVE-type zinc finger containing	Ser341 ↓[AR] (_1), Ser487 ↓[AR] (_2), Thr489 ↓[AR] (_2)
Q63433	Pkn1	protein kinase N1	Ser920 ↑[TRH] (_1)
Q45QJ4	Plcb3	phospholipase C beta 3	Ser537 ↑[2.00] (_1)
D3ZCI6	Plcb4	phospholipase C, beta 4	Thr886 ↑[TRH] (_3)
D3ZW14	Prex2	phosphatidylinositol-3,4,5-trisphosphate-dependent Rac exchange factor 2	Ser826 ↑[TRH] (_1)
A0A0G2K5Q0	Prkcb	protein kinase C, beta	Ser643 ↑[TRH] (_2)
F1LMV8	Prkce	protein kinase C, epsilon	Ser140 ↑[TRH] (_2)
A0A0G2K928	Prkd1	protein kinase D1	Ser361 ↑[TRH] (_2), Thr364 ↑[TRH] (_2)
P97887	Psen1	presenilin 1	Ser366 ↑[TRH] (_2)
A0A0G2JYK2	Rab11fip1	RAB11 family interacting protein 1	Ser990 ↑[TRH] (_2)
D3ZKH6	Rabgap1l	RAB GTPase activating protein 1-like	Ser128 ↓[AR] (_1)
Q5FVT1	Ralbp1	ralA binding protein 1	Ser30 ↓[AR] (_2), Ser34 ↑[TRH] (_2), Ser92 ↑[TRH] (_3), Ser93 ↑[TRH] (_3), Ser99 ↑[TRH] (_3)
A0A0G2KA57	Ralgapb	Ral GTPase activating protein non-catalytic beta subunit	Thr313 ↓[AR] (_1)
Q0VGK1	Ralgps2	Ral GEF with PH domain and SH3 binding motif 2	Ser293 ↓[AR] (_1), Ser316 ↓[AR] (_2)
D4A2G9	Ranbp1	RAN binding protein 1	Ser21 ↓[AR] (_1)
D4A054	Ranbp2	RAN binding protein 2	Ser1154 ↓[AR] (_1), Ser2092 ↑[TRH] (3), Ser2096 ↑[TRH] (_3)
M0R920	Ranbp3	RAN binding protein 3	Ser27 ↑[TRH] (_3), Ser30 ↑[TRH] (_3), Ser31 ↑[2.92] (_2) ↑[TRH] (_3), Ser409 ↓[AR] (_2)
F1LVV3	Ranbp9	RAN binding protein 9	Ser459 ↓[AR] (_1)

D3Z7Z5	Ranbp10	RAN binding protein 10	Ser463 ↑[TRH] (_3)
F1MAA5	Rangap1	RAN GTPase activating protein 1	Ser427 ↑[TRH] (_2)
D3ZPI4	Rap1gap2	RAP1 GTPase activating protein 2	Ser361 ↑[TRH] (_2)
D3ZTL8	Rapgef6	Rap guanine nucleotide exchange factor 6	Ser1595 ↑[TRH] (_1)
P33568	Rb1	RB transcriptional corepressor 1	Thr363 ↓[AR] (_2), Thr396 ↓[AR] (_2), Ser600 ↓[AR] (_2), Ser604 ↓[AR] (_2)
D3ZL11	Rbsn	rabenosyn, RAB effector	Ser208 ↑[TRH] (_1), Ser216 ↓[AR] (_2)
D3Z8E0	Rps6ka3	ribosomal protein S6 kinase A3	Thr365 ↑[TRH] (_2), Ser369 ↑[TRH] (_2)
Q0D2L6	Rragc	Ras-related GTP binding C	Ser94 ↓[AR] (_1)
D3ZI11	Rreb1	ras responsive element binding protein 1	Thr1595 ↑[TRH] (_3), Ser1597 ↑[TRH] (_3)
G3V7X2	Scg2	secretogranin II	Ser491 ↑[TRH] (_1)
D3ZWS0	Scrib	scribbled planar cell polarity protein	Ser672 ↑[TRH] (_1), Ser1207 ↓[AR] (_2), Ser1209 ↑[TRH] (_3)
A0A0G2KAW2	Sipa1l1	signal-induced proliferation-associated 1 like 1	Ser255 ↓[AR] (_1), Ser1249 ↑[TRH] (_1), Ser1618 ↓[AR] (_2), Ser1624 ↓[-3.00] (_2)
D4A3T0	Sos1	SOS Ras/Rac guanine nucleotide exchange factor 1	Thr1255 ↓[AR] (_2)
D3ZEX7	Spire1	spire-type actin nucleation factor 1	Ser399 ↑[TRH] (_3), Ser400 ↑[TRH] (_3), Ser402 ↑[TRH] (_3)
Q9WUD9	Src	SRC proto-oncogene, non-receptor tyrosine kinase	Ser75 ↓[AR] (_1)
D4A208	Srgap2	SLIT-ROBO Rho GTPase activating protein 2	Ser994 ↓[AR] (_2)
E9PTN4	Srpk1	SRSF protein kinase 1	Ser51 ↓[AR] (_2)
A0A0G2JX62	Srpk2	SRSF protein kinase 2	Ser310 ↓[AR] (_1)
D3ZWV8	Tiam1	T-cell lymphoma invasion and metastasis 1	Ser1462 ↓[AR] (_2)
F1LN42	Tns1	tensin 1	Ser1073 ↓[AR] (_1)
Q9Z136	Tsc1	tuberous sclerosis 1	Ser561 ↓[AR] (_2), Ser565 ↓[AR] (_2)
D3ZLW4	Tsc2	tuberous sclerosis 2	Ser1389 ↓[AR] (_2)
D3ZMJ7	Wnk2	WNK lysine deficient protein kinase 2	Ser49 ↓[AR] (_1)

↑, elevated phosphorylation; ↓, decreased phosphorylation; ↓[AR]. detected only in β-arrestin2-deficient cells; ↑[TRH], detected only in β-arrestin2-deficient cells stimulated with TRH; (_1, _2, _3), multiplicity

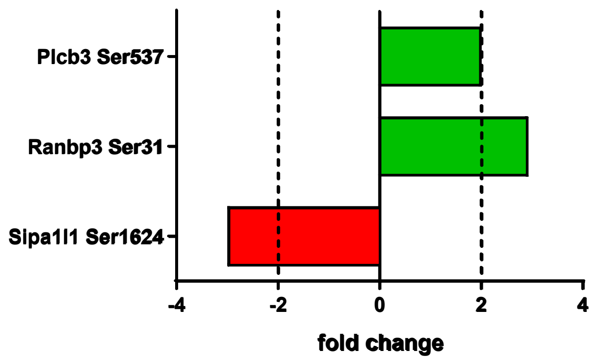

Figure 8. Quantitative changes in phosphoproteins involved in the regulation of small GTPase activity in GH1 cells after siRNA-mediated β-arrestin2 knockdown and stimulation with 1 µM TRH.

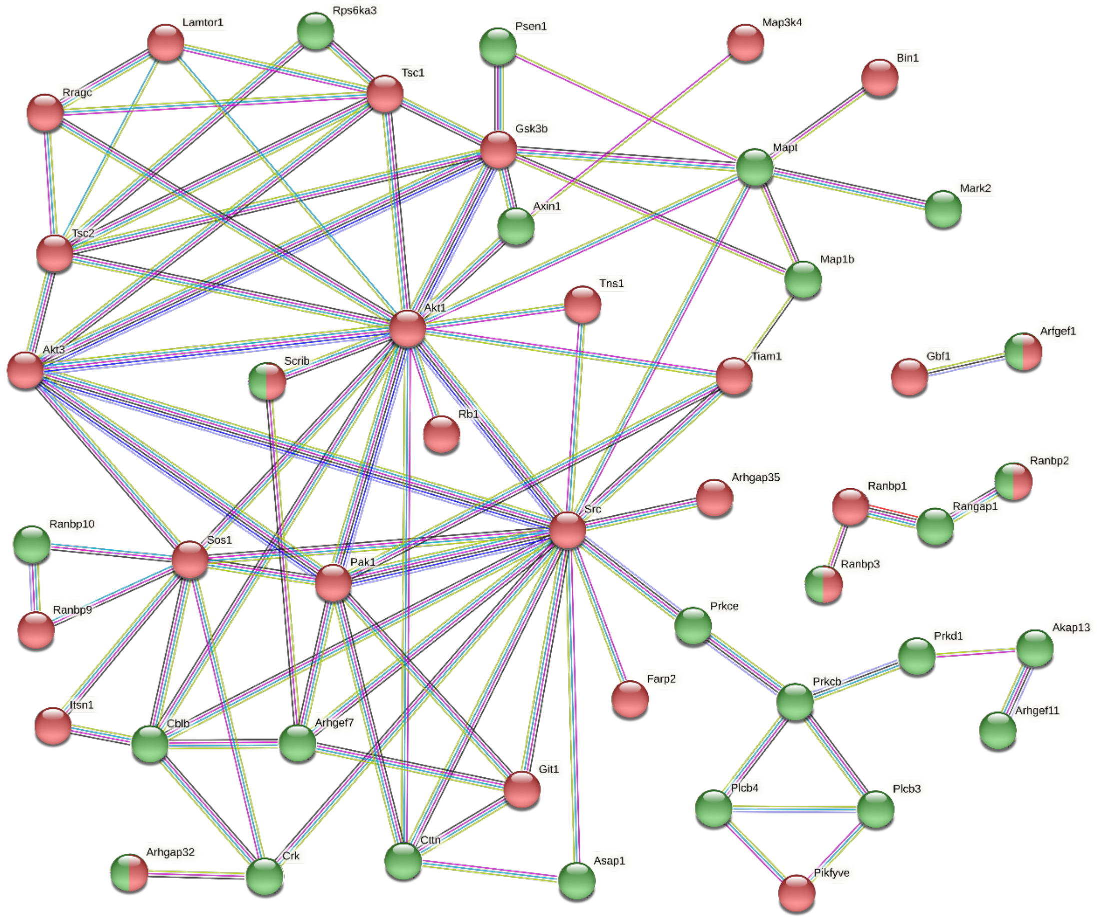

Figure S9. A network of differentially phosphorylated proteins involved in the regulation of small GTPase activity in β-arrestin2-deficient GH1 cells after TRH treatment. Elevated phosphorylation in green, decreased phosphorylation in red.

Table S5. A list of differentially phosphorylated proteins related to regulation of small GTPase activity in GH1 cells after siRNA-mediated β-arrestin2 knockdown and stimulation with 1 μM TAL

Uniprot ID	Gene ID	Gene name	Phosphorylated regions
F1M0N1	Abl2	ABL proto-oncogene 2, non receptor tyrosine kinase	Ser629 ↑[AR] (_1)
A0A0G2K429	Adcy6	adenylate cyclase 6	Thr69 ↓[AR] (_1)
F1M3G7	Akap13	A-kinase anchoring protein 13	Ser2676 ↑[TAL] (_2)
P47196	Akt1	AKT serine/threonine kinase 1	Ser122 ↓[AR] (_3), Ser126 ↓[AR] (_2) ↓[AR] (_1)
D4A631	Arfgef1	ADP ribosylation factor guanine nucleotide exchange factor 1	Ser286 ↓[AR] (_1), Ser393 ↑[TAL] (_2), Ser394 ↑[TAL] (_2)
D3ZF86	Arfgef3	ARFGEF family member 3	Ser387 ↓[AR] (_2)
D4A6C5	Arhgap1	Rho GTPase activating protein 1	Ser51 ↓[AR] (_2)
F1LXQ7	Arhgap21	Rho GTPase activating protein 21	Thr1631 ↓[AR] (_2)
F1MAK3	Arhgap32	Rho GTPase activating protein 32	Ser720 ↑[TAL] (_1), Ser865 ↑[TAL] (_2), Ser870 ↑[TAL] (_2)
D4AD82	Arhgap35	Rho GTPase activating protein 35	Ser773 ↓[AR] (_2), Ser985 ↓[AR] (_2)
A0A0G2QC21	Arhgef7	Rho guanine nucleotide exchange factor 7	Thr159 ↑[TAL] (_1)
A0A0G2JZC6	Arhgef11	Rho guanine nucleotide exchange factor 11	Ser270 ↑[TAL] (_2), Ser273 ↑[TAL] (_2)
D3ZYR0	Arhgef12	Rho guanine nucleotide exchange factor 12	Ser341 ↑[TAL] (_1)
P0C6P5	Arhgef28	Rho guanine nucleotide exchange factor 28	Thr1197 ↓[AR] (_2), Ser1198 ↓[AR] (_2), Ser1200 ↓[AR] (_2)
A0A0G2JZE7	Arhgef40	Rho guanine nucleotide exchange factor 40	Ser958 ↓[AR] (_1)
Q4V8I5	Arl6ip4	ADP-ribosylation factor like GTPase 6 interacting protein 4	Ser142 ↑[TAL] (_1)
A0A0G2K451	Asap1	ArfGAP with SH3 domain, ankyrin repeat and PH domain 1	Ser855 ↑[TAL] (_1)
B2GUV8	Bcl6	B-cell CLL/lymphoma 6	Ser308 ↓[AR] (_2), Ser309 ↓[AR] (_2)
D3ZML2	Brsk2	BR serine/threonine kinase 2	Ser424 ↓[-3.60] (_2), Ser428 ↑[TAL] (_3) ↓[-3.60] (_2), Ser436 ↑[TAL] (_3), Ser490 ↓[AR] (_1)
Q8K4S7	Cblb	Cbl proto-oncogene B	Ser476 ↑[TAL] (_3), Ser480 ↑[TAL] (_3), Ser483 ↑[TAL] (_3)
A0A0G2K5Z1	Cdc42bpa	CDC42 binding protein kinase alpha	Ser1622 ↓[AR] (_2), Ser1625 ↓[AR] (_2)
B1WC33	Cdc42ep4	CDC42 effector protein 4	Ser116 ↑[TAL] (_1)
E2E1S0	Cdkl5	cyclin-dependent kinase-like 5	Ser407 ↓[-2.08] (_1)
G3V8W8	Cnksr1	connector enhacer of kinase suppressor of Ras 1	Thr284 ↓[AR] (_2)
A0A1B0GWS4	Cttn	cortactin	Tyr139 ↑[TAL] (_2)
A0A0G2JTF2	Dab2ip	DAB2 interacting protein	Ser719 ↓[AR] (_1)
A0A0G2KB92	Dclk1	doublecortin-like kinase 1	Thr336 ↓[AR] (_2), Ser340 ↑[TAL] (_1)
F1LTD7	Dennd4c	DENN domain containing 4C	Ser1310 ↑[TAL] (_2)
G3V7Q0	Dennd5a	DENN domain containing 5A	Thr1079 ↓[AR] (_2), Ser1085 ↓[AR] (_2)
D4A544	Dennd6a	DENN domain containing 6A	Ser16 ↓[AR] (_1)
F1LRS2	Dock7	dedicator of cytokinesis 7	Thr917 ↑[TAL] (_3)
G3V6K6	Egfr	epidermal growth factor receptor	Ser1165 ↓[AR] (_1)
F1LYQ8	Farp1	FERM, ARH/RhoGEF and pleckstrin domain protein 1	Ser376 ↑[TAL] (_2), Ser898 ↑[TAL] (_3), Thr902 ↑[TAL] (_3)
D3ZFK8	Farp2	FERM, ARH/RhoGEF and pleckstrin domain protein 2	Thr374 ↓[AR] (_2), Ser474 ↓[AR] (_2), Ser477 ↑[TAL] (_2)

Q2HWF0	Fnbp1l	formin binding protein 1-like	Ser488 ↑[TAL] (_2), Thr496 ↑[TAL] (_1)
A0A0G2K3N1	Gbf1	golgi brefeldin A resistant guanine nucleotide exchange factor 1	Ser340 ↓[AR] (_2), Ser1293 ↓[-59.49] (_1)
A0A0G2K527	Git1	GIT ArfGAP 1	Ser376 ↓[AR] (_2) ↓[AR] (_3)
Q66H91	Git2	GIT ArfGAP 2	Tyr392 ↑[TAL] (_3) ↓[AR] (_2), Ser394 ↑[TAL] (_3), Ser415 ↑[TAL] (_3), Ser418 ↑[TAL] (_3), Ser421 ↑[TAL] (_3)
Q9WVE9	Itsn1	intersectin 1	Ser334 ↓[AR] (_2), Ser335 ↓[AR] (_2), Ser894 ↓[AR] (_1), Ser896 ↓[AR] (_2)
M0R7A6	Itsn2	intersectin 2	Ser903 ↑[TAL] (_1), Ser908 ↑[TAL] (_1)
M0RBD3	Ksr2	kinase suppressor of ras 2	Thr272 ↓[AR] (_2)
Q6P791	Lamtor1	late endosomal/lysosomal adaptor, MAPK and MTOR activator 1	Ser26 ↓[AR] (_1)
G3V6I1	Llgl1	LLGL1, scribble cell polarity complex component	Ser997 ↓[AR] (_3)
O08873	Madd	MAP-kinase activating death domain	Ser1196 ↑[TAL] (_1)
P15205	Map1b	microtubule-associated protein 1B	Ser1305 ↑[TAL] (_1), Ser1371 ↑[TAL] (_1)
A0A0G2K3R1	Map3k4	mitogen activated protein kinase kinase kinase 4	Ser59 ↓[AR] (_3), Ser77 ↓[AR] (_3)
F1M754	Map4k4	mitogen-activated protein kinase kinase kinase kinase 4	Ser852 ↑[TAL] (_1)
A0JN25	Mapt	microtubule associated protein tau	Ser306 ↑[TAL] (_2)
A0A0G2K6X6	Mark2	microtubule affinity regulating kinase 2	Ser392 ↑[TAL] (_1), Ser567 ↑[TAL] (_1)
D4A355	Mastl	microtubule associated serine/threonine kinase-like	Ser588 ↑[TAL] (_1)
A0A0G2K2Y8	Mpdz	multiple PDZ domain crumbs cell polarity complex component	Ser1819 ↓[AR] (_3)
Q4W1H3	Myo9b	myosin IXb	Ser1982 ↑[TAL] (_1)
P35465	Pak1	p21 (RAC1) activated kinase 1	Thr229 ↓[AR] (_2)
F1M785	Pdzd2	PDZ domain containing 2	Ser887 ↑[TAL] (_2), Ser891 ↑[TAL] (_2), Ser2157 ↓[AR] (_2), Ser2159 ↓[AR] (_2)
D3ZXY2	Pdzd8	PDZ domain containing 8	Thr972 ↓[AR] (_3), Ser973 ↓[AR] (_3), Ser978 ↓[AR] (_3)
D3ZYT8	Pikfyve	phosphoinositide kinase, FYVE-type zinc finger containing	Ser487 ↓[AR] (_2) ↓[AR] (_1), Thr489 ↓[AR] (_2) ↓[AR] (_1)
Q63433	Pkn1	protein kinase N1	Ser536 ↓[AR] (_2), Ser920 ↑[TAL] (_1)
F1M2K6	Pkp4	plakophilin 4	Ser220 ↓[AR] (_2), Ser230 ↓[AR] (_2)
D3ZCI6	Plcb4	phospholipase C, beta 4	Thr886 ↑[TAL] (_3)
G3V9D1	Plcd1	phospholipase C, delta 1	Thr457 ↑[TAL] (_2)
D3ZW14	Prex2	phosphatidylinositol-3,4,5-trisphosphate-dependent Rac exchange factor 2	Ser826 ↑[TAL] (_1)
P54645	Prkaa1	protein kinase AMP-activated catalytic subunit alpha 1	Thr488 ↓[AR] (_2), Thr526 ↑[TAL] (_2), Ser527 ↑[TAL] (_3)
A0A0G2K5Q0	Prkcb	protein kinase C, beta	Ser643 ↑[TAL] (_2)
F1LMV8	Prkce	protein kinase C, epsilon	Ser140 ↑[TAL] (_2), Ser148 ↑[TAL] (_2)
A0A0G2K928	Prkd1	protein kinase D1	Ser361 ↑[TAL] (_2), Thr364 ↑[TAL] (_2)
P97887	Psen1	presenilin 1	Ser368 ↑[TAL] (_3), Thr371 ↑[TAL] (_3), Ser372 ↑[TAL] (_3)

P70600	Ptk2b	protein tyrosine kinase 2 beta	Ser389 ↓[AR] (_3), Ser392 ↓[AR] (_3), Ser394 ↓[AR] (_3), Ser396 ↓[AR] (_3), Ser399 ↓[AR] (_3)
A0A0G2K1B4	Rab3ip	RAB3A interacting protein	Ser247 ↓[AR] (_2)
A0A0G2JYK2	Rab11fip1	RAB11 family interacting protein 1	Ser990 ↑[TAL] (_2)
D3ZX42	Rabgap1	RAB GTPase activating protein 1	Ser988 ↑[TAL] (_2), Thr992 ↑[TAL] (_2)
D3ZKH6	Rabgap1l	RAB GTPase activating protein 1-like	Ser128 ↑[8.98] (_1)
Q5FVT1	Ralbp1	ralA binding protein 1	Ser30 ↓[AR] (_2), Ser34 ↑[TAL] (_2), Ser92 ↑[TAL] (_3), Ser93 ↑[TAL] (_3), Ser99 ↑[TAL] (_3)
Q0VGK1	Ralgps2	Ral GEF with PH domain and SH3 binding motif 2	Ser293 ↓[AR] (_1), Ser308 ↑[TAL] (_1), Ser315 ↓[AR] (_3)
D4A2G9	Ranbp1	RAN binding protein 1	Ser21 ↓[AR] (_1)
D4A054	Ranbp2	RAN binding protein 2	Ser1154 ↓[AR] (_1), Ser2088 ↑[TAL] (_3), Ser2092 ↑[TAL] (_3), Ser2096 ↑[TAL] (_3), Ser2097 ↑[TAL] (_3) ↓[AR] (_2)
M0R920	Ranbp3	RAN binding protein 3	Ser27 ↑[TAL] (_3), Ser30 ↑[TAL] (_3), Ser31 ↑[TAL] (_3) ↑[2.47] (_2)
F1LVV3	Ranbp9	RAN binding protein 9	Ser459 ↓[AR] (_1)
D3Z7Z5	Ranbp10	RAN binding protein 10	Ser463 ↑[TAL] (_3), ↓[AR] (_2)
F1MAA5	Rangap1	RAN GTPase activating protein 1	Ser427 ↑[TAL] (_2)
D3ZPI4	Rap1gap2	RAP1 GTPase activating protein 2	Ser361 ↑[TAL] (_2)
D3ZTL8	Rapgef6	Rap guanine nucleotide exchange factor 6	Ser1237 ↑[TAL] (_2), Ser1241 ↓[AR] (_3), Ser1245 ↓[AR] (_3), Ser1595 ↑[TAL] (_1)
O35141	Rassf5	Ras association domain family member 5	Ser177 ↑[TAL] (_1)
P33568	Rb1	RB transcriptional corepressor 1	Thr363 ↓[AR] (_2), Thr366 ↓[AR] (_2), Ser600 ↓[AR] (_2), Ser604 ↓[AR] (_2)
D3ZL11	Rbsn	rabenosyn, RAB effector	Ser208 ↑[TAL] (_1), Ser216 ↓[AR] (_2)
Q0D2L6	Rragc	Ras-related GTP binding C	Ser94 ↓[AR] (_1)
D3ZI11	Rreb1	ras responsive element binding protein 1	Ser1361 ↑[TAL] (_2), Ser1364 ↑[TAL] (_2)
G3V7X2	Scg2	secretogranin II	Ser495 ↑[TAL] (_1)
D3ZWS0	Scrib	scribbled planar cell polarity protein	Ser672 ↑[TAL] (_1), Ser1207 ↓[AR] (_2), Ser1209 ↓[AR] (_2)
M0R617	Sh2b1	SH2B adaptor protein 1	Ser126 ↓[AR] (_3), Ser127 ↓[AR] (_3)
Q9Z200	Sh2b2	SH2B adaptor protein 2	Ser584 ↓[AR] (_2)
E9PSX8	Sipa1	signal-induced proliferation-associated 1	Ser815 ↑[TAL] (_1)
A0A0G2KAW2	Sipa1l1	signal-induced proliferation-associated 1 like 1	Ser1249 ↑[TAL] (_1), Ser1543 ↑[TAL] (_2)
D4A3T0	Sos1	SOS Ras/Rac guanine nucleotide exchange factor 1	Ser1078 ↑[TAL] (_2), Ser1082 ↑[TAL] (_2), Thr1255 ↓[AR] (_2), Ser1319 ↓[AR] (_1)
D3ZEX7	Spire1	spire-type actin nucleation factor 1	Ser399 ↑[TAL] (_3), Ser400 ↑[TAL] (_3), Ser402 ↑[TAL] (_3)
Q9WUD9	Src	SRC proto-oncogene, non-receptor tyrosine kinase	Ser75 ↓[AR] (_1)
D4A208	Srgap2	SLIT-ROBO Rho GTPase activating protein 2	Thr998 ↑[TAL] (_2)
E9PTN4	Srpk1	SRSF protein kinase 1	Ser51 ↓[AR] (_2), Ser311 ↑[TAL] (_1)
A0A0G2JX62	Srpk2	SRSF protein kinase 2	Ser310 ↓[AR] (_1)
F1LRL4	Tbc1d9b	TBC1 domain family member 9B	Ser1084 ↑[TAL] (_2) ↓[AR] (_1), Ser1089 ↑[TAL] (_2)

D3ZSY8	Tbc1d10b	TBC1 domain family, member 10b	Ser128 ↓[AR] (_3), Thr135 ↓[AR] (_3), Thr137 ↑[TAL] (_3), Thr139 ↓[AR] (_3), Ser231 ↑[TAL] (_2), Thr232 ↑[TAL] (_2)
D3ZWV8	Tiam1	T-cell lymphoma invasion and metastasis 1	Ser725 ↓[AR] (_1), Ser1462 ↓[AR] (_2)
F1LN91	Tns3	tensin 3	Ser891 ↓[AR] (_1)
Q9Z136	Tsc1	tuberous sclerosis 1	Ser561 ↓[AR] (_2), Ser565 ↓[AR] (_2), Ser1097 ↑[TAL] (_1)
A0A0G2K3A0	Wnk1	WNK lysine deficient protein kinase 1	Ser1809 ↓[AR] (_2)
D3ZMJ7	Wnk2	WNK lysine deficient protein kinase 2	Ser1830 ↓[AR] (_2), Ser1831 ↓[AR] (_2)
A0A1W2Q6C5	Wnk3	WNK lysine deficient protein kinase 3	Ser436 ↓[AR] (_3), Thr449 ↓[AR] (_3)

↑, elevated phosphorylation; ↓, decreased phosphorylation; ↓[AR]. detected only in β-arrestin2-deficient cells; ↑[TAL], detected only in β-arrestin2-deficient cells stimulated with TAL; (_1, _2, _3), multiplicity

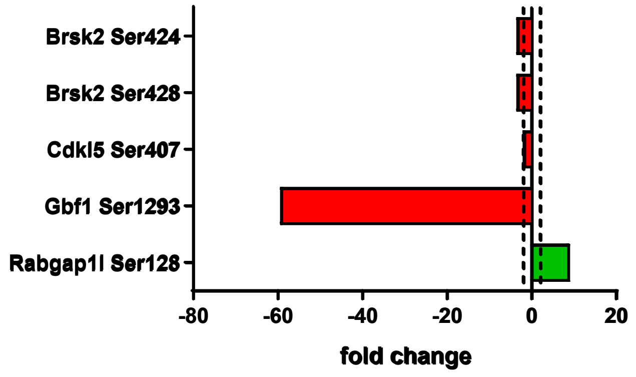

Figure S10. Quantitative changes in phosphoproteins involved in the regulation of small GTPase activity in GH1 cells after siRNA-mediated β-arrestin2 knockdown and stimulation with 1 µM TAL.

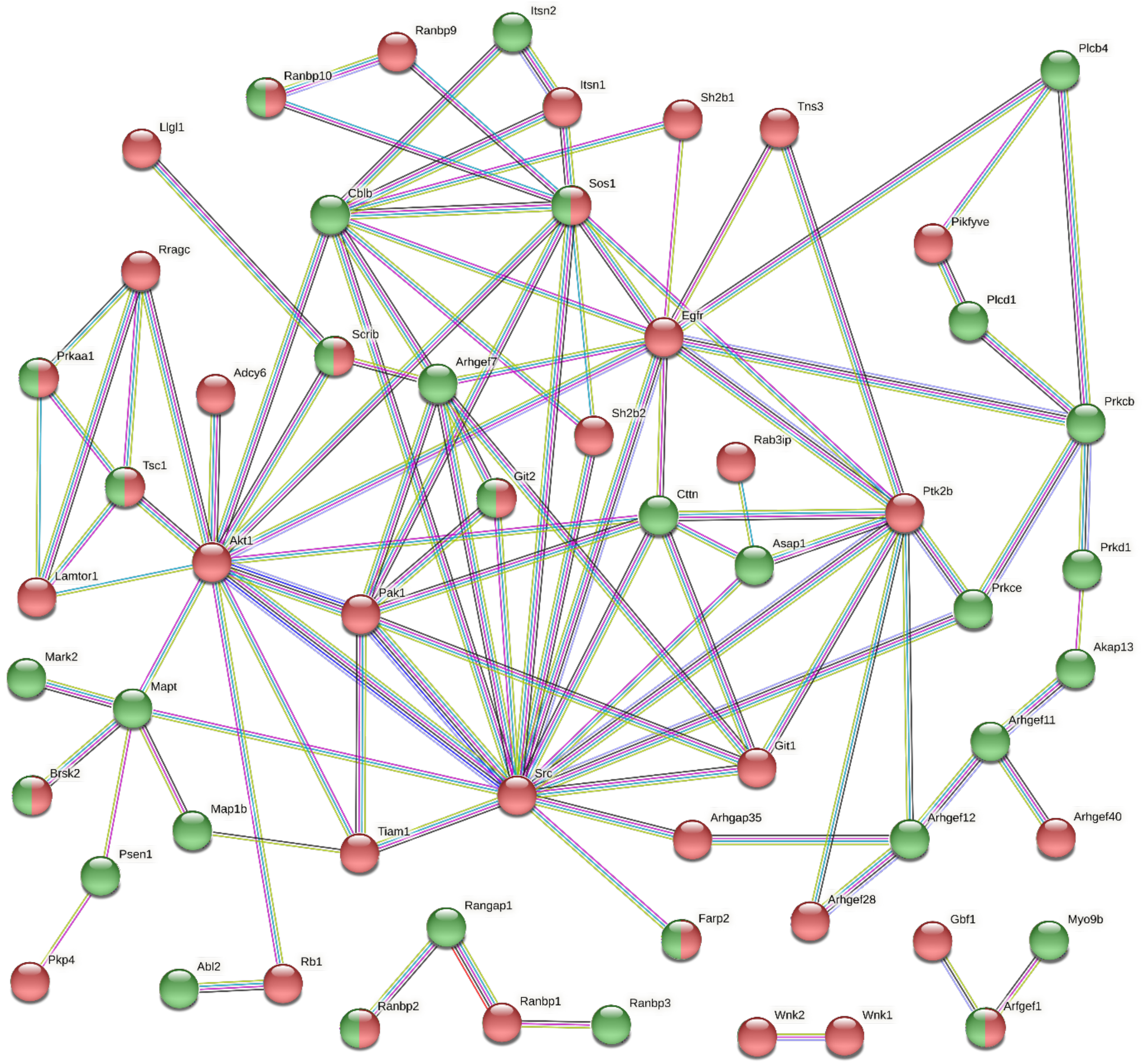

Figure S11. A network of differentially phosphorylated proteins involved in the regulation of small GTPase activity in β-arrestin2-deficient GH1 cells after TAL treatment. Elevated phosphorylation in green, decreased phosphorylation in red.

Table S6. A list of differentially phosphorylated proteins involved in MAP kinase-mediated signaling in GH1 cells after siRNA-mediated β-arrestin2 knockdown

Uniprot ID	Gene ID	Gene name	Phosphorylated regions
F1LRI7	Aak1	AP2 associated kinase 1	Thr608 ↑[12.88] (_1)
F1M0N1	Abl2	ABL proto-oncogene 2, non-receptor tyrosine kinase	Ser629 ↑[70.75] (_1)
G3V6M0	Akap6	A-kinase anchoring protein 6	Ser423 ↑[AR] (_1), Ser2208 ↑[AR] (_2)
F1LPB4	Akap9	A-kinase anchoring protein 9	Ser1500 ↓[C](_1)
F1LR81	Akap11	A-kinase anchoring protein 11	Ser434 ↓[C] (_3), Ser439 ↓[C] (_3), Ser440 ↓[C] (_3)
P47196	Akt1	AKT serine/threonine kinase 1	Ser124 ↑[169.68] (_2), Ser 124 ↑[93.14] (_1), Ser126 ↑[AR] (_2), Ser126 ↑[155.76] (_3), Ser129 ↑[155.76] (_3)
F1M7M4	Bmp2k	BMP-2 inducible kinase	Thr757 ↑[AR] (_2), Ser934 ↑[AR] (_2), Ser936 ↑[AR] (_2)
A0A0G2JYV0	Brd4	bromodomain containing 4	Ser471 ↑[AR] (_1), Ser1085 ↑[104.47] (_3)
B2DD29	Brsk1	BR serine/threonine kinase 1	Ser490 ↑[AR] (_1), Thr583 ↓[C] (_2), Ser587 ↓[C] (_2)
D3ZML2	Brsk2	BR serine/threonine kinase 2	Ser424 ↑[13.84] (_2), Ser428 ↓[C] (_3) ↑[13.84] (_2), Ser436 ↓[C] (_3) ↑[AR] (_2), Ser440 ↑[AR] (_2), Ser490 ↑[AR] (_1)
F1LNI8	Camk2b	calcium/calmodulin-dependent protein kinase II beta	Ser355 ↑[AR] (_3), Ser356 ↑[AR] (_3), Ser358 ↓[C] (_2) ↑[AR] (_3)
A0A0G2K5C0	Camsap3	calmodulin regulated spectrin-associated protein family, member 3	Ser319 ↑[AR] (_2), Ser320 ↓[C] (_1)
D3ZYD7	Ccdc88a	coiled coil domain containing 88A	Ser1319↓[C] (_1), Ser1343 ↑[AR] (_1)
F1M4U0	Ccnyl1	cyclin Y-like 1	Ser349 ↑[AR] (_1)
A0A0G2K5Z1	Cdc42bpa	CDC42 binding protein kinase alpha	Ser1618 ↑[AR] (_3), Ser1622 ↑[AR] (_2), Ser1625 ↑[AR] (_2)
Q7TT49	Cdc42bpb	CDC42 binding protein kinase beta	Ser1688 ↑[5.81] (_3), Ser1692 ↑[5.81] (_3), Ser1695 ↑[5.81] (_3)
Q6P751	Cdk2	cyclin dependent kinase 2	Thr14 ↑[134.24] (_2), Tyr15 ↑[134.24] (_2)
P35426	Cdk4	cyclin-dependent kinase 4	Ser300 ↑[AR] (_1)
D4A3G2	Cdk11b	cyclin-dependent kinase 11B	Tyr751 ↑[AR] (_2)
A0A0G2K5U7	Cdk12	cyclin-dependent kinase 12	Ser331 ↑[7.72] (_2), Ser333 ↑[AR] (_2), Ser378 ↑[AR] (_3), Ser381 ↑[AR] (_3), Ser382 ↑[468.89] (_2), Ser384 ↑[434.23] (_2), Ser681 ↑[6.69] (_2)
A0A0H2UHG4	Cdk18	cyclin-dependent kinase 18	Ser65 ↑[157.60] (_1), Ser80 ↑[5.04] (_1)
E2E1S0	Cdkl5	cyclin-dependent kinase-like 5	Ser407 ↑[17.43] (_1)
D4ADG3	Clk1	CDC-like kinase 1	Ser137 ↑[41.56] (_2), Ser139 ↑[41.56] (_2)
D3ZHC3	Clspn	claspin	Ser77 ↑[AR] (_3)
D3ZKK3	Cnst	consortin, connexin sorting protein	Ser117 ↓[C] (_1), Ser119 ↑[17.93] (_1)
A0A096MJD3	Csnk2b	casein kinase 2 beta	Ser154 ↑[AR] (_1)

A0A0G2JTF2	Dab2ip	DAB2 interacting protein	Ser719 ↑[AR] (_1)
Q6MGC8	Daxx	death-domain associated protein	Ser521 ↓[C] (_2), Ser526 ↓[C] (_2)
A0A0G2KB92	Dclk1	doublecortin-like kinase 1	Ser305 ↑[AR] (_2), Ser307 ↑[AR] (_2), Ser332 ↑[AR] (_3), Thr336 ↑[AR] (_2), Ser337 ↑[AR] (_3)
Q9WVB9	Dvl1	dishevelled segment polarity protein 1	Ser194 ↑[AR] (_1)
D3ZB71	Dvl2	dishevelled segment polarity protein 2	Ser211 ↓[C] (_1)
D4ADV8	Dvl3	dishevelled segment polarity protein 3	Ser48 ↑[AR] (_1)
D4ACC4	Dyrk1b	dual specificity tyrosine phosphorylation regulated kinase 1B	Tyr273 ↑[AR] (_1)
G3V6K6	Egfr	epidermal growth factor receptor	Ser1165 ↑[AR] (_1)
D4A554	Eif4g3	eukaryotic translation initiation factor 4 gamma, 3	Ser305 ↑[AR] (_1), Ser1188 ↑[AR] (_1)
G3V6N1	Erbb3	erb-b2 receptor tyrosine kinase 3	Ser980 ↑[AR] (_2), Thr984 ↑[AR] (_2)
B5DFE2	Ezh2	enhancer of zeste 2 polycomb repressive complex 2 subunit	Ser362 ↑[AR] (_2), Ser363 ↑[AR] (_2)
F1LYQ8	Farp1	FERM, ARH/RhoGEF and pleckstrin domain protein 1	Thr371 ↑[AR] (_2), Ser373 ↑[AR] (_2), Ser893 ↑[16.79] (_3), Thr902 ↓[C] (_3), Ser903 ↑[64.70] (_3)
F1LMD9	Gak	cyclin G associated kinase	Ser824 ↓[C] (_2), Ser827 ↓[C] (_2)
D3ZUP4	Gpatch2l	G patch domain containing 2-like	Ser86 ↓[C] (_2), Ser88 ↓[C] (_2)
P51639	Hmgcr	3-hydroxy-3-methylglutaryl-CoA reductase	Ser356 ↑[AR] (_2), Thr360 ↑[AR] (_2)
A0A0G2K2T6	Ilf3	interleukin enhancer binding factor 3	Thr67 ↑[AR] (_1)
F1LZV1	Kalrn	kalirin, RhoGEF kinase	Ser1790 ↑[AR] (_1), Ser1808 ↑[AR] (_1)
Q9EQG6	Kidins220	kinase D-interacting substrate 220	Ser1513 ↓[C] (_2), Ser1518 ↓[C] (_2), Thr1520 ↑[AR] (_2)
M0RBD3	Ksr2	kinase suppressor of ras 2	Thr272 ↑[19.47] (_2)
F1M2K4	Lats1	large tumor suppressor kinase 1	Ser464 ↑[AR] (_1), Ser1111 ↓[C] (_1)
D3ZBH5	Lmtk2	lemur tyrosine kinase 2	Ser576 ↑[AR] (_2), Ser704 ↑[AR] (_1), Ser712 ↑[AR] (_2), Ser746 ↑[7.46] (_2), Ser750 ↑[9.64] (_2), Ser756 ↑[AR] (_2), Ser1035 ↑[AR] (_1), Ser1334 ↑[AR] (_3), Ser1524 ↑[AR] (_2), Ser1525 ↑[AR] (_2)
F1M0A6	LOC100909750	tyrosine-protein kinase ABL1-like	Ser798 ↓[C] (_3), Ser801 ↓[C] (_2)
D4A930	Maml1	mastermind-like transcriptional coactivator 1	Ser88 ↑[AR] (_1)
F1M9D0	Map3k2	mitogen activated protein kinase kinase kinase 2	Ser163 ↑[AR] (_1), Ser239 ↑[AR] (_1), Ser331 ↑[AR] (_1), Thr337 ↑[AR] (_2), Ser344 ↑[AR] (_2)
D3ZW27	Map3k5	mitogen-activated protein kinase kinase kinase 5	Ser1027 ↑[AR] (_2), Ser1031 ↑[AR] (_2)
P0C8E4	Map3k7	mitogen activated protein kinase kinase kinase 7	Ser439 ↑[AR] (_2)
A0A0G2JUN9	Map3k9	mitogen-activated protein kinase kinase kinase 9	Ser541 ↑[AR] (_2), Ser545 ↑[AR] (_2)
D3Z8I4	Map4k1	mitogen activated protein kinase kinase kinase kinase 1	Ser373 ↓[C] (_2), Ser375 ↓[C] (_2), Tyr379 ↑[4.06] (_2)

G3V9M2	Mapk8ip2	mitogen-activated protein kinase 8 interacting protein 2	Ser254 ↑[AR] (_2)
A0A0G2K7H9	Mark1	microtubule affinity regulating kinase 1	Ser348 ↑[AR] (_2)
F1M836	Mark3	microtubule affinity regulating kinase 3	Ser469 ↑[8.07] (_1)
D3ZL30	Mast3	microtubule associated serine/threonine kinase 3	Ser354 ↑[12.14] (_1), Ser1201 ↑[AR] (_2), Ser1213 ↑[AR] (_2)
A0A0G2K382	Mink1	misshapen-like kinase 1	Ser760 ↑[AR] (_1), Ser777 ↑[AR] (_2), Ser781 ↑[AR] (_2)
G3V968	Mphosph10	M-phase phosphoprotein 10	Ser164 ↓[C] (_2)
A0A140TA95	Mprip	myosin phosphatase Rho interacting protein	Ser230 ↑[9.91] (_2), Ser294 ↑[AR] (_2)
A0A0G2JX74	Mtor	mechanistic target of rapamycin	Ser2478 ↑[AR] (_2), Ser2481 ↑[AR] (_2)
A0A0G2JSU4	Ndrg2	NDRG family member 2	Ser314 ↑[AR] (_3), Ser318 ↑[AR] (_2), Ser324 ↑[AR] (_2), Ser336 ↓[C] (_3)
A0A0G2JWL3	Nf1	neurofibromin 1	Ser821 ↑[AR] (_2), Ser824 ↑[AR] (_2), Ser2488 ↑[AR] (_1)
Q63035	Nlrp6	NLR family, pyrin domain containing 6	Thr719 ↑[43.01] (_3), Thr728 ↑[43.01] (_3)
G3V7Z8	Pabpn1	poly(A) binding protein, nuclear 1	Ser91 ↑[AR] (_1)
P35465	Pak1	p21 (RAC1) activated kinase 1	Thr184 ↑[AR] (_1), Ser219 ↑[AR] (_2), Ser222 ↑[AR] (_1), Thr228 ↓[C] (_1), Thr229 ↑[AR] (_2)
B5DF62	Pak4	p21 (RAC1) activated kinase 4	Ser181 ↑[AR] (_1)
D3ZQ51	Pak6	p21 (RAC1) activated kinase 6	Ser328 ↑[AR] (_1), Ser346 ↑[AR] (_2), Ser347 ↑[AR] (_2), Ser351 ↑[AR] (_2)
Q63433	Pkn1	protein kinase N1	Ser920 ↓[C] (_1)
A0A0G2K6J2	Pkn2	protein kinase N2	Ser468 ↑[22.69] (_1)
A0A0H2UHA0	Ppp1r2	protein phosphatase 1, regulatory (inhibitor) subunit 2	Ser47 ↑[57.29] (_1), Ser47 ↑[AR] (_2), Ser49 ↑[AR] (_2), Ser81 ↑[15.99] (_2), Ser82 ↑[15.99] (_2)
O35274	Ppp1r9b	protein phosphatase 1, regulatory subunit 9B	Ser100 ↑[AR] (_1)
Q99MC0	Ppp1r14a	protein phosphatase 1, regulatory (inhibitor) subunit 14A	Ser136 ↓[C] (_1)
D3ZG37	Ppp6r1	protein phosphatase 6, regulatory subunit 1	Thr524 ↑[22.12] (_3), Ser529 ↓[C] (_2) ↑[9.58] (_3), Ser530 ↓[C] (_2) ↑[9.58] (_3), Ser531 ↑[291.69] (_3), Ser740 ↑[AR] (_2), Ser746 ↑[AR] (_2)
D3ZBT9	Ppp6r3	protein phosphatase 6, regulatory subunit 3	Thr518 ↓[C] (_3), Ser523 ↑[45.78] (_2), Ser525 ↑[AR] (_3)
P54645	Prkaa1	protein kinase AMP-activated catalytic subunit alpha 1	Thr488 ↑[AR] (_2), Ser527 ↓[C] (_3) ↑[AR] (_2)
P80386	Prkab1	protein kinase AMP-activated non-catalytic subunit beta 1	Ser108 ↑[AR] (_1)
P09456	Prkar1a	protein kinase cAMP-dependent type 1 regulatory subunit alpha	Ser83 ↑[AR] (_1)
A0A0G2K405	Prkar2a	protein kinase cAMP-dependent type 2 regulatory subunit alpha	Ser97 ↑[AR] (_1)
A0A0G2K5Q0	Prkcb	protein kinase C, beta	Ser639 ↑[AR] (_1)
D4A0U0	Prkcd	protein kinase C, delta	Ser642 ↑[AR] (_1)

F1LMV8	Prkce	protein kinase C, epsilon	Ser140 ↓[C] (_2)
A0A0G2K928	Prkd1	protein kinase D1	Ser161 ↑[24.30] (_2), Ser164 ↑[24.30] (_2), Ser361 ↓[C] (_2), Ser361 ↑[25.40] (_1), Thr364 ↓[C] (_2)
Q5XIS9	Prkd2	protein kinase D2	Ser197 ↑[AR] (_2), Ser198 ↑[AR] (_2), Ser206 ↑[AR] (_2), Ser711 ↑[AR] (_1)
Q5RKH1	Prpf4b	pre-mRNA processing factor 4B	Ser21 ↓[C] (_3), Ser24 ↓[C] (_3), Ser33 ↓[C] (_3), Ser143 ↑[25.60] (_2), Ser145 ↑[25.60] (_2), Ser366 ↑[64.57] (_2), Ser368 ↑[64.57] (_2), Ser576 ↑[AR] (_3), Ser578 ↑[AR] (_3), Ser580 ↑[AR] (_3)
A0A0G2K064	Ptpn6	protein tyrosine phosphatase, non-receptor type 6	Ser10 ↑[AR] (_1)
F1LVV3	Ranbp9	RAN binding protein 9	Ser440 ↑[AR] (_3), Ser446 ↑[57.73] (_2), Ser459 ↑[AR] (_1)
F1M8L9	Rapgef1	Rap guanine nucleotide exchange factor 1	Ser375 ↑[AR] (_1)
D3ZHK4	Rb1cc1	RB1-inducible coiled-coil 1	Ser237 ↑[AR] (_1), Thr238 ↑[AR] (_2), Ser243 ↑[AR] (_2)
D3ZN37	Rock1	Rho-associated coiled-coil containing protein kinase 1	Ser1105 ↑[8.38] (_2)
F1LQT3	Rock2	Rho-associated coiled-coil containing protein kinase 2	Ser1124 ↑[AR] (_2) ↑[AR] (_1), Ser1127 ↑[6.58] (_2)
D3Z8E0	Rps6ka3	ribosomal protein S6 kinase A3	Ser715 ↑[AR] (_1)
A0A0G2KB60	Rps6kc1	ribosomal protein S6 kinase C1	Ser635 ↓[C] (_2), Ser639 ↓[C] (_2)
D3ZDU2	Rptor	regulatory associated protein of MTOR, complex 1	Thr857 ↑[AR] (_2), Ser859 ↓[C] (_2)
F1LU97	Sash1	SAM and SH3 domain containing 1	Ser534 ↑[AR] (_2), Ser541 ↓[C] (_2)
G3V7X2	Scg2	secretogranin II	Ser176 ↑[AR] (_1), Ser495 ↓[C] (_1)
D4AEB3	Setx	senataxin	Ser997 ↑[AR] (_2), Ser999 ↑[AR] (_2)
M0RD40	Sik3	SIK family kinase 3	Ser493 ↑[AR] (_1), Ser534 ↑[AR] (_1), Ser856 ↑[AR] (_1)
Q9JJ19	Slc9a3r1	SLC9A3 regulator 1	Ser287 ↑[AR] (_1)
Q63553	Snrk	SNF related kinase	Ser569 ↑[AR] (_1)
P07632	Sod1	superoxide dismutase 1, soluble	Ser99 ↑[AR] (_1)
D3ZJA3	Spry4	sprouty RTK signaling antagonist 4(Spry4)	Ser126 ↑[AR] (_1)
O08623	Sqstm1	sequestosome 1	Thr266 ↓[C] (_2), Ser354 ↑[AR] (_2), Ser364 ↓[C] (_1)
Q9WUD9	Src	SRC proto-oncogene, non-receptor tyrosine kinase	Ser75 ↑[10.91] (_1)
E9PTN4	Srpk1	SRSF protein kinase 1	Ser51 ↑[3.40] (_2), Ser309 ↑[AR] (_2), Ser311 ↑[AR] (_2)
A0A0G2JX62	Srpk2	SRSF protein kinase 2	Ser487 ↑[AR] (_2), Thr491 ↑[AR] (_2)
B1WBQ5	Stk3	serine/threonine kinase 3	Ser316 ↑[29.15] (_1)
F1LRI6	Taok3	TAO kinase 3	Ser324 ↑[AR] (_1)
D3ZWV8	Tiam1	T-cell lymphoma invasion and metastasis 1	Ser725 ↑[AR] (_1), Ser1462 ↑[AR] (_2)
F1LPP2	Tlk2	tousled-like kinase 2	Ser749 ↑[AR] (_1)

D3ZZQ0	Tnik	TRAF2 and NCK interacting kinase	Ser640 ↑[15.17] (_1), Ser678 ↑[AR] (_2), Ser680 ↑[AR] (_2), Ser769 ↑[AR] (_1)
F7ESX8	Trim24	tripartite motif-containing 24	Ser183 ↑[29.79] (_2), Ser190 ↑[29.79] (_2), Ser267 ↑[AR] (_1)
O08629	Trim28	tripartite motif-containing 28	Ser27 ↑[AR] (_2), Ser52 ↑[AR] (_1), Ser502 ↑[AR] (_1), Ser595 ↑[AR] (_2) ↓[-46.22] (_3), Ser597 ↓[-38.19] (_3)
A0A1P0PBZ6	Trio	trio Rho guanine nucleotide exchange factor	Ser2408 ↑[AR] (_2), Ser2412 ↑[AR] (_2)
Q9Z136	Tsc1	tuberous sclerosis 1	Ser561 ↑[AR] (_2), Ser565 ↑[AR] (_2)
D3ZMG0	Ulk1	unc-51 like autophagy activating kinase 1	Ser757 ↑[AR] (_2)
Q66HC2	Vrk3	vaccinia related kinase 3	Ser82 ↑[11.18] (_3), Ser83 ↑[11.18] (_3), Ser85 ↑[11.18] (_3), Thr88 ↓[C] (_3)
Q63802	Wee1	WEE1 G2 checkpoint kinase	Ser78 ↑[AR] (_2), Ser85 ↑[AR] (_2)
A0A0G2K3A0	Wnk1	WNK lysine deficient protein kinase 1	Ser2154 ↑[AR] (_1)
D3ZMJ7	Wnk2	WNK lysine deficient protein kinase 2	Ser45 ↑[AR] (_1), Ser1774 ↑[AR] (_1), Ser1830 ↑[16.53] (_2), Ser1831 ↑[16.53] (_2)
A0A1W2Q6C5	Wnk3	WNK lysine deficient protein kinase 3	Ser436 ↑[AR] (_3), Thr449 ↑[AR] (_3)

↑, elevated phosphorylation; ↓, decreased phosphorylation; ↓[C], detected only in control (negative siRNA-treated) cells; ↓[AR]. detected only in β-arrestin2-deficient cells; (_1, _2, _3), multiplicity

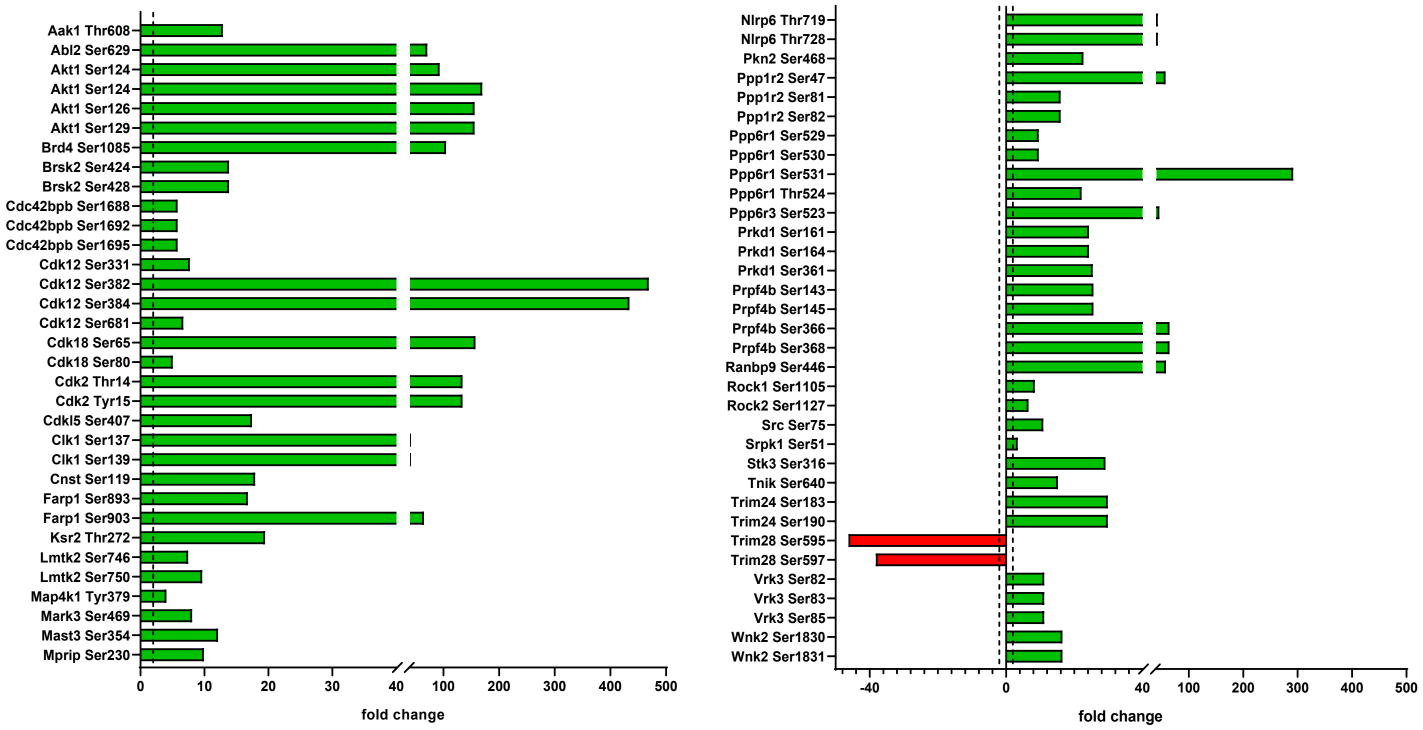

Figure S12. Quantitative changes in phosphoproteins involved in the regulation of MAP-mediated signaling in GH1 cells after after siRNA-mediated β-arrestin2 knockdown.

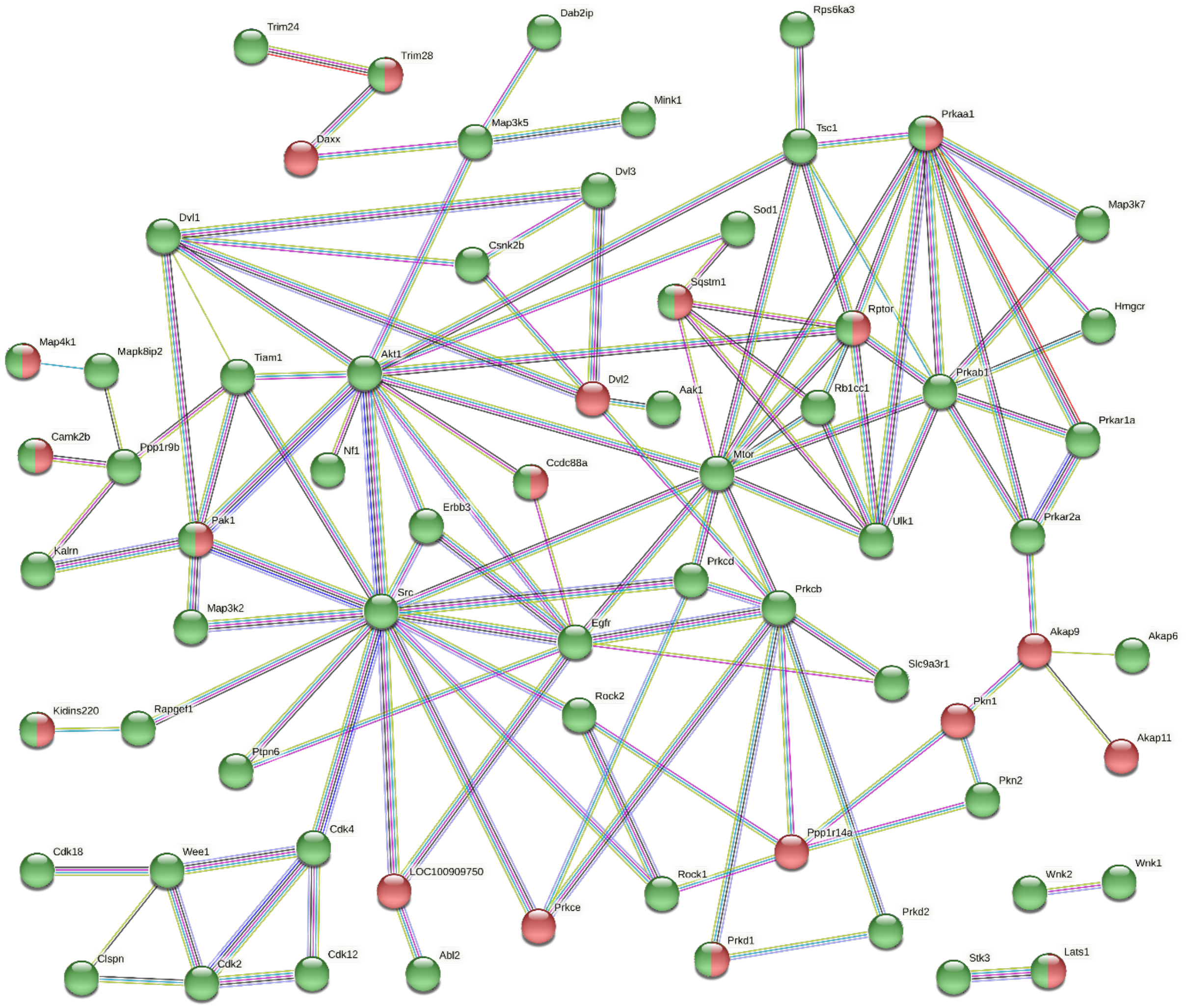

Figure S13. A network of differentially phosphorylated proteins involved in the regulation of MAP-mediated signaling in GH1 cells after siRNA-mediated β-arrestin2 knockdown. Elevated phosphorylation in green, decreased phosphorylation in red.

Table S7. A list of differentially phosphorylated proteins involved in MAP kinase-mediated signaling in GH1 cells after stimulation with 1 μM TRH

Uniprot ID	Gene ID	Gene name	Phosphorylated regions
F1LRI7	Aak1	AP2 associated kinase 1	Ser626 ↑[TRH] (_2)
F1LPB4	Akap9	A-kinase anchoring protein 9	Ser858 ↓[C] (_3), Thr864 ↓[C] (_3), Thr865 ↓[C] (_3), Ser1501 ↓[C] (_1)
F1LR81	Akap11	A-kinase anchoring protein 11	Ser434 ↓[C] (_3), Ser439 ↓[C] (_3), Ser440 ↓[C] (_3)
P47196	Akt1	AKT serine/threonine kinase 1	Ser126 ↓[C] (_1) ↑[TRH] (_2)
A0A0G2K3I0	Atm	ATM serine/threonine kinase	Tyr2839 ↓[C] (_1)
O70239	Axin1	axin 1	Ser75 ↓[C] (_3), Ser77 ↓[C] (_3), Thr79 ↓[C] (_3)
F1M9C3	Braf	B-Raf proto-oncogene, serine/threonine kinase	Ser94 ↓[C] (_1)
A0A0G2JYV0	Brd4	bromodomain containing 4	Ser1071 ↓[C] (_3), Ser1077 ↓[C] (_3)
D3ZML2	Brsk2	BR serine/threonine kinase 2	Ser424 ↓[C] (_2), Ser428 ↓[C] (_2) ↓[C] (_3), Ser436 ↓[C] (_3)
F1LNI8	Camk2b	calcium/calmodulin-dependent protein kinase II beta	Thr361 ↓[-2.68] (_3)
D3ZYD7	Ccdc88a	coiled coil domain containing 88A	Ser1319 ↓[C] (_1)
A1L1L5	Ccnk	cyclin K	Ser348 ↓[C] (_1)
P35426	Cdk4	cyclin-dependent kinase 4	Ser300 ↑[TRH] (_1)
A0A0G2K5U7	Cdk12	cyclin-dependent kinase 12	Ser273 ↓[C] (_2), Ser275 ↓[C] (_2), Ser332 ↓[C] (_2)
D3ZKK3	Cnst	consortin, connexin sorting protein	Ser117 ↓[C] (_1)
Q6MGC8	Daxx	death-domain associated protein	Ser526 ↓[-7.22] (_2), Ser550 ↑[TRH] (_3), Ser553 ↑[TRH] (_3), Ser556 ↑[TRH] (_3)
A0A0G2KB92	Dclk1	doublecortin-like kinase 1	Ser363 ↓[C] (_2), Ser364 ↓[C] (_2), Ser364 ↓[C] (_1)
F1LRS2	Dock7	dedicator of cytokinesis 7	Ser904 ↓[C] (_3), Ser906 ↓[C] (_2), Ser918 ↓[C] (_2)
Q9WVB9	Dvl1	dishevelled segment polarity protein 1	Ser194 ↑[TRH] (_1)
D4ADV8	Dvl3	dishevelled segment polarity protein 3	Ser125 ↓[C] (_1)
D4A0G9	Ercc6	ERCC excision repair 6, chromatin remodeling factor	Ser1014 ↓[C] (_1)
D3ZAL7	Gab1	GRB2-associated binding protein 1	Ser438 ↓[C] (_2)
A0A0G2K2T6	Ilf3	interleukin enhancer binding factor 3	Ser482 ↑[TRH] (_1)
Q9EQG6	Kidins220	kinase D-interacting substrate 220	Ser1585 ↓[C] (_2), Ser1673 ↓[C] (_1)
F1M2K4	Lats1	large tumor suppressor kinase 1	Ser1111 ↓[C] (_1)
D3ZBH5	Lmtk2	lemur tyrosine kinase 2	Ser496 ↓[C] (_2)
F1M0A6	LOC100909750	tyrosine-protein kinase ABL1-like	Ser798 ↓[C] (_3), Thr807 ↓[C] (_3)
A0A0G2K3R1	Map3k4	mitogen activated protein kinase kinase kinase 4	Ser59 ↓[C] (_3) ↓[C] (_2), Ser77 ↓[C] (_3) ↓[C] (_2)

D3Z8I4	Map4k1	mitogen activated protein kinase kinase kinase kinase 1	Ser370 ↓[C] (_2), Ser373 ↓[C] (_2), Ser375 ↓[C] (_2), Tyr379 ↓[C] (_2)
G3V9M2	Mapk8ip2	mitogen-activated protein kinase 8 interacting protein 2	Ser254 ↑[TRH] (_2), Ser257 ↓[C] (_1)
A0A0G2JSU4	Ndrg2	NDRG family member 2	Ser336 ↓[C] (_3)
P35465	Pak1	p21 (RAC1) activated kinase 1	Ser174 ↓[-5.65] (_1), Ser219 ↓[C] (_1), Thr228 ↓[C] (_1)
A0A0H2UHA0	Ppp1r2	protein phosphatase 1, regulatory (inhibitor) subunit 2	Ser81 ↓[C] (_3), Ser90 ↓[C] (_3)
D3ZG37	Ppp6r1	protein phosphatase 6, regulatory subunit 1	Thr524 ↓[C] (_2)
D3ZBT9	Ppp6r3	protein phosphatase 6, regulatory subunit 3	Thr518 ↓[C] (_3), Ser524 ↓[C] (_2), Ser525 ↓[C] (_2) ↑[TRH] (_3)
P54645	Prkaa1	protein kinase AMP-activated catalytic subunit alpha 1	Ser486 ↓[-13.17] (_2)
F1LMV8	Prkce	protein kinase C, epsilon	Ser140 ↓[-14.81] (_2)
A0A0G2K928	Prkd1	protein kinase D1	Ser189 ↓[C] (_2), Ser192 ↓[C] (_2), Ser361 ↓[C] (_2), Ser361 ↓[C] (_1), Thr364 ↓[C] (_2)
Q5RKH1	Prpf4b	pre-mRNA processing factor 4B	Ser21 ↓[C] (_3) ↑[TRH] (_2), Ser24 ↓[C] (_3) ↑[TRH] (_2), Ser33 ↓[C] (_3)
P70600	Ptk2b	protein tyrosine kinase 2 beta	Ser389 ↓[C] (_3), Ser392 ↓[C] (_3), Ser394 ↓[C] (_3), Ser396 ↓[C] (_3), Ser399 ↓[C] (_3)
F1LQT3	Rock2	Rho-associated coiled-coil containing protein kinase 2	Ser1124 ↑[TRH] (_2) ↑[TRH] (_1)
A0A0G2KB60	Rps6kc1	ribosomal protein S6 kinase C1	Ser603 ↓[C] (_2)
D3ZDU2	Rptor	regulatory associated protein of MTOR, complex 1	Thr857 ↑[TRH] (_2), Ser859 ↓[C] (_2)
G3V7X2	Scg2	secretogranin II	Ser491 ↑[TRH] (_1), Ser494 ↑[TRH] (_1), Ser495 ↓[-3.50] (_1)
G3V7I8	Slk	STE20-like kinase	Ser348 ↓[C] (_2)
E9PSJ4	Spag9	sperm associated antigen 9	Ser719 ↓[-14.77] (_2)
O08623	Sqstm1	sequestosome 1	Ser364 ↓[C] (_1)
E9PTN4	Srpk1	SRSF protein kinase 1	Ser33 ↓[C] (_3), Ser37 ↓[C] (_3), Ser39 ↓[C] (_3), Ser51 ↓[C] (_3) ↓[C] (_2), Thr453 ↓[C] (_2), Ser455 ↓[C] (_2)
A0A0G2K7Z9	Stk10	serine/threonine kinase 10	Thr 456 ↓[C] (_1)
F7ESX8	Trim24	tripartite motif-containing 24	Ser294 ↓[C] (_1)
O08629	Trim28	tripartite motif-containing 28	Ser27 ↑[TRH] (_2), Ser31 ↓[-18.62] (_2) ↓[C] (_1)
A0A0G2K3A0	Wnk1	WNK lysine deficient protein kinase 1	Ser1809 ↓[C] (_2)
D3ZMJ7	Wnk2	WNK lysine deficient protein kinase 2	Ser45 ↑[TRH] (_1), Ser49 ↓[C] (_1), Ser1830 ↓[C] (_2), Ser1831 ↓[C] (_2)
D4AE17	Zak	sterile alpha motif and leucine zipper containing kinase AZK	Ser434 ↓[C] (_2), Ser452 ↓[C] (_2)
A0A0G2K8T6	Zeb2	zinc finger E-box binding homeobox 2	Ser517 ↓[C] (_3), Thr522 ↓[C] (_3)

↑, elevated phosphorylation; ↓, decreased phosphorylation; ↓[C], detected only in control (negative siRNA-treated) cells; ↑[TRH]. detected only in cells stimulated with TRH; (_1, _2, _3), multiplicity

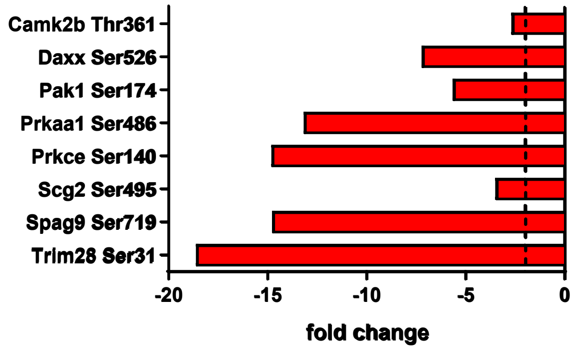

Figure S14. Quantitative changes in phosphoproteins involved in the regulation of MAP-mediated signaling in GH1 cells after stimulation with 1 μM TRH.

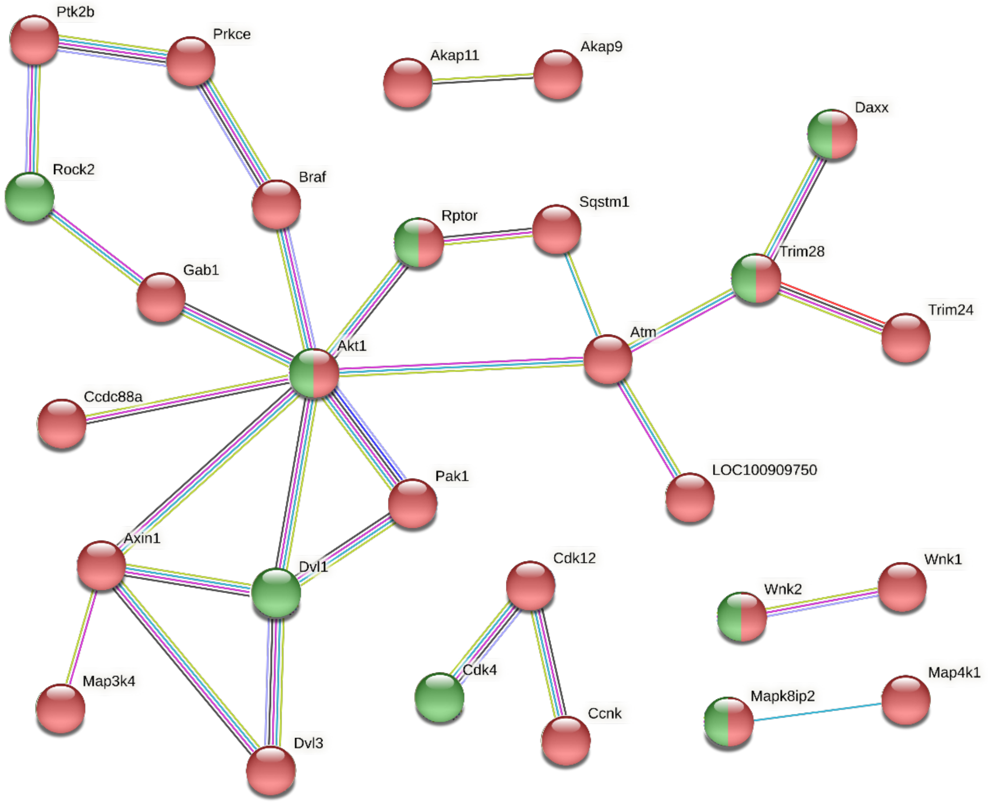

Figure S15. A network of differentially phosphorylated proteins involved in the regulation of MAP-mediated signaling in GH1 cells after TRH treatment. Elevated phosphorylation in green, decreased phosphorylation in red.

Table S8. A list of differentially phosphorylated proteins involved in MAP kinase-mediated signaling in GH1 cells after stimulation with 1 μM TAL

Uniprot ID	Gene ID	Gene name	Phosphorylated regions
F1LRI7	Aak1	AP2 associated kinase 1	Thr622 ↓[-125.55] (_2)
F1LPB4	Akap9	A-kinase anchoring protein 9	Ser858 ↓[C] (_3), Ser862 ↓[-6.60] (_2), Thr864 ↓[C] (_3), Thr865 ↓[-6.60] (_2) ↓[C] (_3), Ser1500 ↓[-10.14] (_1), Ser1501 ↓[-10.14] (_1)
P47196	Akt1	AKT serine/threonine kinase 1	Ser124 ↓[-5.22] (_1), Ser126 ↓[-363.47] (_1)
A0A0G2K3I0	Atm	ATM serine/threonine kinase	Tyr2839 ↓[C] (_1)
Q64542	Atp2b4	ATPase plasma membrane Ca2+ transporting 4	Ser1151 ↓[C] (_1)
O70239	Axin1	axin 1	Ser75 ↓[C] (_3), Ser77 ↓[C] (_3), Thr79 ↓[C] (_3)
F1M9C3	Braf	B-Raf proto-oncogene, serine/threonine kinase	Ser94 ↓[C] (_1)
A0A0G2JYV0	Brd4	bromodomain containing 4	Ser1071 ↓[C] (_3), Ser1077 ↓[C] (_3)
D3ZML2	Brsk2	BR serine/threonine kinase 2	Ser424 ↓[C] (_2), Ser428 ↓[C] (_3) ↓[C] (_2), Ser436 ↓[C] (_3)
A0A0G2K5C0	Camsap3	calmodulin regulated spectrin-associated protein family, member 3	Ser320 ↓[C] (_1) ↑[TAL] (_2)
D3ZYD7	Ccdc88a	coiled coil domain containing 88A	Ser1319 ↓[C] (_1)
A1L1L5	Ccnk	cyclin K	Ser348 ↓[C] (_1)
O70509	Cd44	CD44 molecule (Indian blood group)	Ser328 ↓[C] (_1)
A0A0G2K5U7	Cdk12	cyclin-dependent kinase 12	Ser273 ↓[C] (_2), Ser275 ↓[C] (_2), Ser331 ↓[-13.39] (_2), Ser332 ↓[-20.00] (_2), Ser1079 ↓[C] (_1)
D3ZKK3	Cnst	consortin, connexin sorting protein	Ser117 ↓[C] (_1), Ser119 ↓[-5.65] (_1)
A0A096MJD3	Csnk2b	casein kinase 2 beta	Ser158 ↓[C] (_1)
Q6MGC8	Daxx	death domain associated protein	Ser526 ↓[-3.62] (_2)
A0A0G2KB92	Dclk1	doublecortin-like kinase 1	Ser330 ↓[-12.50] (_2), Ser334 ↓[C] (_3), Ser337 ↓[C] (_2), Ser340 ↓[C] (_3), Ser363 ↓[C] (_2), Ser364 ↓[C] (_2)
F1LRS2	Dock7	dedicator of cytokinesis 7	Ser904 ↓[-5.08] (_2), Ser906 ↓[-5.08] (_2)
Q9WVB9	Dvl1	dishevelled segment polarity protein 1	Ser194 ↑[TAL] (_1)
D3ZB71	Dvl2	dishevelled segment polarity protein 2	Ser211 ↓[-8.27] (_1)
D4ADV8	Dvl3	dishevelled segment polarity protein 3	Ser125 ↓[-3.22] (_1)
M0R4L1	Erbb2	erb-b2 receptor tyrosine kinase 2	Thr152 ↓[-5.36] (_2), Ser154 ↓[-5.36] (_2)
D3ZS47	Ercc6	ERCC excision repair 6, chromatin remodeling factor	Ser405 ↓[-4.30] (_2), Ser406 ↓[-4.30] (_2)
F1LYQ8	Farp1	FERM, ARH/RhoGEF and pleckstrin domain protein 1	Ser893 ↓[C] (_2), Thr902 ↓[-14.79] (_2) ↓[-6.76] (_3)
A0A0G2JSH4	Gsk3b	glycogen synthase kinase 3 beta	Ser389 ↓[-3.03] (_1)
F1MAF1	Hsf1	heat shock transcription factor 1	Ser303 ↓[C] (_2), Ser307 ↓[C] (_2)
A0A0G2K2T6	Ilf3	interleukin enhancer binding factor 3	Ser482 ↑[TAL] (_1)

Q9EQG6	Kidins220	kinase D-interacting substrate 220	Ser1513 ↓[-2.03] (_2), Ser1518 ↓[-2.06] (_2), Ser1585 ↓[C] (_2), Ser1673 ↓[-9.93] (_1)
F1M2K4	Lats1	large tumor suppressor kinase 1	Ser1111 ↓[C] (_1)
D3ZBH5	Lmtk2	lemur tyrosine kinase 2	Ser496 ↓[C] (_2), Ser580 ↓[C] (_2), Ser1334 ↑[TAL] (_3)
F1M0A6	LOC100909750	tyrosine-protein kinase ABL1-like	Ser801 ↓[C] (_2)
A0A0G2K3R1	Map3k4	mitogen activated protein kinase kinase kinase 4	Ser59 ↓[C] (_3), Ser59 ↓[-11.89] (_2), Ser77 ↓[-11.89] (_2), Ser77 ↓[C] (_3)
P0C8E4	Map3k7	mitogen activated protein kinase kinase kinase 7	Ser439 ↓[-5.39] (_1)
D3Z8I4	Map4k1	mitogen activated protein kinase kinase kinase kinase 1	Ser370 ↓[C] (_2), Tyr379 ↓[C] (_2)
G3V9M2	Mapk8ip2	mitogen-activated protein kinase 8 interacting protein 2	Ser254 ↑[TAL] (_2), Ser257 ↓[-7.28] (_1)
F1M836	Mark3	microtubule affinity regulating kinase 3	Ser400 ↓[C] (_2), Ser469 ↓[-11.09] (_1)
Q810W7	Mast1	microtubule associated serine/threonine kinase 1	Ser346 ↓[-2.01] (_1)
D3ZL30	Mast3	microtubule associated serine/threonine kinase 3	Ser354 ↓[C] (_1)
A0A0G2JSU4	Ndrg2	NDRG family member 2	Ser336 ↓[C] (_3)
P35465	Pak1	p21 (RAC1) activated kinase 1	Ser219 ↓[-2.29] (_1)
Q64303	Pak2	p21 protein (Cdc42/Rac)-activated kinase 2	Ser2 ↓[-10.61] (_1)
A0A0H2UHA0	Ppp1r2	protein phosphatase 1, regulatory (inhibitor) subunit 2	Ser47 ↑[TAL] (_2) ↓[-5.98] (_1), Ser49 ↑[TAL] (_2), Ser81 ↓[C] (_3), Ser82 ↓[-7.88] (_1), Ser90 ↓[C] (_3), Ser135 ↓[C] (_1)
Q99MC0	Ppp1r14a	protein phosphatase 1, regulatory (inhibitor) subunit 14A	Ser136 ↓[-5.00] (_1)
D3ZG37	Ppp6r1	protein phosphatase 6, regulatory subunit 1	Thr524 ↓[-15.15] (_2), Ser529 ↓[-13.53] (_3) ↓[-13.05] (_2), Ser530 ↓[-13.53] (_3) ↓[-13.05] (_2), Ser531 ↓[-12.36] (_2), Ser827 ↓[-4.48] (_1)
D3ZBT9	Ppp6r3	protein phosphatase 6, regulatory subunit 3	Thr518 ↓[-4.76] (_3), Ser524 ↓[C] (_2), Ser525 ↓[C] (_2)
P54645	Prkaa1	protein kinase AMP-activated catalytic subunit alpha 1	Ser486 ↓[-6.03] (_2)
P12369	Prkar2b	protein kinase cAMP-dependent type 2 regulatory subunit beta	Ser83 ↓[C] (_1), Ser85 ↓[C] (_1)
A0A0G2K928	Prkd1	protein kinase D1	Ser161 ↓[-4.59] (_2), Ser164 ↓[-4.59] (_2), Ser189 ↓[C] (_2), Ser192 ↓[C] (_2), Ser361 ↓[C] (_1)
G3V7J2	Prkra	protein activator of interferon induced protein kinase EIF2AK2	Ser18 ↓[C] (_1)
Q5RKH1	Prpf4b	pre-mRNA processing factor 4B	Ser21 ↓[C] (_3) ↑[TAL] (_2), Ser24 ↓[C] (_3) ↑[TAL] (_2), Ser33 ↓[C] (_3), Ser143 ↓[-8.10] (_2), Ser145 ↓[-8.10] (_2)
P70600	Ptk2b	protein tyrosine kinase 2 beta	Ser389 ↓[C] (_3), Ser392 ↓[C] (_3), Ser394 ↓[C] (_3), Ser396 ↓[C] (_3), Ser399 ↓[C] (_3)
F1M8L9	Rapgef1	Rap guanine nucleotide exchange factor 1	Ser239 ↓[C] (_2)

F1LQT3	Rock2	Rho-associated coiled-coil containing protein kinase 2	Ser1124 ↑[TAL] (_2) ↑[TAL] (_1)
D3ZDU2	Rptor	regulatory associated protein of MTOR, complex 1	Ser859 ↓[-3.12] (_2)
F1LU97	Sash1	SAM and SH3 domain containing 1	Ser539 ↓[C] (_2), Ser541 ↓[C] (_2)
G3V7X2	Scg2	secretogranin II	Ser495 ↓[-4.13] (_1)
E9PSJ4	Spag9	sperm associated antigen 9	Ser719 ↓[-8.71] (_2)
O08623	Sqstm1	sequestosome 1	Ser354 ↑[TAL] (_2), Ser364 ↓[C] (_1)
E9PTN4	Srpk1	SRSF protein kinase 1	Ser33 ↓[-10.08] (_3), Ser37 ↓[-10.08] (_3), Ser39 ↓[-7.44] (_3), Ser51 ↓[-4.50] (_2) ↓[-10.08] (_3), Thr453 ↓[C] (_2), Ser455 ↓[C] (_2)
B1WBQ5	Stk3	serine/threonine kinase 3	Ser316 ↓[-13.41] (_1)
A0A0G2K7Z9	Stk10	serine/threonine kinase 10	Thr456 ↓[-4.56] (_1)
B0LT89	Stk24	serine/threonine kinase 24	Ser4 ↓[-13.05] (_1)
D3ZZQ0	Tnik	TRAF2 and NCK interacting kinase	Ser640 ↓[-10.91] (_1), Ser764 ↓[-5.33] (_2), Ser769 ↓[-5.33] (_2)
F7ESX8	Trim24	tripartite motif-containing 24	Ser294 ↓[C] (_1), Ser320 ↓[-12.00] (_1)
O08629	Trim28	tripartite motif-containing 28	Ser31 ↓[-5.71] (_1), Ser474 ↓[C] (_1), Ser595 ↓[-53.08] (_3), Ser597 ↓[-28.00] (_3)
Q66HC2	Vrk3	vaccinia related kinase 3	Ser82 ↓[C] (_3), Ser83 ↓[C] (_3), Ser85 ↓[C] (_3), Thr88 ↓[C] (_3)
A0A0G2K3A0	Wnk1	WNK lysine deficient protein kinase 1	Ser1809 ↓[C] (_2)
D3ZMJ7	Wnk2	WNK lysine deficient protein kinase 2	Ser49 ↓[C] (_1)
D4AE17	Zak	sterile alpha motif and leucine zipper containing kinase AZK	Ser434 ↓[C] (_2), Ser452 ↓[C] (_2)

↑, elevated phosphorylation; ↓, decreased phosphorylation; ↓[C], detected only in control (negative siRNA-treated) cells; ↑[TAL]. detected only in cells stimulated with TAL; (_1, _2, _3), multiplicity

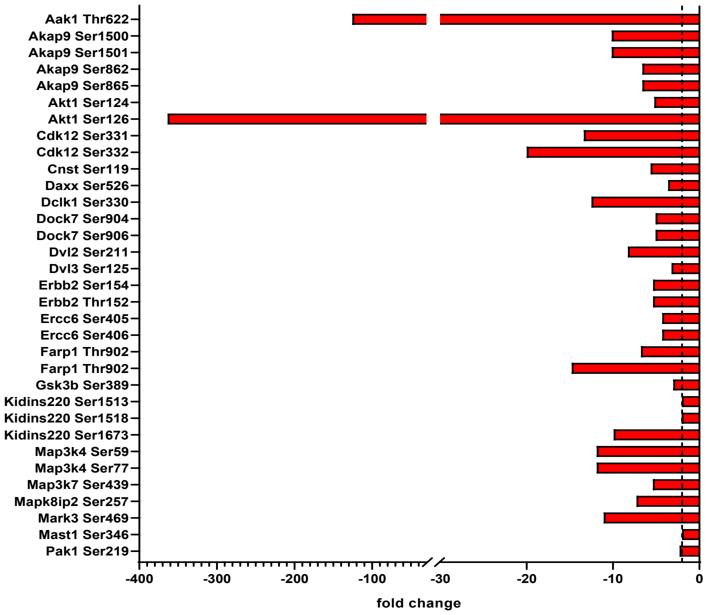

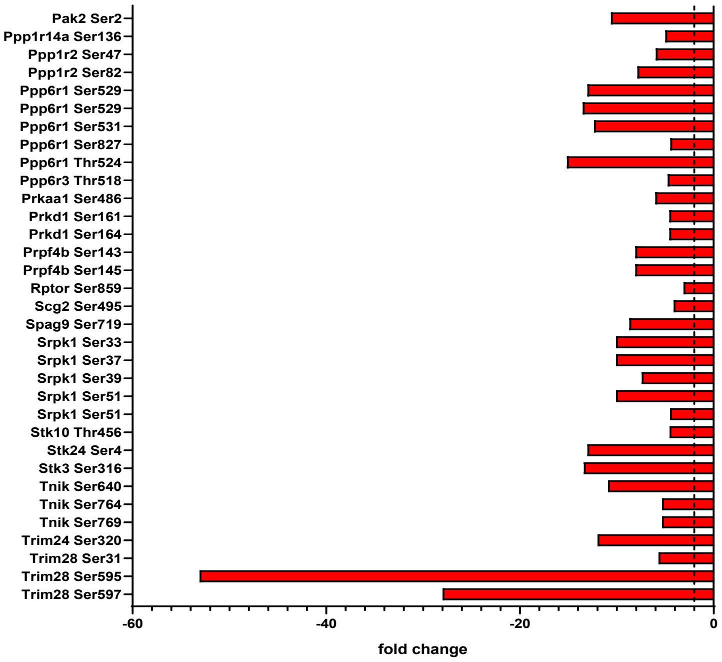

Figure S16. Quantitative changes in phosphoproteins involved in the regulation of MAP-mediated signaling iin GH1 cells after TAL treatment.

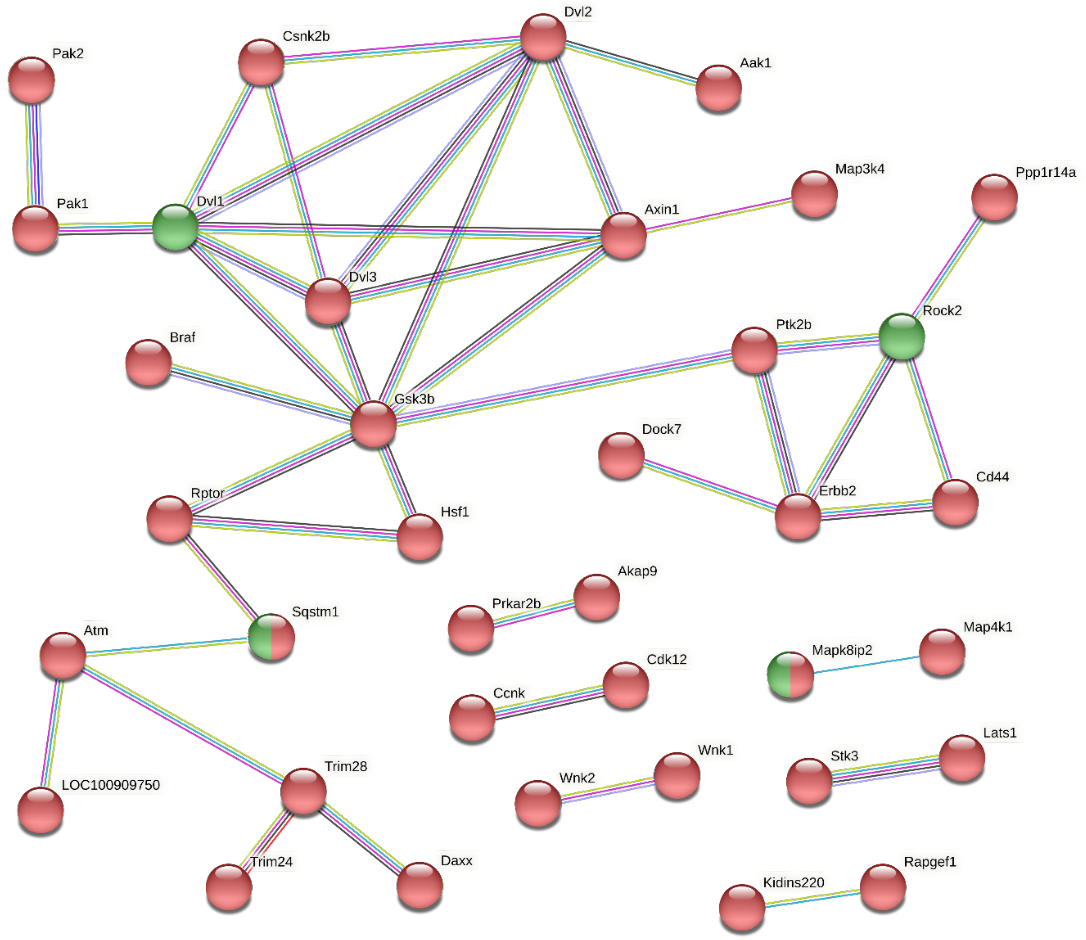

Figure S17. A network of differentially phosphorylated proteins involved in the regulation of MAP-mediated signaling in in GH1 cells after TAL treatment. Elevated phosphorylation in green, decreased phosphorylation in red.

Table S9. A list of differentially phosphorylated proteins involved in MAP kinase-mediated signaling in GH1 cells after siRNA-mediated β-arrestin2 knockdown and stimulation with 1 μM TRH

Uniprot ID	Gene ID	Gene name	Phosphorylated regions
F1LRI7	Aak1	AP2 associated kinase 1	Ser626 ↑[TRH] (_2)
G3V6M0	Akap6	A-kinase anchoring protein 6	Ser2208 ↓[AR] (_2)
F1LR81	Akap11	A-kinase anchoring protein 11	Ser434 ↑[TRH] (_3), Ser439 ↑[TRH] (_3), Ser440 ↑[TRH] (_3)
F1M3G7	Akap13	A-kinase anchoring protein 13	Ser2676 ↑[TRH] (_2)
P47196	Akt1	AKT serine/threonine kinase 1	Ser124 ↓[AR] (_2), Ser126 ↓[AR] (_2)
Q63484	Akt3	AKT serine/threonine kinase 3	Ser2 ↓[AR] (_2), Thr5 ↓[AR] (_2)
F1M7M4	Bmp2k	BMP-2 inducible kinase	Ser934 ↑[TRH] (_3), Ser936 ↑[TRH] (_3), Ser937 ↑[TRH] (_3)
A0A0G2JYV0	Brd4	bromodomain containing 4	Ser1071 ↓[AR] (_3), Ser1077 ↓[AR] (_3), Ser1085 ↓[AR] (_3)
D3ZML2	Brsk2	BR serine/threonine kinase 2	Ser428 ↑[TRH] (_3), Ser436 ↑[TRH] (_3)
F1LNI8	Camk2b	calcium/calmodulin-dependent protein kinase II beta	Ser358 ↑[TRH] (_2), Thr361 ↓[AR] (_3)
A0A0G2K5C0	Camsap3	calmodulin regulated spectrin-associated protein family, member 3	Ser320 ↑[TRH] (_2)
D3ZYD7	Ccdc88a	coiled coil domain containing 88A	Ser1319 ↑[TRH] (_1), Ser1343 ↓[AR] (_1)
D4A3G2	Cdk11b	cyclin-dependent kinase 11B	Tyr751 ↓[AR] (_2)
A0A140UHY2	Cdk16	cyclin-dependent kinase 16	Ser43 ↑[TRH] (_2), Ser60 ↑[TRH] (_2)
D3ZHC3	Clspn	claspin	Ser77 ↓[AR] (_3)
D3ZKK3	Cnst	consortin, connexin sorting protein	Ser117 ↑[TRH] (_1)
Q63768	Crk	CRK proto-oncogene, adaptor protein	Ser41 ↑[TRH] (_1)
Q6MGC8	Daxx	death-domain associated protein	Ser521 ↑[TRH] (_2), Ser526 ↑[TRH] (_2)
A0A0G2KB92	Dclk1	doublecortin-like kinase 1	Ser334 ↓[AR] (_3), Ser340 ↓[AR] (_3) ↑[TRH] (_1)
F1LMD9	Gak	cyclin G associated kinase	Ser824 ↑[TRH] (_2), Ser827 ↑[TRH] (_2)
D3ZUP4	Gpatch2l	G patch domain containing 2-like	Ser86 ↑[TRH] (_2), Ser88 ↑[TRH] (_2)
A0A0G2JSH4	Gsk3b	glycogen synthase kinase 3 beta	Ser389 ↓[AR] (_1)
P51639	Hmgcr	3-hydroxy-3-methylglutaryl-CoA reductase	Ser356 ↓[AR] (_2)
A0A0G2K2T6	Ilf3	interleukin enhancer binding factor 3	Thr67 ↓[AR] (_1)
Q9EQG6	Kidins220	kinase D-interacting substrate 220	Ser1513 ↑[TRH] (_2), Ser1518 ↑[TRH] (_2)
F1M2K4	Lats1	large tumor suppressor kinase 1	Ser1111 ↑[TRH] (_1)
D3ZBH5	Lmtk2	lemur tyrosine kinase 2	Ser576 ↓[AR] (_2), Ser746 ↑[TRH] (_1), Ser756 ↓[AR] (_2) ↓[AR] (_1), Ser1035 ↓[AR] (_1), Ser1524 ↓[AR] (_2)
F1M0A6	LOC100909750	tyrosine-protein kinase ABL 1-like	Ser798 ↑[TRH] (_3)
F1M9D0	Map3k2	mitogen activated protein kinase kinase kinase 2	Ser135 ↑[TRH] (_1), Thr337 ↓[AR] (_2)
A0A0G2K3R1	Map3k4	mitogen activated protein kinase kinase kinase 4	Ser59 ↓[AR] (_2)

P0C8E4	Map3k7	mitogen activated protein kinase kinase kinase 7	Ser454 ↑[TRH] (_2)
A0A0G2JUN9	Map3k9	mitogen-activated protein kinase kinase kinase 9	Ser541 ↓[AR] (_2)
G3V9M2	Mapk8ip2	mitogen-activated protein kinase 8 interacting protein 2	Ser254 ↓[AR] (_2)
A0A0G2K6X6	Mark2	microtubule affinity regulating kinase 2	Ser567 ↑[TRH] (_1)
Q810W7	Mast1	microtubule associated serine/threonine kinase 1	Ser346 ↓[AR] (_1)
A0A0G2JSU4	Ndrg2	NDRG family member 2	Ser336 ↑[TRH] (_3)
P35465	Pak1	p21 (RAC1) activated kinase 1	Thr184 ↓[AR] (_1), Ser219 ↓[AR] (_1), Ser222 ↓[AR] (_1)
D3ZQ51	Pak6	p21 (RAC1) activated kinase 6	Ser328 ↓[AR] (_1)
Q63433	Pkn1	protein kinase N1	Ser920 ↑[TRH] (_1)
A0A0H2UHA0	Ppp1r2	protein phosphatase 1, regulatory (inhibitor) subunit 2	Ser47 ↓[AR] (_1), Ser82 ↓[AR] (_1), Ser135 ↓[AR] (_1)
D3ZBT9	Ppp6r3	protein phosphatase 6, regulatory subunit 3	Ser524 ↓[AR] (_2), Ser525 ↓[AR] (_2)
P54645	Prkaa1	protein kinase AMP-activated catalytic subunit alpha 1	Thr526 ↑[TRH] (_2)
A0A0G2K5Q0	Prkcb	protein kinase C, beta	Ser643 ↑[TRH] (_2)
F1LMV8	Prkce	protein kinase C, epsilon	Ser140 ↑[TRH] (_2), Ser148 ↑[TRH] (_2)
A0A0G2K928	Prkd1	protein kinase D1	Ser361 ↑[TRH] (_2), Thr364 ↑[TRH] (_2)
Q5RKH1	Prpf4b	pre-mRNA processing factor 4B	Ser21 ↑[TRH] (_3), Ser24 ↑[TRH] (_3), Ser33 ↑[TRH] (_3)
P97887	Psen1	presenilin 1	Ser366 ↑[TRH] (_2), Ser368 ↑[TRH] (_3), Thr371 ↑[TRH] (_3), Ser372 ↑[TRH] (_3)
Q66HJ7	Ptpra	protein tyrosine phosphatase, receptor type, A	Tyr465 ↑[TRH] (_1)
F1LVV3	Ranbp9	RAN binding protein 9	Ser459 ↓[AR] (_1)
F1LQT3	Rock2	Rho-associated coiled-coil containing protein kinase 2	Ser1124 ↓[AR] (_1) ↓[AR] (_2)
D3Z8E0	Rps6ka3	ribosomal protein S6 kinase A3	Thr365 ↑[TRH] (_2), Ser369 ↑[TRH] (_2)
D3ZDU2	Rptor	regulatory associated protein of MTOR, complex 1	Thr857 ↓[AR] (_2), Ser859 ↑[TRH] (_2)
F1LU97	Sash1	SAM and SH3 domain containing 1	Ser534 ↓[AR] (_2), Ser541 ↑[TRH] (_2)
G3V7X2	Scg2	secretogranin II	Ser491 ↑[TRH] (_1), Ser494 ↑[TRH] (_1)
Q9JJ19	Slc9a3r1	SLC9A3 regulator 1	Ser287 ↓[AR] (_1)
Q9WUD9	Src	SRC proto-oncogene, non-receptor tyrosine kinase	Ser75 ↓[AR] (_1)
E9PTN4	Srpk1	SRSF protein kinase 1	Ser51 ↓[AR] (_2)
A0A0G2JX62	Srpk2	SRSF protein kinase 2	Ser310 ↓[AR] (_1)
D3ZWV8	Tiam1	T-cell lymphoma invasion and metastasis 1	Ser1462 ↓[AR] (_2)
F7ESX8	Trim24	tripartite motif-containing 24	Ser293 ↑[TRH] (_1)
O08629	Trim28	tripartite motif-containing 28	Ser27 ↑[8.11] (_2), Ser595 ↓[AR] (_2) ↑[59.54] (_3)

Q9Z136	Tsc1	tuberous sclerosis 1	Ser561 ↓[AR] (_2), Ser565 ↓[AR] (_2)
D3ZN60	Ttbk2	tau tubulin kinase 2	Ser755 ↓[AR] (_2)
Q66HC2	Vrk3	vaccinia related kinase 3	Ser82 ↓[AR] (_3), Ser83 ↓[AR] (_3), Ser85 ↓[AR] (_3)
D3ZMJ7	Wnk2	WNK lysine deficient protein kinase 2	Ser49 ↓[AR] (_1)

↑, elevated phosphorylation; ↓, decreased phosphorylation; ↓[AR]. detected only in β-arrestin2-deficient cells; ↑[TRH], detected only in β-arrestin2-deficient cells stimulated with TRH; (_1, _2, _3), multiplicity

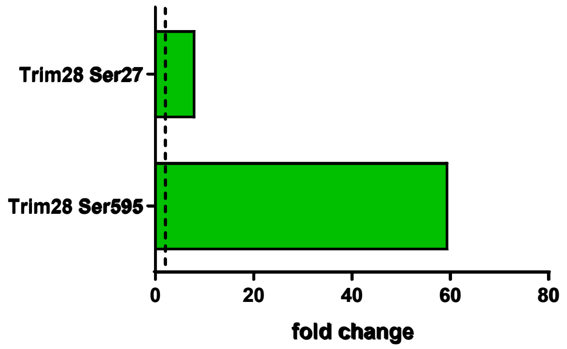

Figure S18. Quantitative changes in phosphoproteins involved in the regulation of MAP-mediated signaling in β-arrestin2-deficient GH1 cells after TRH treatment.

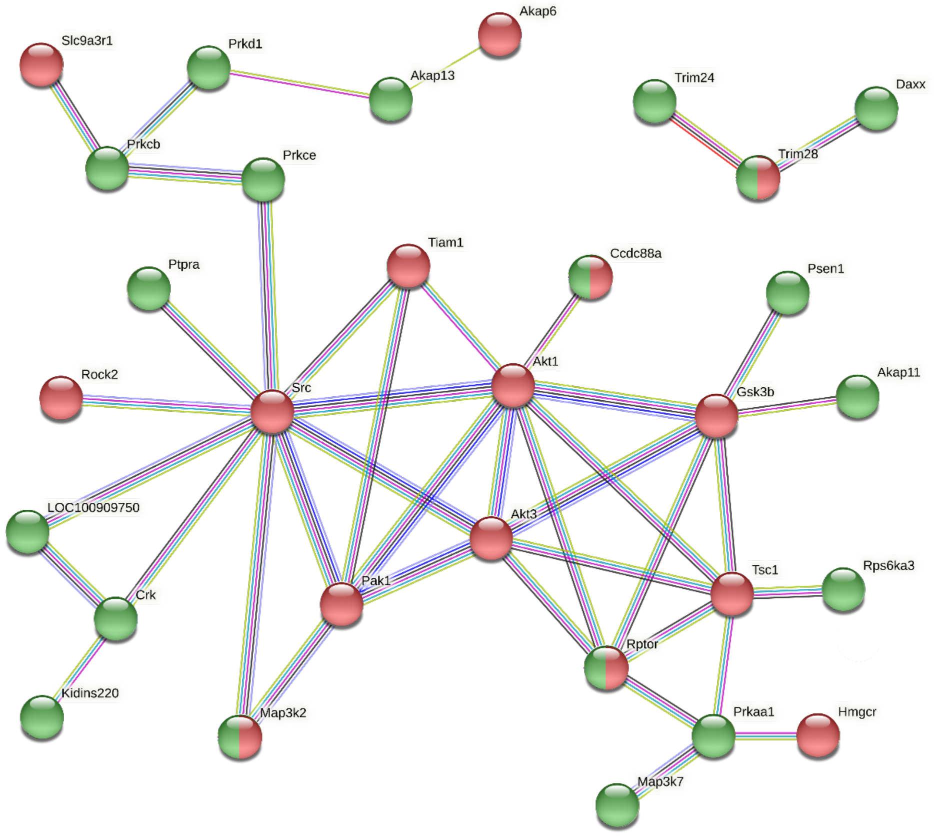

Figure S19. A network of differentially phosphorylated proteins involved in the regulation of MAP-mediated signaling in β-arrestin2-deficient GH1 cells after TRH treatment. Elevated phosphorylation in green, decreased phosphorylation in red.

Table S10. A list of differentially phosphorylated proteins involved in MAP kinase-mediated signaling in GH1 cells after siRNA-mediated β-arrestin2 knockdown and stimulation with 1 μM TAL

Uniprot ID	Gene ID	Gene name	Phosphorylated regions
F1M0N1	Abl2	ABL proto-oncogene 2, non receptor tyrosine kinase	Ser629 ↑[AR] (_1)
G3V6M0	Akap6	A-kinase anchoring protein 6	Ser423 ↓[AR] (_1), Ser2208 ↓[AR] (_2)
F1LPB4	Akap9	A-kinase anchoring protein 9	Ser1500 ↑[TAL] (_1), Ser1501 ↓[AR] (_1)
F1LR81	Akap11	A-kinase anchoring protein 11	Ser434 ↑[TAL] (_3), Ser439 ↑[TAL] (_3), Ser440 ↑[TAL] (_3)
F1M3G7	Akap13	A-kinase anchoring protein 13	Ser2676 ↑[TAL] (_2)
P47196	Akt1	AKT serine/threonine kinase 1	Ser122 ↓[AR] (_3), Ser126 ↓[AR] (_2) ↓[AR] (_1)
Q63484	Akt3	AKT serine/threonine kinase 3	Ser2 ↓[AR] (_2), Thr5 ↓[AR] (_2)
B2DD29	Brsk1	BR serine/threonine kinase 1	Ser490 ↓[AR] (_1)
D3ZML2	Brsk2	BR serine/threonine kinase 2	Ser424 ↓[-3.60] (_2), Ser428 ↑[TAL] (_3) ↓[-3.60] (_2), Ser436 ↑[TAL] (_3), Ser490 ↓[AR] (_1)
F1LNI8	Camk2b	calcium/calmodulin-dependent protein kinase II beta	Ser358 ↑[TAL] (_2)
A0A0G2K5C0	Camsap3	calmodulin regulated spectrin-associated protein family, member 3	Ser320 ↑[TAL] (_1)
D3ZYD7	Ccdc88a	coiled coil domain containing 88A	Ser1319 ↑[TAL] (_1), Ser1343 ↓[AR] (_1)
A0A0G2K5Z1	Cdc42bpa	CDC42 binding protein kinase alpha	Ser1622 ↓[AR] (_2), Ser1625 ↓[AR] (_2)
A0A140UHY2	Cdk16	cyclin-dependent kinase 16	Ser43 ↑[TAL] (_2), Ser60 ↑[TAL] (_2)
D3ZKK3	Cnst	consortin, connexin sorting protein	Ser117 ↑[TAL] (_1), Ser119 ↓[AR] (_1)
A0A096MJD3	Csnk2b	casein kinase 2 beta	Ser154 ↓[AR] (_1)
A0A0G2JTF2	Dab2ip	DAB2 interacting protein	Ser719 ↓[AR] (_1)
Q6MGC8	Daxx	death-domain associated protein	Ser521 ↑[TAL] (_2), Ser526 ↑[TAL] (_2), Ser550 ↑[TAL] (_3), Ser553 ↑[TAL] (_3), Ser556 ↑[TAL] (_3)
A0A0G2KB92	Dclk1	doublecortin-like kinase 1	Thr336 ↓[AR] (_2), Ser340 ↑[TAL] (_1)
G3V6K6	Egfr	epidermal growth factor receptor	Ser1165 ↓[AR] (_1)
F1LMD9	Gak	cyclin G associated kinase	Ser824 ↑[TAL] (_2), Ser827 ↑[TAL] (_2)
D3ZUP4	Gpatch2l	G patch domain containing 2-like	Ser86 ↑[TAL] (_2), Ser88 ↑[TAL] (_2)
P51639	Hmgcr	3-hydroxy-3-merhylglutaryl-CoA reductase	Ser356 ↓[AR] (_2)
A0A0G2K2T6	Ilf3	interleukin enhancer binding factor 3	Thr67 ↓[AR] (_1), Ser482 ↑[TAL] (_1)
Q9EQG6	Kidins220	kinase D-interacting substrate 220	Ser1351 ↑[TAL] (_2), Ser1353 ↑[TAL] (_2), Ser1513 ↑[TAL] (_2), Ser1518 ↑[TAL] (_2), Thr1520 ↓[AR] (_2), Ser1673 ↓[AR] (_1)

M0RBD3	Ksr2	kinase suppressor of ras 2	Thr272 ↓[AR] (_2)
F1M2K4	Lats1	large tumor suppressor kinase 1	Ser1111 ↑[TAL] (_1)
D3ZBH5	Lmtk2	lemur tyrosine kinase 2	Ser746 ↑[TAL] (_1), Ser756 ↓[AR] (_2), Ser1035 ↓[AR] (_1)
F1M0A6	LOC100909750	tyrosine-protein kinase ABL1-like	Ser801 ↑[TAL] (_2)
D4A930	Maml1	mastermind-like transcriptional coactivator 1	Ser88 ↓[AR] (_1)
Q62925	Map3k1	mitogen-activated protein kinase kinase kinase 1	Ser513 ↑[TAL] (_1)
F1M9D0	Map3k2	mitogen activated protein kinase kinase kinase 2	Ser135 ↑[TAL] (_1)
A0A0G2K3R1	Map3k4	mitogen activated protein kinase kinase kinase 4	Ser59 ↓[AR] (_3), Ser77 ↓[AR] (_3)
D3ZW27	Map3k5	mitogen-activated protein kinase kinase kinase 5	Ser1027 ↓[AR] (_2), Ser1031↓[AR] (_2)
P0C8E4	Map3k7	mitogen activated protein kinase kinase kinase 7	Ser454 ↑[TAL] (_2)
D3Z8I4	Map4k1	mitogen activated protein kinase kinase kinase kinase 1	Ser373 ↑[TAL] (_2), Ser375 ↑[TAL] (_2)
F1M754	Map4k4	mitogen-activated protein kinase kinase kinase kinase 4	Ser852 ↑[TAL] (_1)
G3V9M2	Mapk8ip2	mitogen-activated protein kinase 8 interacting protein 2	Ser259 ↑[TAL] (_2)
A0A0G2K6X6	Mark2	microtubule affinity regulating kinase 2	Ser392 ↑[TAL] (_1), Ser567 ↑[TAL] (_1)
D4A355	Mastl	microtubule associated serine/threonine kinase-like	Ser588 ↑[TAL] (_1)
A0A0G2K382	Mink1	misshapen-like kinase 1	Ser553 ↑[TAL] (_2), Ser559 ↑[TAL] (_2)
A0A0G2JSU4	Ndrg2	NDRG family member 2	Ser336 ↑[TAL] (_3)
A0A0G2K5C7	Nek1	NIMA-related kinase 1	Ser647 ↓[AR] (_1)
P35465	Pak1	p21 (RAC1) activated kinase 1	Thr228 ↑[TAL] (_1), Thr229 ↓[AR] (_2)
Q63433	Pkn1	protein kinase N1	Ser536 ↓[AR] (_2), Ser920 ↑[TAL] (_1)
A0A0G2K6J2	Pkn2	protein kinase N2	Ser467 ↑[TAL] (_1)
D3ZG37	Ppp6r1	protein phosphatase 6, regulatory subunit 1	Ser529 ↑[TAL] (_2), Ser530 ↑[TAL] (_2)
P54645	Prkaa1	protein kinase AMP-activated catalytic subunit alpha 1	Thr488 ↓[AR] (_2), Thr526 ↑[TAL] (_2), Ser527 ↑[TAL] (_3)
A0A0G2K5Q0	Prkcb	protein kinase C, beta	Ser643 ↑[TAL] (_2)
F1LMV8	Prkce	protein kinase C, epsilon	Ser140 ↑[TAL] (_2), Ser148 ↑[TAL] (_2)
A0A0G2K928	Prkd1	protein kinase D1	Ser361 ↑[TAL] (_2), Thr364 ↑[TAL] (_2)
Q5RKH1	Prpf4b	pre-mRNA processing factor 4B	Ser21 ↑[TAL] (_3), Ser24 ↑[TAL] (_3), Ser33 ↑[TAL] (_3)
P97887	Psen1	presenilin 1	Ser368 ↑[TAL] (_3), Thr371 ↑[TAL] (_3), Ser372 ↑[TAL] (_3)
P70600	Ptk2b	protein tyrosine kinase 2 beta	Ser389 ↓[AR] (_3), Ser392 ↓[AR] (_3), Ser394 ↓[AR] (_3), Ser396 ↓[AR] (_3), Ser399 ↓[AR] (_3)
F1LVV3	Ranbp9	RAN binding protein 9	Ser459 ↓[AR] (_1)
Q5I0I1	Riok2	RIO kinase 2	Ser437 ↑[TAL] (_1)
F1LQT3	Rock2	Rho-associated coiled-coil containing protein kinase 2	Ser1124 ↓[AR] (_1)
D3ZDU2	Rptor	regulatory associated protein of MTOR, complex 1	Thr857 ↓[AR] (_2), Ser859 ↑[TAL] (_2)

F1LU97	Sash1	SAM and SH3 domain containing 1	Ser534 ↓[AR] (_2), Ser541 ↑[TAL] (_2)
G3V7X2	Scg2	secretogranin II	Ser494 ↑[TAL] (_1), Ser495 ↑[TAL] (_1)
Q9JJ19	Slc9a3r1	SLC9A3 regulator 1	Ser277 ↑[TAL] (_1), Ser287 ↓[AR] (_1)
G3V7I8	Slk	STE20-like kinase	Ser647 ↑[TAL] (_2)
O08623	Sqstm1	sequestosome 1	Thr266 ↑[TAL] (_2)
Q9WUD9	Src	SRC proto-oncogene, non-receptor tyrosine kinase	Ser75 ↓[AR] (_1)
E9PTN4	Srpk1	SRSF protein kinase 1	Ser51 ↓[AR] (_2), Ser311 ↑[TAL] (_1)
A0A0G2JX62	Srpk2	SRSF protein kinase 2	Ser310 ↓[AR] (_1)
F1LRI6	Taok3	TAO kinase 3	Ser324 ↓[AR] (_1)
D3ZWV8	Tiam1	T-cell lymphoma invasion and metastasis 1	Ser725 ↓[AR] (_1), Ser1462 ↓[AR] (_2)
O08629	Trim28	tripartite motif-containing 28	Ser27 ↑[6.73] (_2), Ser31 ↓[AR] (_1), Ser595 ↑[54.59] (_3), Ser597 ↑[53.85] (_3)
Q9Z136	Tsc1	tuberous sclerosis 1	Ser561 ↓[AR] (_2), Ser565 ↓[AR] (_2), Ser1097 ↑[TAL] (_1)
D3ZN60	Ttbk2	tau tubulin kinase 2	Ser755 ↓[AR] (_2)
Q66HC2	Vrk3	vaccinia related kinase 3	Ser82 ↓[-12.85] (_3), Ser83 ↓[-12.85] (_3), Ser85 ↓[-12.85] (_3)
Q63802	Wee1	WEE1 G2 checkpoint kinase	Ser85 ↓[AR] (_2)
A0A0G2K3A0	Wnk1	WNK lysine deficient protein kinase 1	Ser1809 ↓[AR] (_2)
D3ZMJ7	Wnk2	WNK lysine deficient protein kinase 2	Ser1830 ↓[AR] (_2), Ser1831 ↓[AR] (_2)
A0A1W2Q6C5	Wnk3	WNK lysine deficient protein kinase 3	Ser436 ↓[AR] (_3), Thr449 ↓[AR] (_3)

↑, elevated phosphorylation; ↓, decreased phosphorylation; ↓[AR]. detected only in β-arrestin2-deficient cells; ↑[TAL], detected only in β-arrestin2-deficient cells stimulated with TAL; (_1, _2, _3), multiplicity

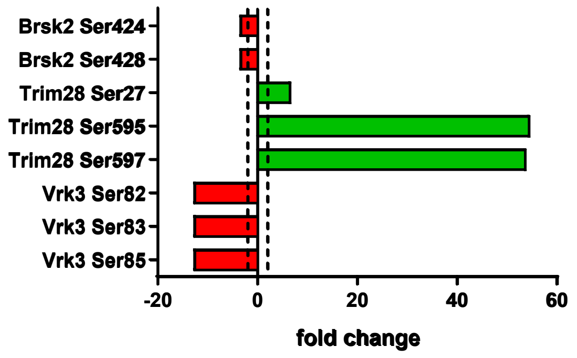

Figure S20. Quantitative changes in phosphoproteins involved in the regulation of MAP-mediated signaling in GH1 cells after siRNA-mediated β-arrestin2 knockdown and stimulation with 1 µM TAL.

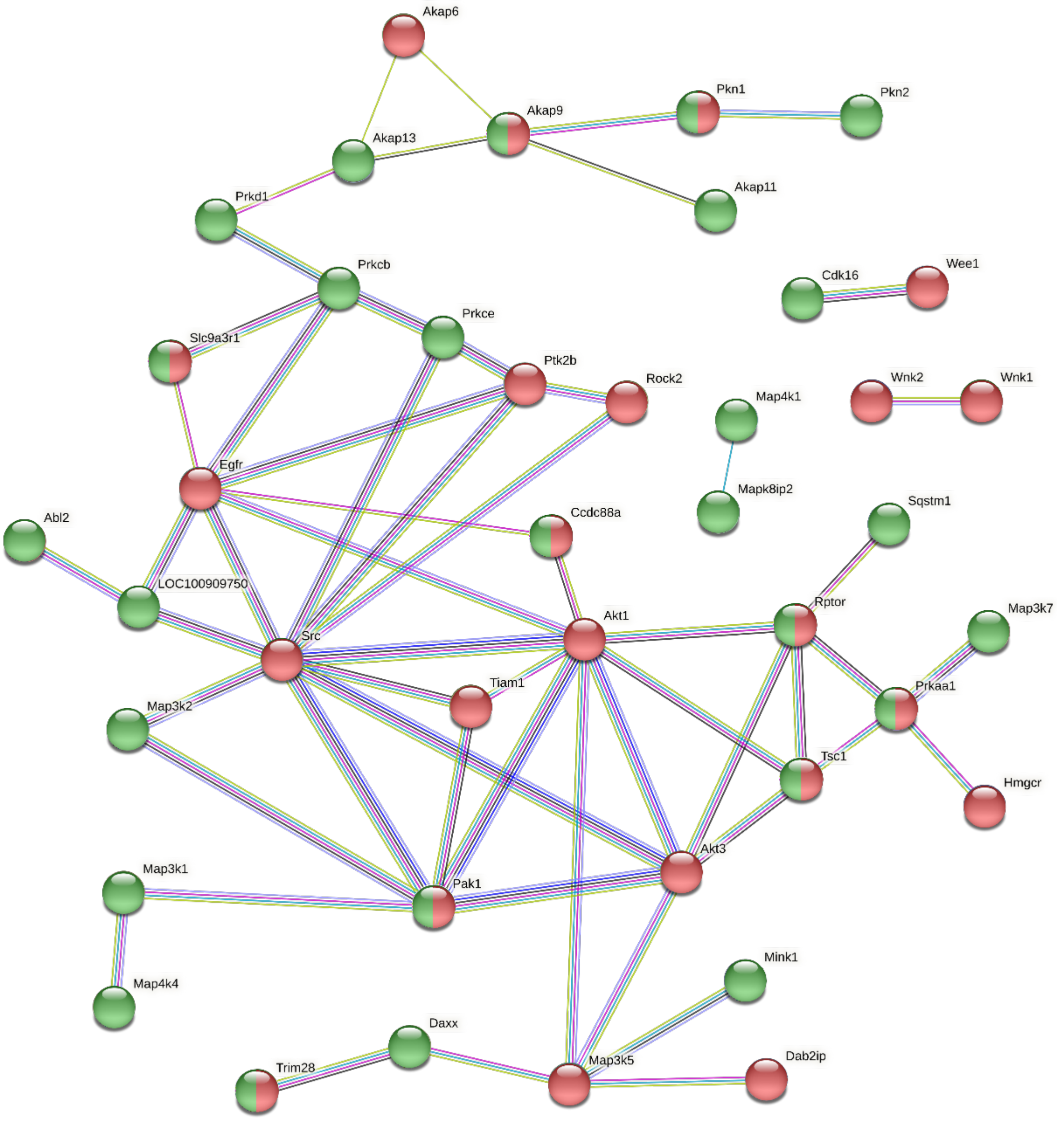

Figure S21. A network of differentially phosphorylated proteins involved in the regulation of MAP-mediated signaling in β-arrestin2-deficient GH1 cells after TAL treatment. Elevated phosphorylation in green, decreased phosphorylation in red.
